# Comparative analysis of glucagon-like peptide-1 receptor agonists, metformin, and inositol in improving anthropometric and metabolic outcomes in women with polycystic ovary syndrome: a network meta-analysis

**DOI:** 10.3389/fendo.2026.1833904

**Published:** 2026-07-03

**Authors:** Asad Omarion, Yaman Ayasa, Zainab Omarion, Batoul Jayouse, Hazem Ayesh

**Affiliations:** 1 Faculty of Medicine, Al-Quds University, Jerusalem, Palestine; 2 Deaconess Clinic Endocrinology, Deaconess Health System, Evansville, IN, United States

**Keywords:** GLP-1 receptor agonists, insulin resistance, metformin, myoinositol, network meta-analysis, obesity, polycystic ovary syndrome

## Abstract

**Background:**

Polycystic Ovary Syndrome (PCOS) is a common endocrine disorder characterized by obesity, insulin resistance, and cardiometabolic risks. Lifestyle intervention is first-line therapy, but drug therapy is often necessary. Common treatments include metformin, myoinositol, and glucagon-like peptide-1 (GLP-1) receptor agonists. The comparative metabolic efficacy of these treatments remains unclear.

**Methods:**

We conducted a systematic review and network meta-analysis (NMA) of randomized controlled trials (RCTs) according to PRISMA-NMA guidelines. Eligible studies recruited non-menopausal women with PCOS and evaluated GLP-1 receptor agonists, metformin, or myoinositol (± folic acid [FA]) alone or in combination versus placebo or active comparators. The primary outcome was body weight change, while secondary outcomes included body mass index (BMI), waist circumference, and Homeostasis Model Assessment for Insulin Resistance (HOMA-IR). Outcomes were synthesized using a frequentist random-effects NMA, and the CINeMA framework was used to assess certainty.

**Results:**

Sixteen RCTs were included. GLP-1 + metformin was the most effective intervention for weight reduction (MD −5.58 kg; 95% CI −8.57 to −2.59) and BMI decrease (MD −2.17; 95% CI −2.77 to −1.58). GLP-1 monotherapy also showed decreases in weight (MD −5.22 kg) and BMI (MD −2.00). Waist circumference was significantly reduced by GLP-1 alone (MD −4.70 cm). None ofthe interventions showed significant effects on HOMA-IR, and results were marked by heterogeneity. Sensitivity analyses confirmed robustness for weight and BMI outcomes.

**Conclusions:**

GLP-1 receptor agonists combined with metformin appear to be the most effective pharmacologic therapies for anthropometric improvement, specifically body weight and BMI reduction, in women with PCOS. These conclusions are limited to anthropometric and metabolic parameters, reproductive, endocrine, and patient-centered outcomes were not evaluated in this analysis and therefore no claims of overall superiority in PCOS management can be made. Further long-term and head-to-head RCTs including a broader range of outcomes are needed.

**Systematic Review Registration:**

https://osf.io/zyhws/, identifier 10.17605/OSF.IO/ZYHWS

## Highlights

- GLP-1 receptor agonists combined with metformin produced the greatest reductions in body weight and BMI among women with PCOS
- GLP-1 monotherapy significantly reduced waist circumference, whereas no treatment demonstrated a statistically significant effect on HOMA-IR
- These findings support GLP-1–based therapies as the most effective pharmacologic strategy for anthropometric improvement in women with metabolically active PCOS.

## Background

Polycystic Ovary Syndrome (PCOS) is a prevalent endocrine disorder affecting up to 15% of women of reproductive age (1). While its exact cause is unknown, it is linked to a plethora of metabolic, reproductive, and hormonal disturbances such as menstrual irregularities, insulin resistance, and obesity. Among these disturbances, metabolic dysfunction, especially obesity and insulin resistance significantly contribute to the development of long-term issues such as type 2 diabetes, dyslipidemia, and cardiovascular disease. Current international and US guidelines, including the 2023 International Evidence-Based Guideline for the Assessment and Management of Polycystic Ovary Syndrome, consistently recommend lifestyle modification as the first-line treatment for PCOS across all phenotypes and weight categories (2). Lifestyle interventions, including dietary changes, increased physical activity, and behavioral strategies, are advised for all women with PCOS to improve metabolic health, central adiposity, lipid profile, general health, quality of life, body composition, and weight management (3). However, pharmacologic interventions are often required in addition to lifestyle modifications in order to address the metabolic issues associated with PCOS.

Metformin, a biguanide drug, has long been used as the “go-to” drug to improve insulin resistance and promote weight loss in women with PCOS (4). However, while metformin improves insulin sensitivity and has modest effects on weight, its impact on weight loss and central adiposity is generally limited (5). For instance, a 2014, 12-week long clinical trial employing metformin as a weight loss tool in women diagnosed with PCOS saw an average decrease in weight (kg) by -1.2 ± 0.5 with metformin (from 103.2 ± 6.3 to 102 ± 6.8), while the same study saw a mean difference of -3.8 ± 1.3 with liraglutide, a GLP-1 receptor agonist, (from 108.9 ± 15.1 to 105.1 ± 13.8) (6). The same study also saw a median difference in HOMA-IR score with metformin from 3.8 (3.8) to 2.5 (2.4), while the median difference with liraglutide was from 2.4 (2.3) to 2.1 (2.0). This shows that while metformin can hold clinically significant results regarding weight loss and a change in HOMA-IR, it may not always compare to the difference seen with other interventions, namely GLP-1 receptor agonists (7). Moreover, myoinositol, a naturally-occurring insulin sensitizing agent has recently gained attention as a complementary or even an alternative treatment for PCOS (8). Despite there being various pharmacological interventions used in the treatment of PCOS, there has been limited comparison of their efficacy in head-to-head trials, and the optimal strategy to treat the metabolic issues associated with PCOS remains unclear.

To fill in this gap, we have conducted a comprehensive network meta-analysis comparing the efficacy of GLP-1 receptor agonists, metformin, and myoinositol, alone or in combination, in improving metabolic outcomes among women diagnosed with PCOS. Their comparative effectiveness on weight, BMI, waist circumference, and HOMA-IR was evaluated by utilizing both direct and indirect evidence among randomized clinical trials (RCT’s). The aim of our analysis is to determine the efficacy of relative pharmacologic treatment options for clinical decision making in this metabolically complex condition.

Direct comparisons in the form of head to head trials between GLP-1 receptor agonists, metformin, and myoinositol in the management of metabolic parameters in PCOS are rare, where most RCTs evaluate only one or two interventions in isolation. This scattered evidence makes it extremely challenging for clinicians to dictate which pharmacologic strategy offers superior metabolic benefits. Therefore, a network meta-analysis is required as it offers a methodologically robust solution by integrating both direct and indirect evidence to provide clinical insight into the management of PCOS. This approach facilitates treatment ranking and provides clinicians with a better understanding of a formative strategy for guiding evidence-based decision making in this population.

## Methods

This systematic review and network meta-analysis was conducted in adherence with the Preferred Reporting Items for Systematic Reviews and Meta-Analyses for Network Meta-Analyses (PRISMA-NMA) guidelines (9). The protocol was also prospectively registered on the Open Science Framework (OSF; Registration DOI: 10.17605/OSF.IO/ZYHWS). The study design utilized a frequentist framework using the netmeta R package to synthesize direct and indirect evidence from randomized controlled trials (10).

We included randomized controlled trials (RCTs) enrolling non-menopausal women diagnosed with polycystic ovary syndrome (PCOS). Eligible interventions included GLP-1 receptor agonists (e.g., liraglutide, exenatide, semaglutide), metformin, and myoinositol (with or without FA), either as monotherapy or in combination. Comparators included placebo or other active pharmacologic agents. Studies were required to report at least one of the following outcomes: change in body weight, BMI, waist circumference, or HOMA-IR. Trials were excluded if they were not RCTs, involved pediatric populations, lacked a relevant comparator, or did not report outcome data.

We systematically searched PubMed, Scopus, and Web of Science on February 12, 2025.

Search terms included combinations of “GLP-1 receptor agonists,” “metformin,” “inositol,” “PCOS,” and “randomized controlled trial,” with Boolean operators and MeSH-aligned phrasing adapted to each database. No language or publication status restrictions were applied at the search stage, though non-English full texts were excluded later during full-text review. After deduplication, 849 titles and abstracts were screened by two independent reviewers using Rayyan. Full texts of 80 potentially eligible studies were retrieved, with 64 ultimately excluded for reasons including wrong drug, outcome, or study design. Disagreements during screening were resolved by a third independent reviewer.

Four reviewers independently extracted data using a standardized, piloted form. Extracted variables included sample size, intervention arms, treatment duration, baseline BMI, and reported values for each outcome of interest. For studies with multiple dose arms, only the highest dose was retained for analysis to reflect the maximal therapeutic effect, enhance comparability across trials, and avoid unit-of-analysis errors associated with including multiple arms from the same study. Risk of bias was assessed using the Cochrane Risk of Bias tool across standard domains to assess study quality. (11) We also evaluated overall confidence in network estimates using the CINeMA (Confidence in Network Meta-Analysis) framework (12).

We conducted a frequentist random-effects network meta-analysis using the netmetapackage in R. Mean differences (MDs) with 95% confidence intervals were calculated for all continuous outcomes. Placebo was designated as the reference comparator. Relative treatment rankings were made based on P-scores, which quantify the certainty that one intervention is superior to another on a scale from 0 to 1. A higher P-score indicates a greater probability that the respective treatment yields a more favorable outcome compared with competing interventions, whereas a lower P-score reflects relatively poorer efficacy for that outcome.

Heterogeneity across comparisons was evaluated using the I² statistic, τ², and Cochrane’s Q test (13). Global inconsistency was assessed using design-by-treatment interaction models (14). Transitivity was evaluated by comparing baseline covariates, including BMI and intervention duration, across treatment nodes using ANOVA and boxplot visualizations. Sensitivity analyses included leave-one-out models and the exclusion of studies with high risk of bias to assess the robustness of overall findings.

Cochrane RoB 2 was also used to assess the risk of bias, where no significant risk of bias was found in the random sequence generation or selective reporting for any of the studies used. However, when it came to allocation concealment, blinding of participants/personnel, blinding of outcome assessment, incomplete outcome data, and overall judgement, some studies portrayed a low risk of bias, while others displayed a high risk of bias. The certainty of evidence for each treatment comparison was evaluated using the Confidence in Network Meta-Analysis (CINeMA) framework, which adapts the GRADE approach for network meta-analyses. CINeMA assesses six domains: within-study bias (informed by the Cochrane RoB 2 tool) (11), across-studies bias, indirectness, imprecision, heterogeneity, and incoherence. Each comparison was rated as high, moderate, low, or very low confidence.

The outcomes of interest were metabolic parameters reflecting the efficacy of each intervention in improving metabolic profiles among women with polycystic ovary syndrome. Body weight (kg) was assessed to evaluate absolute reductions in mass, independent of height. Body mass index (BMI) was measured as weight in kilograms divided by the square of height in meters (kg/m²) and served as an indicator of overall adiposity. Waist circumference (cm) represented central adiposity and was measured at the midpoint between the lowest rib and the iliac crest using a standardized tape measure. Insulin resistance was estimated using the Homeostasis Model Assessment of Insulin Resistance (HOMA-IR), calculated as fasting insulin (µU/mL) × fasting glucose (mmol/L)/22.5. Lower values across all outcomes indicated more favorable metabolic improvement.

## Results

**Figure 1:**
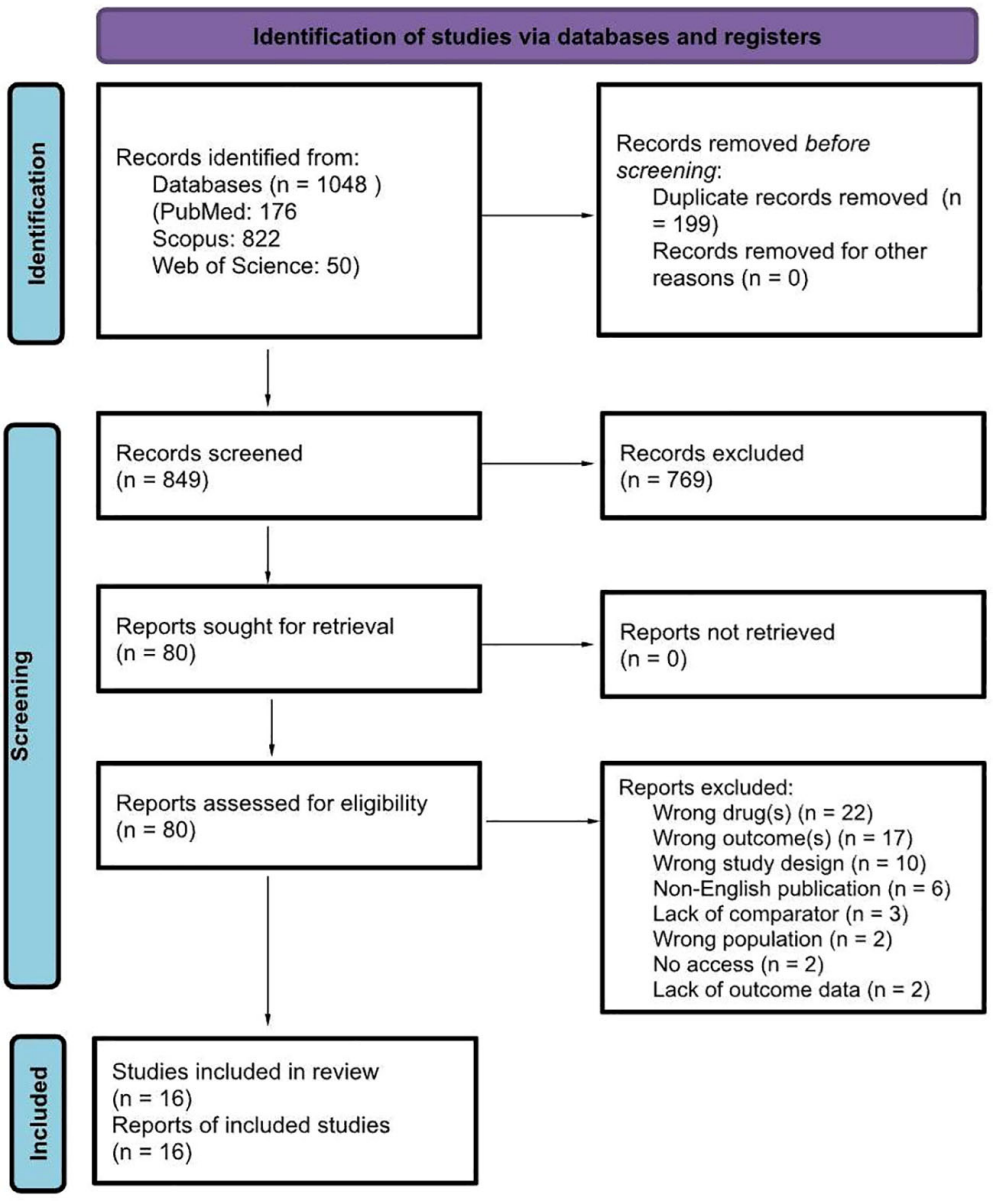
PRISMA flow diagram showing identification, screening, eligibility assessment, and inclusion of randomized controlled trials in the network meta-analysis.

A total of 849 records were identified through database searching. After removing duplicates and screening titles and abstracts, 80 full-text articles were assessed for eligibility. Of these, 16 randomized controlled trials were included in the final analysis (15–30). The characteristics of the included studies are summarized in **Table 1**. These studies collectively enrolled women with PCOS and compared GLP-1 receptor agonists (e.g., liraglutide, semaglutide, exenatide), metformin, myoinositol with or without FA, and placebo, either as monotherapy or in combination. Treatment durations ranged from 8 to 32 weeks, and all participants were premenopausal and most studies ensured patients met the Rotterdam criteria for PCOS diagnosis. This is seen in the PRISMA checklist which we followed for inclusion or exclusion of the studies we eventually used (**Figure 1**).

**Table 1:** Study characteristics table, **Table 1** in supplementary file.

Study ID	Trial phase	Year	Code	Population included	Population excluded	Number of participants	Intervention	Comparator	Duration (weeks)
Wen,2 023 (15)	Pha se 3	202 3	ChiC TR20 0003 3741	BMI ≥ 24kg/m2 with PCOS, age 18-40.	DM, CKD, pregnancy.	64 (60 complete d)	beinaglutide (0.1 mg TID increasing to 0.2 mg TID) + metformin (850 mg BID)	metfor min (850 mg BID)	12 weeks
Xing, 2022 (16)	Pha se 3	202 2	NCT 0496 9627	Diagnosed via Rotterdam Criteria, BMI ≥ 24 kg/m2, age 18-40, no medication affecting insulin sensitivity or ovarian function within first three months of trial, use barrier contraception.	GLP-1 RAs or MET allergy; CVD, abnormal LFTs, renal insufficiency, thyroid dysfunction; history of cancer, active infection, weekly alcohol intake > 100 g, pregnancy and/or breastfeeding, 17-hydroxyprogesterone level > 2 ng/mL	60 (52 complete d)	metformin (1000 mg BID)	metfor min (1000 mg BID) + Liraglut ide (1.2 mg QD)	12 weeks
Elkind-Hirsch, 2022 (17)	Pha se 3	2022	NCT 03480022	Irregular menstrual cycles and biochemical hyperandrogenism, and BMI >30 kg/m²	DM, smoking within 6 months, pregnancy/lactation, systemic disease, uncontrolled hypertension, acute pancreatitis, injectable hormonal contraceptive within 6 months, OCPs, steroid hormones, drugs affecting GI motility/carbohydrate metabolism, and/or anti-obesity drugs within 3 months.	82 (67 complete d)	liraglutide 3 mg QD	Placebo	32 weeks
Ma,20 21 (18)	Pha se 3	2021	NCT 04029272	Diagnosed according to Rotterdam criteria, 18 to 40 years old and overweight/obese (BMI ≥25 kg/m2).	DM, history of cancer, personal/family history of MEN type 2, CVD, kidney, or liver diseases, and use of drugs affecting reproductive/metabolic functions within 3 months.	50 (40 complete d)	metformin (500 mg TID) + exenatide (2 mg QW)	metfor min (500 mg TID)	12 weeks
Soldat-Stank ović,2 022 (19)	Pha se 3	2022	ISRC TN1 3199 265	Diagnosed according to the Rotterdam criteria, age 18-40 (33 normal- weight, 33 overweight/obese)	Thyroid dysfunction, hyperprol-actinemia, Cushing syndrome, NCAH, androgen-secreting tumors, DM, hepatic, renal and CVS disorders, history of alcohol/drug abuse, breast/uterine cancer history	66 (60 complete d)	metformin (500 mg TID)	myo-in ositol (2 g BID) + FA (200 µg BID)	24 weeks
Tao,2 021 (20)	Pha se 3	2021	NCT 03352869	Diagnosed by Rotterdam criteria, age 18-45, BMI ≥25 kg/m2, no hypoglycemic drugs within 3 months or dietary/behavioral intervention for 3 months but met OGTT criteria for prediabetes.	GLP-1RA or MET allergy, severe liver function test abnormality, renal dysfunction, hypertension, active infection, secondary diabetes, and active alcohol misuse, pregnancy, or breast feeding.	183 (150 in complete d)	exenatide (starting at 10 µg for the first 4 weeks, then increasing to 20μg daily)	metfor min (1000 mg BID)	12 weeks
Zheng,2019 (21)	Pha se 3	2019	PMI D: 3091 8134	Diagnosed by the Rotterdam criteria	NR	182 (63 complete d)	exenatide (10 μg BID)	metfor min (1000 mg BID)	12 weeks
Shork pour,2 019 (22)	Pha se 3	2019	IRCT 2017082733941N10	Diagnosed by the Rotterdam criteria, age 18–40	Pregnancy, CAH, androgen-secreting tumors, hyperprolactinemia, thyroid dysfunction, and DM.	60 (53 complete d)	myo-inositol (2 g BID) + FA (200 µg BID)	metfor min (500mg TID)	12 weeks
Frøssi ng, 2018 (23)	Pha se 3	2018	NCT 02073929	PCOS (Rotterdam criteria), BMI > 25 kg/m2 and/or presence of IR.	DM, use of hormonal contra-ceptives 6 weeks before or insulin sensitizers 3 months before trial.	72 (65 complete d)	liraglutide 1.8 mg QD	Placebo	26 weeks
Jenste rle_a, 2017 24	Pha se 3	2017	NCT 0290 9933	Diagnosed by Rotterdam criteria, hyperandrogenemia, menses abnormalities, age 18- premenopause, BMI ≥ 30	Patients with history of carcinoma, significant CVS, kidney or hepatic disease and use of medications affecting reproductive or metabolic functions prior to study entry.	30 (28 complete d)	liraglutide (1.2 mg QD) + metformin (1000 mg BID)	liraglut ide 3 mg QD	12 weeks
Zahra, 2016 (25)	Pha se 3	2016	NR	Diagnosed by Rotterdam criteria	NR	60 (40 complete d)	metformin (500 mg TID)	Placebo	
Nguye n,2023 (26)	Pha se 3	2023	NR	Age 18–40 years with diagnosed by Rotterdam criteria:	CAH, androgen producing tumors, Cushing’s, history of ovarian surgery, tumors, endometriosis, or failure, obstruction of fallopian tubes, and OAT.	171 (132 complete d)	myo-inositol (500 mg QID)	metfor min (850 mg BID)	12 weeks
Ravn, 2022 (27)	Pha se 3	2022	NR	Diagnosed by the Rotterdam criteria and age 18–50	Abnormal prolactin, TSH, or 17-hydroxy-progesterone values, postmenopausal values of FSH (>25 IE/L), and type 1 or 2 DM.	45 (28 complete d)	myo-inositol (2 mg BID) + FA (200 µg BID)	metfor min (500 mg BID)	24 weeks
Zheng,2017 (28)	Pha se 3	2017	Chi C TR-I IR-1 600 8084	18–40 years diagnosed by Rotterdam criteria, BMI >24 kg/m2 = overweight, and BMI >28 kg/m2 = obese.	Other causes of hyperandrogenemia and abnormal ovulation such as CAH, Cushing’s syndrome, hyperprolactinemia and testosterone-secreting tumors.	82 (63 complete d)	exenatide (10 µg BID)	metfor min (1000 mg BID)	12 weeks
Jenste rle_b, 2016 (29)	Pha se 3	2016	NCT 02483299	Hyperandrogenemia, menstrual abnormalities, PCOS morphology, >18 years and premenopausal, and BMI ≥30	History of carcinoma, cardiovascular, kidney or hepatic disease, medications known to affect reproductive/metabolic functions prior to study entry. Certain subjects did, however, use oral contraceptives >6 months prior to being recruited.	44 (43 complete d)	liraglutide 1.2 mg QD	metfor min (1000 mg BID) + Liraglut ide (1.2 mg QD)	12 weeks
Jenste rle_c, 2015 (30)	Pha se 3	2015	NCT 01899430	18 years to menopause and BMI ≥30	Type 1 or 2 DM, carcinoma history, MEN 2 personal/family history, significant CVS, kidney or hepatic disease, and medications affecting reproductive/metabolic functions 90 days prior to study entry.	32 (17 in Liragluti de group, 15 in Metform in group)	liraglutide (1.2 mg QD)	metfor min (1000 mg BID)	12 weeks

**Figure 2:**
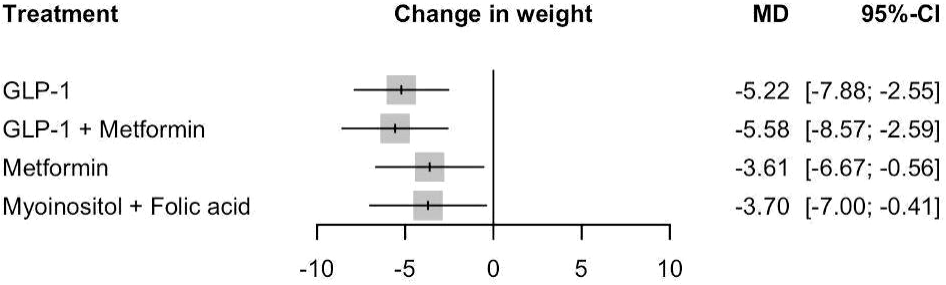
Forest plot for weight change showing network meta-analysis estimates versus placebo.

**Figure 3:**
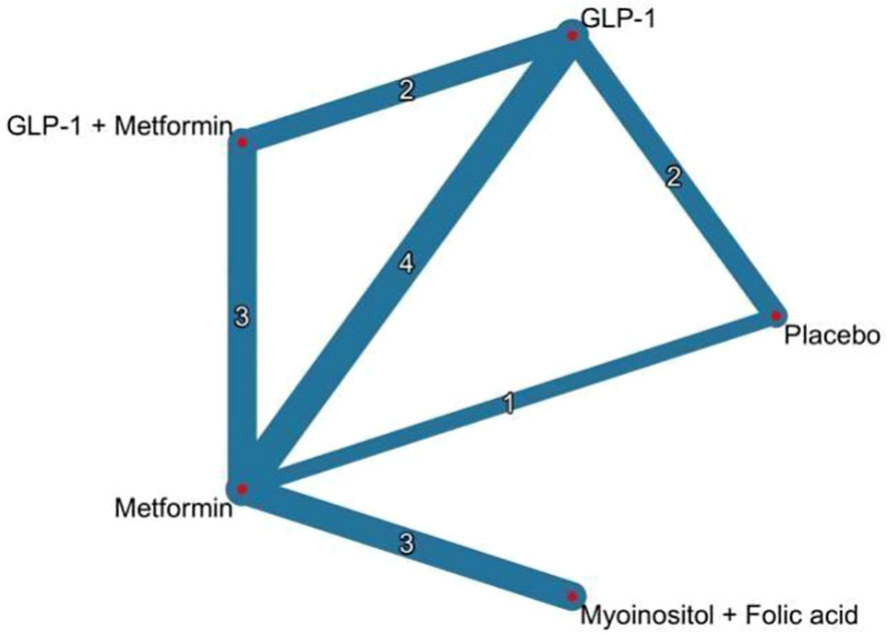
Network plot showing the geometry of treatment comparisons included in the analysis. Nodes represent interventions; numbers on connecting lines indicate the number of trials directly comparing each pair.

Weight Change GLP-1 + metformin showed the highest efficacy, resulting in a mean difference (MD) of −5.58 kg (95% CI: −8.57 to −2.59, p = 0.0003) compared to placebo (**Figure 2**). GLP-1 alone also produced a substantial reduction (MD: −5.22 kg, 95% CI: −7.88 to −2.55, p = 0.0001). Myoinositol + FA and metformin (MD: −3.70 kg, 95% CI: −6.99 to −0.41, p = 0.0276) also outperformed placebo. Based on P-scores, GLP-1 + metformin ranked highest (P-score: 0.919), followed by GLP-1 (0.804), then myoinositol + FA (0.407), and finally metformin (0.363). Heterogeneity was low (I² = 9.7%, τ² = 0.315), with no significant global inconsistency. A local inconsistency was observed in the GLP-1 vs GLP-1 + metformin comparison (p = 0.0007). Meta-regression suggested that longer treatment duration was associated with greater weight reduction (p = 0.06), though this did not reach statistical significance. Transitivity was satisfied, with no baseline imbalance in BMI or duration across treatment nodes (**Figure 3**). Sensitivity analyses confirmed robustness of results. Leave-one-out analyses demonstrated stable results across outcomes. For weight, GLP-1 and GLP-1 plus metformin consistently showed significant reductions compared with placebo, while metformin alone also retained a modest but significant effect; myoinositol plus FA trended toward benefit but did not reach statistical significance. Funnel plot asymmetry assessments were underpowered, so publication bias cannot be excluded.

### Change in BMI

**Figure 4:**
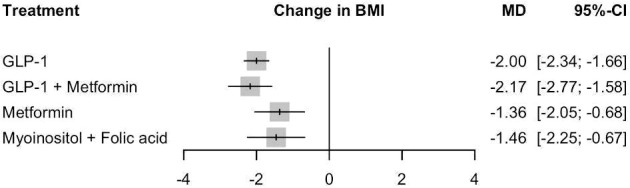
Forest plot for BMI change showing network meta-analysis estimates versus placebo.

GLP-1 + metformin showed the largest reduction in BMI (MD: −2.17, 95% CI: −2.77 to −1.58, p < 0.0001), closely followed by GLP-1 alone (MD: −2.00, 95% CI: −2.34 to −1.66, p < 0.0001) (**Figure 4**). Myoinositol + FA (MD: -1.46) and metformin (MD: -1.36) also significantly outperformed placebo. Treatment rankings mirrored weight loss: GLP-1 + metformin ranked highest (P-score: 0.937), followed by GLP-1 (0.789).

Heterogeneity was low (I² = 7.4%), and sensitivity analyses confirmed the stability of results. Meta-regression showed a significant effect of intervention duration (p = 0.0027), explaining over half of between-study variance (R² = 56.2%).

Funnel plot asymmetry assessments were underpowered, so publication bias cannot be excluded.

### Change in waist circumference

**Figure 5:**
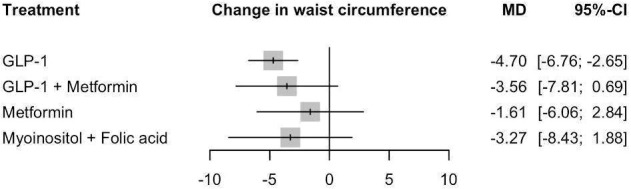
Forest plot for waist circumference change showing network meta-analysis estimates versus placebo.

Only GLP-1 monotherapy significantly reduced waist circumference compared to placebo (MD: −4.70 cm, 95% CI: −6.76 to −2.65, p < 0.0001). Other interventions showed numerically favorable but non-significant reductions. GLP-1 ranked highest (P-score: 0.847), followed by GLP-1 + metformin (0.692) and myoinositol + FA (0.624) (**Figure 5**).

Moderate heterogeneity was observed (I² = 50.5%), with a significant inconsistency between GLP-1 + metformin and metformin (p = 0.0033). Meta-regression found no effect of BMI or duration (p = 0.909), and R² = 0%. Transitivity was satisfied. Sensitivity analyses using leave-one-out models and exclusion of high-risk-of-bias studies confirmed the robustness of the findings, as the direction and magnitude of effects remained unchanged across iterations.

In waist circumference, the strongest effect was observed with GLP-1 plus metformin, whereas metformin and myoinositol comparisons remained nonsignificant. Collectively, these findings indicate that the primary conclusions for weight and waist circumference are robust, while outcomes related to insulin resistance appear more sensitive to study exclusion.

Funnel plot asymmetry assessments were underpowered, so publication bias cannot be excluded.

### Change in HOMA-IR

**Figure 6:**
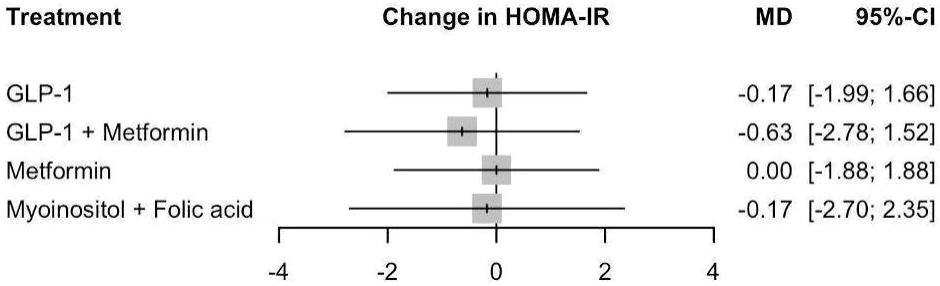
Forest plot for HOMA-IR change showing network meta-analysis estimates versus placebo.

None of the included treatments showed a statistically significant effect on HOMA-IR compared to placebo. GLP-1 + metformin showed the largest reduction (most favorable) MD (−0.63; 95% CI: −2.78 to 1.52, p = 0.565), followed by myoinositol + FA (−0.17; 95% CI: −2.70 to 2.35, p = 0.892). P-scores indicated GLP-1 + metformin ranked highest (0.742), but confidence intervals crossed the null for all interventions (**Figure 6**).

Heterogeneity was very high (I² = 84.9%, τ² = 1.30), and multiple nodes exhibited significant inconsistency, including GLP-1 vs metformin (p < 0.0001). Transitivity was preserved across arms. Sensitivity analyses, including leave-one-out models and exclusion of high-risk-of-bias studies, demonstrated that the direction of effect consistently favored GLP-1 + metformin; however, the magnitude of the effect fluctuated substantially, and confidence intervals remained wide, confirming that imprecision rather than directionality accounted for the non-significant results. These findings collectively reduce confidence in the robustness of HOMA-IR conclusions.

Funnel plot asymmetry assessments were underpowered, so publication bias cannot be excluded.

## Discussion

This network meta-analysis demonstrates that GLP-1 receptor agonists, especially when combined with metformin, are the most effective pharmacologic options assessed in this analysis for promoting weight loss and improving body mass index in women with PCOS. It should be noted that this conclusion is specific to anthropometric outcomes, as reproductive and endocrine parameters were not evaluated.

GLP- 1 + metformin yielded the largest mean weight reduction (−5.58 kg), followed by GLP- 1 monotherapy (−5.22 kg), with both significantly outperforming metformin and myoinositol + FA. Similarly, both agents showed statistically significant effects on BMI. Waist circumference was significantly reduced by GLP- 1 alone, while no statistically significant improvements were observed for HOMA-IR. Treatment rankings based on P-scores were consistent across outcomes, with GLP- 1 + metformin or GLP- 1 ranking the highest. These findings held across multiple sensitivity analyses and were supported by balanced baseline variables and low heterogeneity for most outcomes.

Metformin, myoinositol, and GLP-1 receptor agonists act through different yet complementary mechanisms in PCOS. Metformin enhances insulin sensitivity by suppressing hepatic gluconeogenesis and improving peripheral glucose uptake, reducing hyperinsulinemia and downstream ovarian androgen production (31, 39). myoinositol, an insulin-sensitizing agent involved in the inositol-phosphoglycan signaling pathway, improves intracellular insulin signaling and restores follicular microenvironment homeostasis, favorably impacting ovulatory and metabolic function (32). GLP-1 receptor agonists reduce caloric intake by delaying gastric emptying, suppressing appetite, and promoting satiety, while also enhancing glucose dependent insulin secretion and lowering glucagon levels (33). These mechanisms explain our findings: the combination of GLP-1 receptor agonists with metformin yielded the greatest improvements in weight loss, BMI reduction, and waist circumference, reflecting the synergistic effects of central appetite suppression with peripheral insulin sensitization. On the other hand, metformin alone achieved modest but consistent metabolic benefits, while myoinositol, though less potent in anthropometric outcomes, supported improvements in insulin resistance and reproductive parameters. The pharmacological complementarity of GLP-1 and metformin provides a strong argument for the superior outcomes observed in our network meta-analysis.

The extent of weight loss observed with GLP-1 receptor agonists, especially in combination with metformin, is clinically relevant in the context of PCOS, a condition in which insulin resistance, central obesity, and metabolic dysfunction contribute to eventual infertility and long-term cardiometabolic risk (34, 35). A reduction of over 5 kg in weight and over 2 points in BMI may enhance ovulatory function, reduce androgen levels, and improve quality of life (36, 37). These findings support the use of GLP-1 receptor agonists as a metabolically targeted strategy for anthropometric improvement in women with PCOS. Whether these benefits extend to reproductive or endocrine outcomes requires evaluation in future trials. Given that both GLP-1 agents and metformin are already used clinically, our results will help guide treatment sequencing and combination strategies, particularly for overweight or insulin-resistant patients with PCOS.

Given the heterogeneity of PCOS phenotypes, a personalized, combination-based therapeutic approach may optimize outcomes beyond a uniform treatment model. Patients with predominant metabolic or obese phenotypes—characterized by insulin resistance and central adiposity—may derive the greatest benefit from GLP-1 receptor agonists in combination with metformin, leveraging the synergistic effects of appetite suppression and enhanced insulin sensitivity. Conversely, those with milder metabolic profiles or primarily reproductive dysfunction may respond adequately to myoinositol-based therapy, which offers insulin-sensitizing and ovulatory benefits with minimal adverse effects. Furthermore, gradual sequencing—initiating metformin or myoinositol in treatment-naïve patients, followed by glucagon-like peptide-1 receptor agonist (GLP-1 RA) introduction if weight loss or metabolic targets remain unmet—could personalize therapy while maintaining tolerability and cost-effectiveness. Integrating baseline characteristics such as BMI, insulin resistance index, and reproductive goals into treatment selection frameworks may therefore enable more tailored, patient-centered management of PCOS.

Our findings align with and further extend prior meta-analyses that have shown benefits of GLP-1 receptor agonists in PCOS (38). However, previous analyses often lacked direct comparisons among all available pharmacologic treatments, particularly myoinositol or combination therapies. By incorporating indirect evidence from various RCT’s, our network meta-analysis confirms and strengthens the position of GLP-1 receptor agonists as leading agents specifically for weight and BMI reduction in women with PCOS, within the scope of anthropometric outcomes assessed. Notably, our results differ from earlier reviews that attributed greater effects to myoinositol; when placed alongside GLP-1 agents, its relative efficacy was consistently lower. This analysis clarifies where each intervention stands within the broader pharmacological options and provides essential comparative insights that single agent trials simply cannot offer.

This study has several strengths. We used a pre-registered protocol and followed PRISMA-NMA guidelines to ensure transparency and methodological diligence. Our comprehensive database search, conducted without language restrictions, maximized trial identification. All included studies were randomized controlled trials, increasing the reliability of pooled estimates. We additionally employed a frequentist network meta-analysis framework with sensitivity analyses, moderator testing, and consistency checks, all of which confirmed the robustness of our results. Transitivity assumptions were assessed statistically and met. Finally, the CINeMA framework was additionally employed to evaluate the certainty of evidence and a structured transitivity assessment using baseline covariate balance.

Despite its many strengths, this analysis also has several limitations. Some comparisons, particularly those involving HOMA-IR, showed high heterogeneity and inconsistency, limiting interpretability. The network was anchored primarily on placebo arms, and relatively few trials directly compared active agents to each other. Additionally, while transitivity was preserved statistically, variation in study durations and dosing strategies may still introduce clinical heterogeneity. Although most included trials were rated as low or moderate risk of bias, limited reporting on allocation concealment in some studies reduces overall certainty. Additionally, most included studies did not consistently report or stratify outcomes based on lifestyle factors such as physical activity or dietary interventions, which may act as important confounders influencing metabolic outcomes.

Our findings highlight the need for head to head RCTs comparing GLP-1 receptor agonists, metformin, and myoinositol, especially in combination treatments, to establish optimal treatment strategies. Long-term trials are also recommended to assess durability of metabolic improvement, effects on fertility outcomes, and safety. Subgroup analyses in women with differing phenotypes of PCOS (e.g., lean PCOS, adolescents, those with metabolic syndrome) could also further refine treatment selection. Future meta-analyses would benefit from more standardized outcome reporting and direct comparisons of GLP-1 agonists themselves (e.g., semaglutide vs. liraglutide) to enhance precision in clinical decision making.

## Conclusion

This network meta-analysis evaluated the comparative effectiveness of GLP-1 receptor agonists, metformin, and myoinositol in improving anthropometric and metabolic outcomes among women with PCOS. GLP-1 receptor agonists, especially in combination with metformin, emerged as the most effective interventions for reducing body weight and BMI, with GLP-1 alone also significantly reducing waist circumference. While no intervention showed a statistically significant effect on HOMA-IR, findings across the other anthropometric outcomes were robust, supported by low heterogeneity, consistent treatment rankings, and balanced study populations. These results offer important evidence to inform pharmacologic decision making regarding weight and metabolic management in PCOS, particularly for patients with obesity or metabolic dysfunction. Importantly, the scope of this analysis is limited to anthropometric and metabolic parameters. Reproductive outcomes, endocrine endpoints such as androgen levels and menstrual regularity, and patient-centered outcomes were not assessed; therefore, conclusions regarding overall superiority in PCOS management cannot be drawn. Future head-to-head trials with longer follow-up, standardized outcome reporting, and inclusion of reproductive and endocrine endpoints are needed to validate and extend these findings.

## Data Availability

The original contributions presented in the study are included in the article/**Supplementary Material**. Further inquiries can be directed to the corresponding author.
