# Correction: Comparative analysis of glucagon-like peptide-1 receptor agonists, metformin, and inositol in improving anthropometric and metabolic outcomes in women with polycystic ovary syndrome: a network meta-analysis

**DOI:** 10.3389/fendo.2026.1932302

**Published:** 2026-07-20

**Authors:** Asad Omarion, Yaman Ayasa, Zainab Omarion, Batoul Jayouse, Hazem Ayesh

**Affiliations:** 1 Faculty of Medicine, Al-Quds University, Jerusalem, Palestine; 2 Deaconess Clinic Endocrinology, Deaconess Health System, Evansville, IN, United States

**Keywords:** GLP-1 receptor agonists, insulin resistance, metformin, myoinositol, network meta-analysis, obesity, polycystic ovary syndrome

The figure captions were published in the wrong order relative to their corresponding images. Specifically, the captions for **Figure 3**, **4** and **5** were each shifted one position ahead of the image to which they belong. This has been corrected so that each caption now matches its corresponding figure, as follows:

**Figure 3:** “Network plot showing the geometry of treatment comparisons included in the analysis. Nodes represent interventions; numbers on connecting lines indicate the number of trials directly comparing each pair.”

**Figure 4:** “Forest plot for BMI change showing network meta-analysis estimates versus placebo.”

**Figure 5:** “Forest plot for waist circumference change showing network meta-analysis estimates versus placebo.”

**Figure 6:** “Forest plot for HOMA-IR change showing network meta-analysis estimates versus placebo.”

The original version of this article has been updated.

