# Supplementary material for: Comparative analysis of glucagon-like peptide-1 receptor agonists, metformin, and inositol in improving anthropometric and metabolic outcomes in women with polycystic ovary syndrome: a network meta-analysis
Source: Front Endocrinol (Lausanne). 2026 Jul 3;17:1833904. doi: 10.3389/fendo.2026.1833904 (PMC13375489; doi:10.3389/fendo.2026.1833904)
Supplement: Supplementary file 1 [file DataSheet1.pdf]

1. Search strategy

Databases Searched:

1. Pub Med Search Dates

- Start Date: [February 12]

- End Date: [February 12]

PubMed Search Query Syntax:

(("GLP-1 Receptor Agonists" OR "GLP-1 agonists" OR "glucagon-like peptide-1 receptor agonist" OR "liraglutide" OR "dulaglutide" OR "exenatide" OR "semaglutide" OR "albiglutide" OR "GLP1RA") OR ("Metformin" OR "biguanides" OR "metformin hydrochloride")) OR (("Inositol" OR "myo-inositol" OR "D-chiro-inositol" OR "inositol supplementation")) AND ("randomized controlled trial" OR "clinical trial" OR "meta-analysis") AND ("Polycystic Ovary Syndrome" OR "PCOS" OR "polycystic ovary syndrome" OR "Stein-Leventhal Syndrome")

2. Sco pus Search

Dates

- Start Date: [February 12]

- End Date: [February 12]

Scopus Search Query Syntax:

(("GLP-1 Receptor Agonists" OR "GLP-1 agonists" OR "glucagon-like peptide-1 receptor agonist" OR "liraglutide" OR "dulaglutide" OR "exenatide" OR "semaglutide" OR "albiglutide" OR "GLP1RA") OR ("Metformin" OR "biguanides" OR "metformin hydrochloride")) OR (("Inositol" OR "myo-inositol" OR "D-chiro-inositol" OR "inositol supplementation")) AND ("randomized controlled trial" OR "clinical trial" OR "meta-analysis") AND ("Polycystic Ovary Syndrome" OR "PCOS" OR "polycystic ovary syndrome" OR "Stein-Leventhal Syndrome")

3. Web of

Science Search

Dates

- Start Date: [February 12]

- End Date: [February 12]

Web of Science Search Query Syntax:

(((((((((((ALL=(GLP-1 Receptor Agonists)) OR ALL=(GLP-1 Agonists)) OR ALL=(Metformin)) OR ALL=(Inositol)) OR ALL=(myo-inositol)) OR ALL=(D-chiro-inositol)) AND ALL=(randomized controlled trial)) OR ALL=(clinical trial)) OR
ALL=(meta-analysis)) OR ALL=(systematic review)) OR ALL=(network meta-analysis)) AND ALL=(Polycystic Ovary Syndrome)) OR ALL=(PCOS)

Number of Records Retrieved:

- PubMed: [176]

- Scopus: [822]

- Web of Science: [50]

Total Records Identified: [1048]

2. Network Geometry

Summary Weight loss

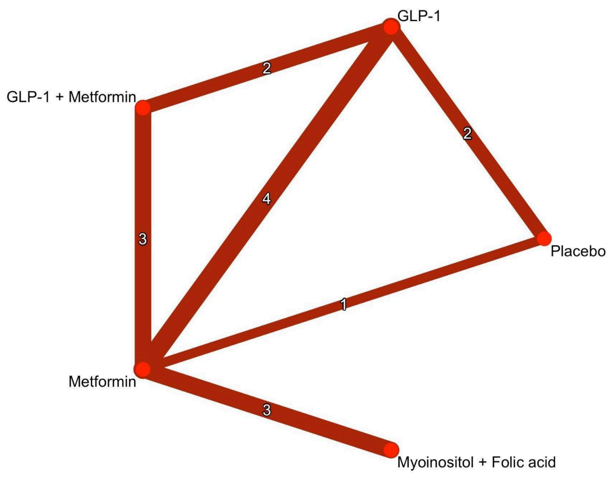

BMI Change

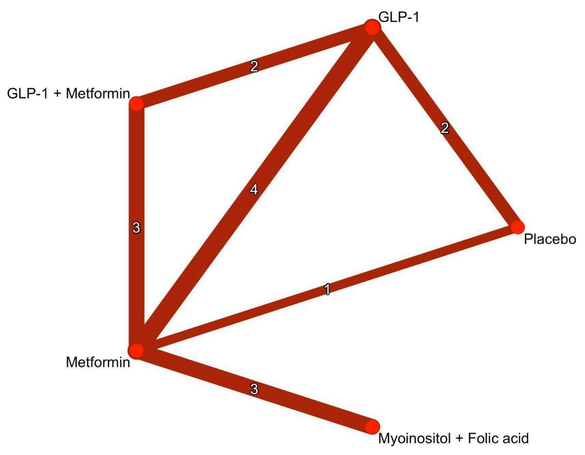

Waist Circumference Change

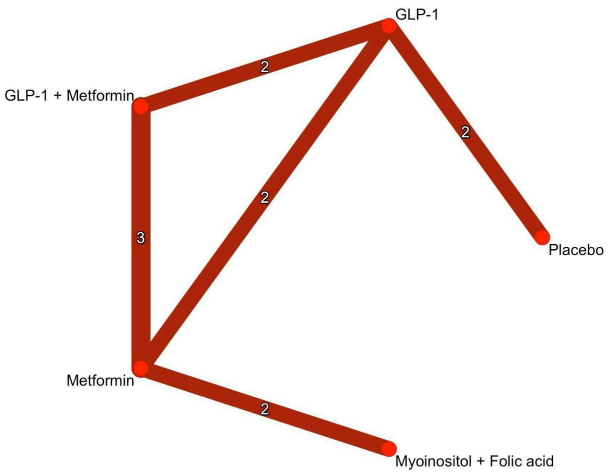

HOMA-IR Change

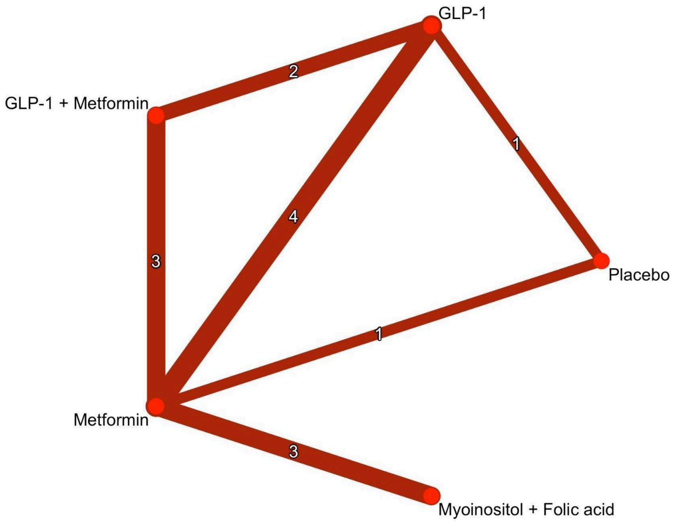

3. Study Characteristics Table

Study ID	Trial Phase	Year	NCT Code	Population Included	Population Excluded	Number of Participants	Intervention	Comparator	Duration (weeks)
Wen, 2023	Phase 3	2023	ChiCTR2000033741	Overweight and obese women (BMI≥24kg/m2), aged 18-40 years who meet the diagnostic criteria of PCOS and promise to continuously utilize barrier contraception while undergoing treatment. The main inclusion requirement also included no therapy that impairs ovarian or insulin sensitivity throughout the first three months of the study.	1) Combined with diabetes mellitus, pituitary tumors, thyroid dysfunction, adrenal tumors, and other endocrinology diseases;2) abnormal liver function and renal insufficiency (alanine transaminase(ALT)levels 2.5 times higher than the upper limit of the normal range, estimated glomerular filtration rate(eGFR) <60mL/min/1.73m2);3)clinically serious diseases of the cardiovascular and hematopoietic system, etc;4)menstrual abnormalities caused by congenital deficiencies or organic lesions such as congenital malformations of reproductive organs and gonadal hypoplasia;5)pregn	64 (60 completed)	Beinaglutide (0.1 mg TID increasing to 0.2 mg TID) + Metformin (850 mg BID)	Metformin (850 mg BID)	12 weeks

					ancy or lactation;6)had a previous episode of acute pancreatitis;7)allergy to GLP-1RAs or metformin;8) did not take the medication as prescribed, or could not judge the therapeutic effect, or could not cooperate with the follow-up;9)usage of injectable or oral hormonal contraceptives within 6 months, other steroid hormones, drugs that affect endocrine indicators and insulin sensitivity, and/or anti-obesity medications before 3 months of study enrollment.				
Xing, 2022	Phase 3	2022	NCT04969627	(1) Meet the Rotterdam diagnostic criteria of PCOS phenotype B with hyperandrogenism and ovulatory dysfunction (15); (2) body mass index (BMI) ≥ 24 kg/m2; (3) age	(1) Allergy to GLP-1 RAs or MET; (2) severe cardiovascular disease; (3) abnormal liver function test results (alanine transaminase (ALT) levels 2.5 times higher than the upper limit of the normal range); (4) renal insufficiency	60 (52 completed)	Metformin (1000 mg BID)	Metformin (1000 mg BID) + Liraglutide (1.2 mg QD)	12 weeks

				between 18 and 40 years; (4) no medication that affects insulin sensitivity or ovarian function within the first three months of the trial; (5) use barrier contraception.	(estimated glomerular filtration rate, eGFR < 60 mL/min/1.73 m2); (5) thyroid dysfunction; (6) history of cancer; (7) active infection; (8) weekly alcohol intake > 100 g; (9) pregnancy and breastfeeding; (10) 17-hydroxyprogesterone level > 2 ng/mL (to exclude women with hyperandrogenemia due to atypical 21-hydroxylase deficiency).				
Elkind-Hirsch,2022	Phase 3	2022	NCT034800222	Eligible subjects were required to have irregular periods (cycle length outside 21–35 days or <8 cycles per year) together with biochemical hyperandro-genism (total testosterone [TT] >50 ng/dL, or free androgen index [FAI] >3.87) and exclusion of known disorders for bleeding irregularities and androgen excess (18).	Exclusion criteria included diabetes diagnosis, smoking within 6 months, pregnancy or lactation, clinically significant systemic disease, uncontrolled hypertension, acute pancrea- titis, injectable hormonal contraceptive use within 6 months, use of oral contraceptives, other steroid hormones, drugs that affect gastrointestinal motility or carbohydrate metabolism, and/or	82 (67 completed)	Liraglutid e 3 mg QD	Placebo	32 weeks

				The main in-clusion criteria also included BMI >30 kg/m2 and agreement to use effective contraception consistently during the therapy.	anti-obesity drugs within 3 months before study entry.				
Ma,2021	Phase 3	2021	NCT04029272	All eligible women with PCOS were recruited from the outpatients from Department of Obstetrics and Gynecology, Peking Union Medical College Hospital, and diagnosed according to the Rotterdam criteria. All subjects were 18 to 40 years old and overweight/obese (body mass index [BMI] ≥25 kg/m2).	The exclusion criteria included patients with diabetes; history of cancer; personal or family history of multiple endocrine neoplasia type 2; severe cardiovascular, kidney, or liver diseases; and use of statins or other drugs known or suspected to affect reproductive or metabolic functions within 3 months before entering the study.	50 (40 completed)	Metformi n (500 mg TID) + Exenatid e (2 mg QW)	Metformin (500 mg TID)	12 weeks
Soldat-Stanković, 2022	Phase 3	2022	ISRCTN13199265	Women with PCOS diagnosed according to the Rotterdam criteria, ages between 18 and 40 years old (33 normal-weight, 33	Women suffering from thyroid dysfunction, hyperprol-actinemia, Cushing syndrome, non-classical congenital adre-nal hyperplasia (NCAH), and	66 (60 completed)	Metformi n (500 mg TID)	Myo-inositol (2 g BID) + Folic acid (200 µg BID)	24 weeks

				overweight/obes e)	androgen-secreting tumors were excluded. Women suffering from diabetes, hepatic, renal and cardiovascular disorders, or having a history of alcohol or drug abuse or medical history of breast or uterine cancer were excluded from the study.				
Tao,2 021	Phase 3	2021	NCT03 35286 9	(1) Patients diagnosed with PCOS according to the Rotterdam 2003 criteria [31]; (2) and also met prediabetes criteria. Prediabetes is defined by the presence of IFG and/or IGT and/or A1C 5.7% to 6.4% (39-47 mmol/mol) [4]. IFG is defined as FBG levels between 5.6 and 6.9 mmol/L (100 and 125 mg/dL) and IGT as PPG levels for the 75-g OGTT between 7.8 and 11.0 mmol/L (140	Exclusion criteria were as follows: subjects with self-reported allergy to either glucagon-like peptide 1 receptor agonists (GLP-1RA) or MET; subjects with severe liver func-tion test abnormality (defined as alanine aminotransferase [ALT] 2.5 times or higher than the upper limit of normal range), or renal dysfunction (serum creatinine >132 μmol/L, and/or estimated glomerular filtration rate <60 mL/ min/1.73 m2), hypertension (>160/100 mmHg), active in- fection,	183 (150 in completed)	Exenatid e (starting at 10 μg for the first 4 weeks, then increasi n g to 20μg daily)	Metfor min (1000 mg BID)	12 weeks

				and 199 mg/ dL) [4]; (3) premenopausal patients aged between 18 and 45 years; (4) with a body mass index (BMI) ≥25 kg/m2 as defined by the World Health Organization-Western Pacific Region [32]; (5) patients with their first onset of PCOS that have not received any hypoglycemic drugs in 3 months prior to this trial; or have been treated with dietary and behavioral intervention for 3 months but still met OGTT criteria for prediabetes.	secondary diabetes, and subjects with active al- cohol misuse, pregnancy, or breast feeding.				
Zheng,2019	Phase 3	2019	PMID: 30918134	Women with PCOS diagnosed based on the Rotterdam criteria	NR	182 (63 completed)	Exenatide (10 µg BID)	Metformin (1000 mg BID)	12 weeks
Shorkpour, 2019	Phase 3	2019	IRCT201708273394 1N10	Women with PCOS diagnosed by Rotterdam	Pregnancy, adrenal hyperplasia, androgen-secreting tumors,	60 (53 completed)	Myo-inositol (2 g BID) + Folic	Metformin (500mg TID)	12 weeks

				criteria, aged 18–40 years	hyperprolactinemia, thyroid dysfunction, and diabetes at enrollment.		acid (200 µg BID)		
Frøssing,2018	Phase 3	2018	NCT020739229	PCOS (Rotterdam criteria), BMI > 25 kg/m2 and/or presence of IR.	Diabetes, use of hormonal contra-ceptives 6 weeks before randomization and insulin sensitizers 3 months before randomization.	72 (65 completed)	Liraglutid e 1.8 mg QD	Placebo	26 weeks
Jensterle_a,2017	Phase 3	2017	NCT029099933	Type A phenotype of PCOS diagnosed by ASRM-ESHRE Rotter- dam criteria including concomitant presence of a) hyper-androgenemia on either the biochemical or the clinical level, b) menses abnormalities and c) PCO morphology; age 18 years to menopause and obesity (body mass index: BMI ≥ 30).	Patients with history of carcinoma, sig-nificant cardiovascular, kidney or hepatic disease and the use of medications known to affect reproductive or metabolic functions within prior to study entry were excluded.	30 (28 completed)	Liraglutid e (1.2 mg QD) + Metformi n (1000 mg BID)	Liraglutide 3 mg QD	12 weeks
Zahra,2016	Phase 3	2016	NR	Women with PCOS diagnosed using the ESHRE/ASRM Rotterdam criteria	NR	60 (40 completed)	Metformi n (500 mg TID)	Placebo	

Nguyen,2023	Phase 3	2023	NR	Women aged 18 to 40 years with PCOS according to the Rotterdam criteria:	Congenital adrenal hyperplasia and androgen production-producing tumors, Cushing's disease, women with a history of ovarian surgery, ovarian tumours, ovarian endometriosis, or ovarian failure, obstruction of both fallopian tubes, and severe oligoasthenoteratozoospermia.	171 (132 completed)	Myo-inositol (500 mg QID)	Metformin (850 mg BID)	12 weeks
Ravn, 2022	Phase 3	2022	NR	PCOS diagnosed according to the Rotterdam criteria and between 18–50 years of age.	Other causes of oligomenorrhea and/or hirsutism including abnormal values of prolactin, thyroid stimu- lating hormone, or 17-hydroxy-progesterone, postmenopausal values of FSH (>25 IE/L), and type 1 or 2 diabetes mellitus.	45 (28 completed)	Myo-inositol (2 mg BID) + Folic acid (200 µg BID)	Metformin (500 mg BID)	24 weeks
Zheng,2017	Phase 3	2017	ChiCTR-IIR-16008084	18-40 years old and conformed to the revised 2003 Rotterdam Eu- ropean Society of Human Reproduction and Embryology (ESHRE)/ American Society of Reproductive	Other diseases that cause hyper-androgenemia and abnormal ovulation such as adrenal cortical hy-perplasia (CAH), Cushing's syndrome, hyperprolactinemia and testosterone-secreting tumors.	82 (63 completed)	Exenatide (10 µg BID)	Metformin (1000 mg BID)	12 weeks

				Medicine (ASRM) PCOS Consensus Workshop Group diagnostic criteria. imultaneously, women also needed to conform to criteria of the Obesity Problem Working Group of China (Zhou, 2002); i.e., women with a body mass index (BMI)!24 kg/m2 were considered to be overweight and women with a BMI!28 kg/m2 were considered to be obese.					
Jensterle_b,2016	Phase 3	2016	NCT02483299	All subjects had PCOS phenotype A, which included the concomitant presence of a) hyperandrogenemia at either the biochemical or clinical level, b) menstrual abnormalities, and c) PCO morphology. The subjects	A history of carcinoma, signifi-cant cardiovascular, kidney or hepatic disease, and the use of medications known to affect reproductive or metabolic functions prior to study entry. Certain subjects did, however, use oral contraceptives as advised by their	44 (43 completed)	Liraglutide 1.2 mg QD	Metformin (1000 mg BID) + Liraglutide (1.2 mg QD)	12 weeks

				were eligible for enroll- ment if they were aged >18 years, had yet to undergo the menopause, and were obese [body mass index (BMI) ≥30].	gynecologists >6 months prior to being recruited.				
Jensterle_c ,2015	Phase 3	2015	NCT01899430	Women with diagnosed PCOS were eligible for enrollment if they were aged 18 years to menopause and were obese (body mass index: BMI ≥30).	Known type 1 or type 2 diabetes mellitus, history of carcinoma, personal or family history of MEN 2, significant cardiovascular, kidney or hepatic disease and the use of medications known or suspected to affect reproductive or metabolic functions, or statins within 90 days prior to study entry.	32 (17 in Liraglutide group, 15 in Metformin group)	Liraglutid e (1.2 mg QD)	Metformin (1000 mg BID)	12 weeks

4. Patient Characteristics Table

Study ID	Age (mean, SD)	Male (%)	HbA1c (%)	BMI (kg/m^2)	Duration of Diabetes (years)
Wen,2023	26.75 ± 4.42	0%	NR	28.65 ± 1.93	0
Xing,2022	23.52 ± 4.65	0%	NR	28.80 ± 4.25	0
Elkind-Hirsch,2022	NR	0%	NR	41.60 ± 1.10	0
Ma,2021	30.10 ± 4.52	0%	NR	31.51 ± 4.20	0
Soldat-Stanković,2022	NR	0%	NR	30.50 ± 3.52	0
Tao,2021	NR	0%	NR	30.72 ± 4.20	0
Zheng,2019	27.20 ± 1.76	0%	NR	28.27 ± 2.2	0
Shorkpour,2019	28.30 ± 4.90	0%	NR	28.10 ± 3.10	0
Frøssing,2018	NR	0%	NR	33.30 ± 5.10	0
Jensterle,2017	31.60 ± 5.90	0%	NR	37.50 ± 5.30	0
Zahra,2016	25.80 ± 6.10	0%	NR	26.70 ± 6.50	0
Nguyen,2023	28.50 ± 3.20	0%	5.04 ± 1.45	20.69 ± 2.60	0
Ravn,2022	27.00 ± 9.75	0%	32.67 ± 4.88	34.10 ± 5.12	0
Zheng,2017	27.20 ± 3.10	0%	NR	28.27 ± 4.85	0
Jensterle,2016	30.30 ± 4.60	0%	NR	36.70 ± 5.10	0
Jensterle,2015	29.50 ± 7.70	0%	NR	41.60 ± 5.30	0

5. Risk of Bias Table for each outcome

Study ID	Random Sequence Generation	Allocation Concealment	Blinding of Participants/Personnel	Blinding of Outcome Assessment	Incomplete Outcome Data	Selective Reporting	Other Bias	Overall Judgment
Wen,2023	Low Risk	High Risk	High Risk	High Risk	Low Risk	Low Risk	None	High Risk
Xing,2022	Low Risk	Low Risk	High Risk	High Risk	Low Risk	Low Risk	None	High Risk
Elkind-Hirsch, 2022	Low Risk	Low Risk	Low Risk	Low Risk	Low Risk	Low Risk	None	Low Risk
Ma,2021	Low Risk	Low Risk	High Risk	High Risk	Low Risk	Low Risk	None	High Risk
Soldat-Stanković,2022	High Risk	Low Risk	High Risk	High Risk	Low Risk	Low Risk	None	High Risk
Tao,2021	Low Risk	High Risk	High Risk	High Risk	Low Risk	Low Risk	None	High Risk
Zheng,2019	Low Risk	High Risk	High Risk	High Risk	Low Risk	Low Risk	None	High Risk
Shokrpour,2019	Low Risk	Low Risk	Low Risk	High Risk	Low Risk	Low Risk	None	Some Concerns
Frøssing,2018	Low Risk	High Risk	Low Risk	High Risk	Low Risk	Low Risk	None	High Risk
Jensterle,2017	Low Risk	High Risk	High Risk	High Risk	Low Risk	Low Risk	None	High Risk
Zahra,2016	Low Risk	High Risk	High Risk	High Risk	Low Risk	Low Risk	None	High Risk
Nguyen,2023	Low Risk	Low Risk	High Risk	High Risk	Low Risk	Low Risk	None	High Risk
Ravn,2022	Low Risk	Low Risk	High Risk	High Risk	Low Risk	Low Risk	None	High Risk
Zheng,2017	Low Risk	Low Risk	High Risk	High Risk	Low Risk	Low Risk	None	High Risk
Jensterle,2016	Low Risk	Low Risk	High Risk	High Risk	Low Risk	Low Risk	None	High Risk
Jensterle,2015	Low Risk	Low Risk	High Risk	High Risk	Low Risk	Low Risk	None	High Risk

6. Funnel Plots

Weight Loss

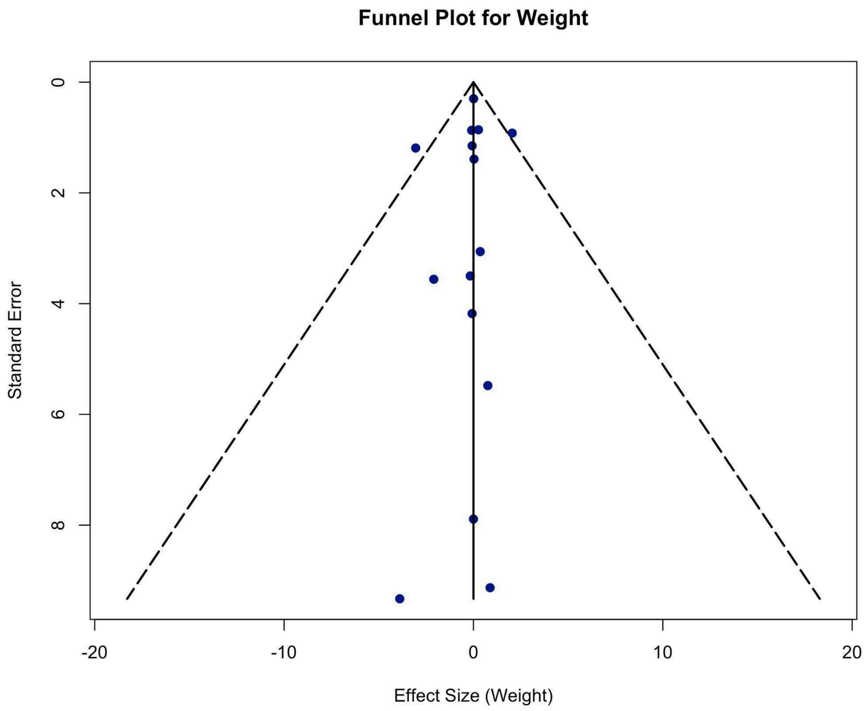

BMI Change

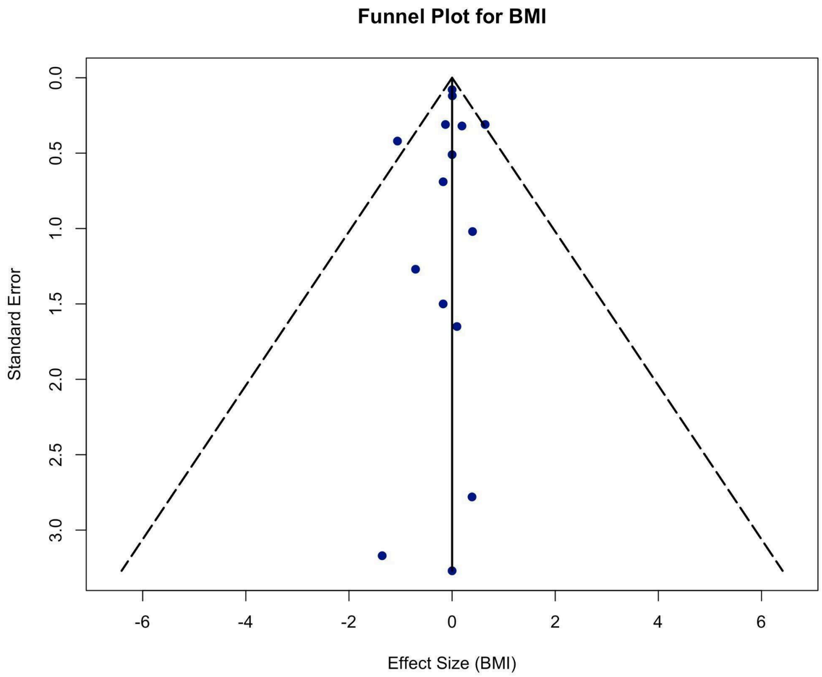

Waist Circumference Change

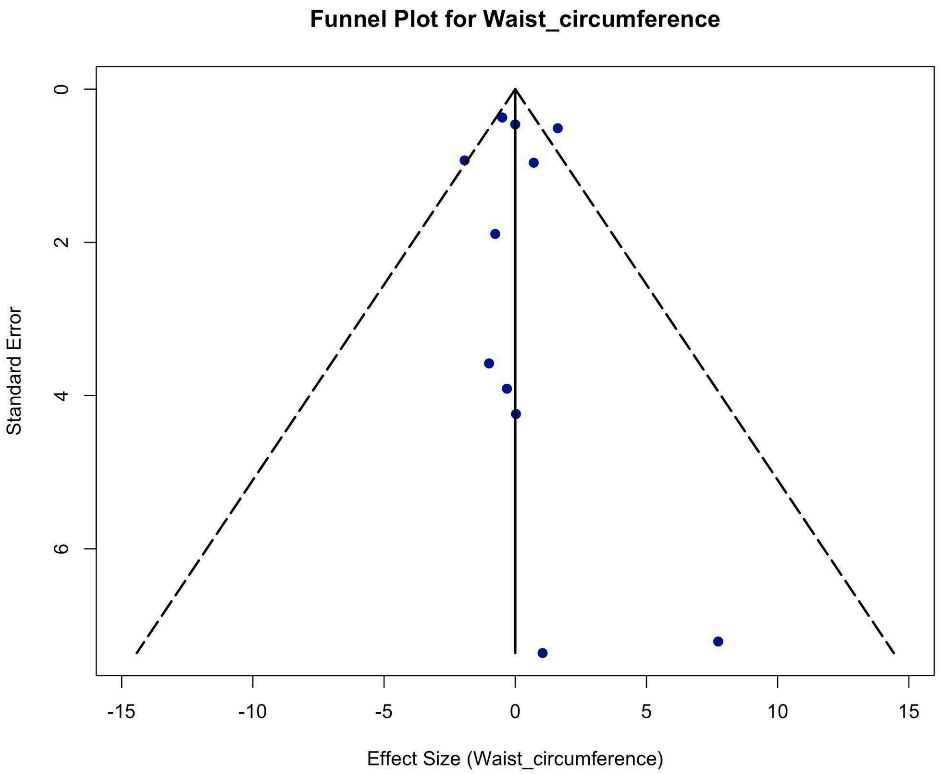

HOMA-IR Change

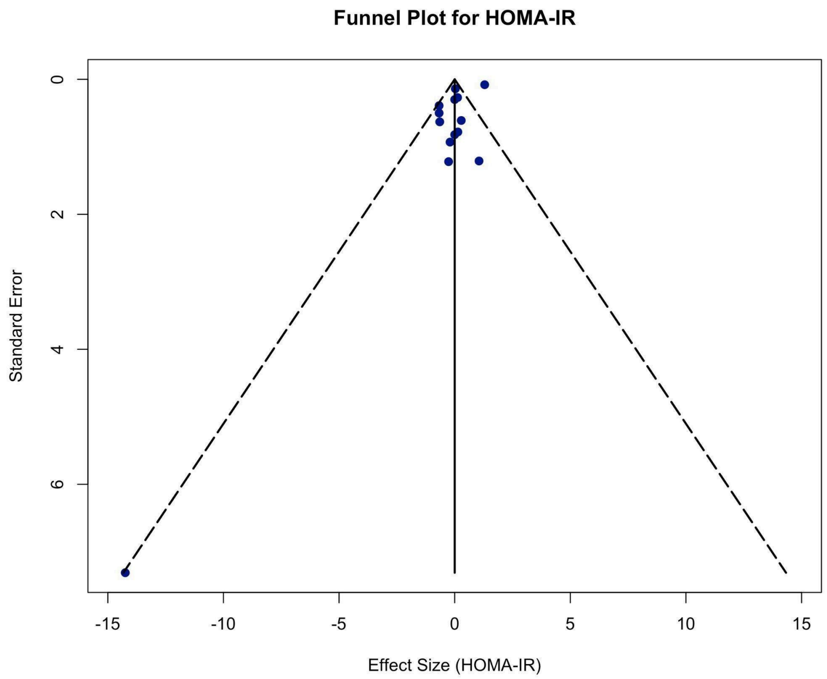

7. League Table for Each

Outcome Weight Loss

	GLP-1 + Metformin	GLP-1	Myoinositol + Folic acid	Metformin	Placebo
**GLP-1 + Metformin**	GLP-1 + Metformin	-0.49 (-1.92; 0.93)	.	-1.95 (-3.13; -0.77)	.
**GLP-1**	-0.45 (-1.67; 0.76)	GLP-1	.	-1.59 (-3.62; 0.44)	-5.23 (-7.76; -2.70)
**Myoinositol + Folic acid**	-1.88 (-3.09; -0.66)	-1.42 (-2.91; 0.06)	Myoinositol + Folic acid	-0.10 (-0.69; 0.49)	.
**Metformin**	-1.98 (-3.04; -0.91)	-1.52 (-2.88; -0.16)	-0.10 (-0.69; 0.49)	Metformin	-3.15 (-18.61; 12.31)
**Placebo**	-5.67 (-8.44; -2.90)	-5.21 (-7.71; -2.71)	-3.79 (-6.68; -0.90)	-3.69 (-6.52; -0.86)	Placebo

BMI Change

	GLP-1 + Metformin	GLP-1	Myoinositol + Folic acid	Metformin	Placebo
**GLP-1 + Metformin**	GLP-1 + Metformin	-0.20 (-0.69; 0.29)	.	-0.82 (-1.25; -0.39)	.
**GLP-1**	-0.21 (-0.65; 0.24)	GLP-1	.	-0.59 (-1.62; 0.44)	-2.00 (-2.15; -1.85)
**Myoinositol + Folic acid**	-0.71 (-1.18; -0.25)	-0.51 (-1.11; 0.09)	Myoinositol + Folic acid	-0.10 (-0.33; 0.14)	.
**Metformin**	-0.81 (-1.21; -0.41)	-0.61 (-1.16; -0.06)	-0.10 (-0.33; 0.14)	Metformin	-1.50 (-7.91; 4.91)
**Placebo**	-2.21 (-2.68; -1.73)	-2.00 (-2.15; -1.85)	-1.49 (-2.11; -0.88)	-1.39 (-1.96; -0.82)	Placebo

Waist Circumference Change

	GLP-1	Myoinositol + Folic acid	GLP-1 + Metformin	Metformin	Placebo
**GLP-1**	GLP-1	.	-1.45 (-5.04; 2.13)	-2.86 (-9.63; 3.91)	-5.04 (-5.72; -4.37)
**Myoinositol + Folic acid**	-1.04 (-4.40; 2.32)	Myoinositol + Folic acid	.	-1.66 (-2.56; -0.76)	.
**GLP-1 + Metformin**	-1.50 (-4.67; 1.67)	-0.46 (-1.70; 0.79)	GLP-1 + Metformin	-1.20 (-2.07; -0.33)	.
**Metformin**	-2.70 (-5.94; 0.54)	-1.66 (-2.56; -0.76)	-1.20 (-2.07; -0.34)	Metformin	.
**Placebo**	-5.04 (-5.72; -4.37)	-4.01 (-7.43; -0.58)	-3.55 (-6.79; -0.30)	-2.35 (-5.65; 0.96)	Placebo

HOMA-IR Change

	GLP-1 + Metformin	Myoinositol + Folic acid	Metformin	GLP-1	Placebo
**GLP-1 + Metformin**	GLP-1 + Metformin	.	-0.67 (-0.94; -0.40)	-0.06 (-1.16; 1.03)	.
**Myoinositol + Folic acid**	-0.26 (-0.81; 0.29)	Myoinositol + Folic acid	-0.31 (-0.79; 0.17)	.	.
**Metformin**	-0.57 (-0.83; -0.31)	-0.31 (-0.79; 0.17)	Metformin	-1.23 (-1.38; -1.08)	0.90 (-0.71; 2.51)
**GLP-1**	-1.75 (-2.04; -1.45)	-1.48 (-1.99; -0.98)	-1.17 (-1.32; -1.03)	GLP-1	-0.80 (-1.39; -0.21)
**Placebo**	-2.21 (-2.83; -1.58)	-1.95 (-2.69; -1.20)	-1.64 (-2.20; -1.07)	-0.46 (-1.01; 0.09)	Placebo

8. Treatment Ranking for Each

Outcome Weight Loss

=== Treatment Ranking ===

	P-score
GLP-1 + Metformin	0.9191
GLP-1	0.8044
Myoinositol + Folic acid	0.4072
Metformin	0.3633
Placebo	0.0061

BMI Change

=== Treatment Ranking ===

	P-score
GLP-1 + Metformin	0.9366
GLP-1	0.7893
Myoinositol + Folic acid	0.4403
Metformin	0.3338
Placebo	0.0000

Waist Circumference Change

=== Treatment Ranking ===

	P-score
GLP-1	0.8468
GLP-1 + Metformin	0.6916
Myoinositol + Folic acid	0.6239
Metformin	0.2386
Placebo	0.0990

HOMA-IR Change

=== Treatment Ranking ===

	P-score
GLP-1 + Metformin	0.7419
Myoinositol + Folic acid	0.4931
GLP-1	0.4858
Placebo	0.4145
Metformin	0.3646

9. Certainty of Evidence of Each Outcome

Summary of Evidence (GRADE)

Outcome	Certainty of Evidence	Reasons for Downgrade
Weight change	Very low	Within-study bias, Imprecision, Heterogeneity
BMI change	Low	Within-study bias
Waist circumference	Very low	Within-study bias, Reporting bias, Imprecision, Heterogeneity
HOMA-IR	Very low	Within-study bias, Reporting bias, Imprecision, Heterogeneity

10. Sensitivity Analysis

Weight Change

=== Sensitivity Analysis Results ===

=== Excluding High Risk of Bias (rob != 3) ===
❌ Not enough data to run network meta-analysis for low-risk studies.

=== Leave-One-Out Analysis ===
[[1]]
Original data:

	treat1	treat2	TE	seTE
Xing,2022	GLP-1 + Metformin	Metformin	-4.0700	3.5600
Elkind-Hirsch,2022	GLP-1	Placebo	-5.2000	1.3900
Ma,2021	GLP-1 + Metformin	Metformin	-1.7100	0.8600
Soldat-Stanković,2022	Metformin	Myoinositol + Folic acid	0.8500	5.4800
Tao,2021	GLP-1	Metformin	-1.2200	3.0600
Zheng,2019	GLP-1	Metformin	-1.6500	1.1500
Shokrpour,2019	Metformin	Myoinositol + Folic acid	0.1000	0.3000
Frøssing,2018	GLP-1	Placebo	-5.4000	3.5000
Jensterle,2017	GLP-1	GLP-1 + Metformin	-2.7000	1.1900
Zahra,2016	Metformin	Placebo	-3.1500	7.8900
Ravn,2022	Metformin	Myoinositol + Folic acid	-3.8000	9.3300
Zheng,2017	GLP-1	Metformin	-1.6500	4.1800
Jensterle,2016	GLP-1	GLP-1 + Metformin	2.4000	0.9200
Jensterle,2015	GLP-1	Metformin	-0.7000	9.1300

Number of treatment arms (by study):

	narms
Xing,2022	2
Elkind-Hirsch,2022	2
Ma,2021	2
Soldat-Stanković,2022	2
Tao,2021	2
Zheng,2019	2
Shokrpour,2019	2
Frøssing,2018	2
Jensterle,2017	2
Zahra,2016	2
Ravn,2022	2
Zheng,2017	2
Jensterle,2016	2

Jensterle,2015 2

Results (random effects model):

	treat1	treat2	MD	95%-CI
Xing,2022	GLP-1 + Metformin	Metformin	-1.8990	[-3.7749; -0.0232]
Elkind-Hirsch,2022	GLP-1	Placebo	-5.2196	[-8.1416; -2.2975]
Ma,2021	GLP-1 + Metformin	Metformin	-1.8990	[-3.7749; -0.0232]
Soldat-Stanković,2022	Metformin	Myoinositol + Folic acid	0.0817	[-1.7911; 1.9544]
Tao,2021	GLP-1	Metformin	-1.6431	[-3.5532; 0.2669]
Zheng,2019	GLP-1	Metformin	-1.6431	[-3.5532; 0.2669]
Shokrpour,2019	Metformin	Myoinositol + Folic acid	0.0817	[-1.7911; 1.9544]
Frøssing,2018	GLP-1	Placebo	-5.2196	[-8.1416; -2.2975]
Jensterle,2017	GLP-1	GLP-1 + Metformin	0.2559	[-1.4259; 1.9377]
Zahra,2016	Metformin	Placebo	-3.5764	[-7.0304; -0.1225]
Ravn,2022	Metformin	Myoinositol + Folic acid	0.0817	[-1.7911; 1.9544]
Zheng,2017	GLP-1	Metformin	-1.6431	[-3.5532; 0.2669]
Jensterle,2016	GLP-1	GLP-1 + Metformin	0.2559	[-1.4259; 1.9377]
Jensterle,2015	GLP-1	Metformin	-1.6431	[-3.5532; 0.2669]

Number of studies: k = 14
Number of pairwise comparisons: m = 14
Number of treatments: n = 5
Number of designs: d = 6

Random effects model

Treatment estimate (sm = 'MD', comparison: other treatments vs 'Placebo'):

	MD	95%-CI	z	p-value
GLP-1	-5.2196	[-8.1416; -2.2975]	-3.50	0.0005
GLP-1 + Metformin	-5.4755	[-8.8314; -2.1195]	-3.20	0.0014
Metformin	-3.5764	[-7.0304; -0.1225]	-2.03	0.0424
Myoinositol + Folic acid	-3.6581	[-7.5871; 0.2709]	-1.82	0.0680
Placebo	.	.	.	.

Quantifying heterogeneity / inconsistency:
tau^2 = 0.8610; tau = 0.9279; I^2 = 17.8% [0.0%; 57.9%]

Tests of heterogeneity (within designs) and inconsistency (between designs):

	Q	d.f.	p-value
Total	12.17	10	0.2741
Within designs	12.13	8	0.1453
Between designs	0.03	2	0.9848

Details of network meta-analysis methods:
- Frequentist graph-theoretical approach
- DerSimonian-Laird estimator for tau^2
- Calculation of I^2 based on Q

[[2]]
Original data:

	treat1	treat2	TE	seTE
Wen,2023	GLP-1 + Metformin	Metformin	-2.0700	0.8700
Elkind-Hirsch,2022	GLP-1	Placebo	-5.2000	1.3900
Ma,2021	GLP-1 + Metformin	Metformin	-1.7100	0.8600
Soldat-Stanković,2022	Metformin	Myoinositol + Folic acid	0.8500	5.4800
Tao,2021	GLP-1	Metformin	-1.2200	3.0600
Zheng,2019	GLP-1	Metformin	-1.6500	1.1500
Shokrpour,2019	Metformin	Myoinositol + Folic acid	0.1000	0.3000
Frøssing,2018	GLP-1	Placebo	-5.4000	3.5000
Jensterle,2017	GLP-1	GLP-1 + Metformin	-2.7000	1.1900
Zahra,2016	Metformin	Placebo	-3.1500	7.8900
Ravn,2022	Metformin	Myoinositol + Folic acid	-3.8000	9.3300
Zheng,2017	GLP-1	Metformin	-1.6500	4.1800
Jensterle,2016	GLP-1	GLP-1 + Metformin	2.4000	0.9200
Jensterle,2015	GLP-1	Metformin	-0.7000	9.1300

Number of treatment arms (by study):

	narms
Wen,2023	2
Elkind-Hirsch,2022	2
Ma,2021	2
Soldat-Stanković,2022	2
Tao,2021	2
Zheng,2019	2
Shokrpour,2019	2
Frøssing,2018	2
Jensterle,2017	2
Zahra,2016	2
Ravn,2022	2
Zheng,2017	2
Jensterle,2016	2
Jensterle,2015	2

Results (random effects model):

	treat1	treat2	MD	95%-CI

Wen,2023	GLP-1 + Metformin	Metformin	-1.8866	[-3.2507; -0.5225]
Elkind-Hirsch,2022	GLP-1	Placebo	-5.2165	[-7.9742; -2.4588]
Ma,2021	GLP-1 + Metformin	Metformin	-1.8866	[-3.2507; -0.5225]
Soldat-Stanković,2022	Metformin	Myoinositol + Folic acid	0.0885	[-1.3993; 1.5762]
Tao,2021	GLP-1	Metformin	-1.5889	[-3.2228; 0.0451]
Zheng,2019	GLP-1	Metformin	-1.5889	[-3.2228; 0.0451]
Shokrpour,2019	Metformin	Myoinositol + Folic acid	0.0885	[-1.3993; 1.5762]
Frøssing,2018	GLP-1	Placebo	-5.2165	[-7.9742; -2.4588]
Jensterle,2017	GLP-1	GLP-1 + Metformin	0.2977	[-1.1771; 1.7725]
Zahra,2016	Metformin	Placebo	-3.6276	[-6.8066; -0.4486]
Ravn,2022	Metformin	Myoinositol + Folic acid	0.0885	[-1.3993; 1.5762]
Zheng,2017	GLP-1	Metformin	-1.5889	[-3.2228; 0.0451]
Jensterle,2016	GLP-1	GLP-1 + Metformin	0.2977	[-1.1771; 1.7725]
Jensterle,2015	GLP-1	Metformin	-1.5889	[-3.2228; 0.0451]

Number of studies: k = 14
Number of pairwise comparisons: m = 14
Number of treatments: n = 5
Number of designs: d = 6

Random effects model

Treatment estimate (sm = 'MD', comparison: other treatments vs 'Placebo'):

	MD	95%-CI	z	p-value
GLP-1	-5.2165	[-7.9742; -2.4588]	-3.71	0.0002
GLP-1 + Metformin	-5.5142	[-8.6264; -2.4020]	-3.47	0.0005
Metformin	-3.6276	[-6.8066; -0.4486]	-2.24	0.0253
Myoinositol + Folic acid	-3.7161	[-7.2260; -0.2062]	-2.08	0.0380
Placebo	.	.	.	.

Quantifying heterogeneity / inconsistency:
tau^2 = 0.5012; tau = 0.7080; I^2 = 15.5% [0.0%; 56.0%]

Tests of heterogeneity (within designs) and inconsistency (between designs):

	Q	d.f.	p-value
Total	11.83	10	0.2966
Within designs	11.81	8	0.1601
Between designs	0.02	2	0.9879

Details of network meta-analysis methods:
- Frequentist graph-theoretical approach
- DerSimonian-Laird estimator for tau^2
- Calculation of I^2 based on Q

[[3]]
Original data:

	treat1	treat2	TE	seTE
Wen,2023	GLP-1 + Metformin	Metformin	-2.0700	0.8700
Xing,2022	GLP-1 + Metformin	Metformin	-4.0700	3.5600
Ma,2021	GLP-1 + Metformin	Metformin	-1.7100	0.8600
Soldat-Stanković,2022	Metformin	Myoinositol + Folic acid	0.8500	5.4800
Tao,2021	GLP-1	Metformin	-1.2200	3.0600
Zheng,2019	GLP-1	Metformin	-1.6500	1.1500
Shokrpour,2019	Metformin	Myoinositol + Folic acid	0.1000	0.3000
Frøssing,2018	GLP-1	Placebo	-5.4000	3.5000
Jensterle,2017	GLP-1	GLP-1 + Metformin	-2.7000	1.1900
Zahra,2016	Metformin	Placebo	-3.1500	7.8900
Ravn,2022	Metformin	Myoinositol + Folic acid	-3.8000	9.3300
Zheng,2017	GLP-1	Metformin	-1.6500	4.1800
Jensterle,2016	GLP-1	GLP-1 + Metformin	2.4000	0.9200
Jensterle,2015	GLP-1	Metformin	-0.7000	9.1300

Number of treatment arms (by study):

	narms
Wen,2023	2
Xing,2022	2
Ma,2021	2
Soldat-Stanković,2022	2
Tao,2021	2
Zheng,2019	2
Shokrpour,2019	2
Frøssing,2018	2
Jensterle,2017	2
Zahra,2016	2
Ravn,2022	2
Zheng,2017	2
Jensterle,2016	2
Jensterle,2015	2

Results (random effects model):

	treat1	treat2	MD	95%-CI
Wen,2023	GLP-1 + Metformin	Metformin	-1.9631	[-3.3511; -0.5751]
Xing,2022	GLP-1 + Metformin	Metformin	-1.9631	[-3.3511; -0.5751]
Ma,2021	GLP-1 + Metformin	Metformin	-1.9631	[-3.3511; -0.5751]
Soldat-Stanković,2022	Metformin	Myoinositol + Folic acid	0.0865	[-1.5221; 1.6952]

Tao,2021	GLP-1	Metformin -1.6533 [-3.3267; 0.0200]

```
Zheng,2019                   GLP-1             Metformin -1.6533 [ -3.3267; 0.0200]
Shokrpour,2019           Metformin Myoinositol + Folic acid 0.0865 [ -1.5221; 1.6952]
Frøssing,2018                GLP-1                Placebo -5.2987 [-11.7078; 1.1104]
Jensterle,2017               GLP-1       GLP-1 + Metformin 0.3098 [ -1.2083; 1.8279]
Zahra,2016               Metformin                Placebo -3.6454 [-10.1971; 2.9064]
Ravn,2022                Metformin Myoinositol + Folic acid 0.0865 [ -1.5221; 1.6952]
Zheng,2017                   GLP-1             Metformin -1.6533 [ -3.3267; 0.0200]
Jensterle,2016               GLP-1       GLP-1 + Metformin 0.3098 [ -1.2083; 1.8279]
Jensterle,2015               GLP-1             Metformin -1.6533 [ -3.3267; 0.0200]

Number of studies: k = 14
Number of pairwise comparisons: m = 14
Number of treatments: n = 5
Number of designs: d = 6

Random effects model

Treatment estimate (sm = 'MD', comparison: other treatments vs 'Placebo'):
                              MD            95%-CI     z p-value
GLP-1                    -5.2987 [-11.7078; 1.1104] -1.62 0.1051
GLP-1 + Metformin        -5.6085 [-12.1538; 0.9368] -1.68 0.0931
Metformin                -3.6454 [-10.1971; 2.9064] -1.09 0.2755
Myoinositol + Folic acid -3.7319 [-10.4782; 3.0144] -1.08 0.2783
Placebo                        .                  .     .       .

Quantifying heterogeneity / inconsistency:
tau^2 = 0.6042; tau = 0.7773; I^2 = 17.9% [0.0%; 58.0%]

Tests of heterogeneity (within designs) and inconsistency (between designs):
                    Q d.f. p-value
Total           12.18   10  0.2729
Within designs  12.17    8  0.1439
Between designs  0.02    2  0.9921

Details of network meta-analysis methods:
- Frequentist graph-theoretical approach
- DerSimonian-Laird estimator for tau^2
- Calculation of I^2 based on Q

[[4]]
Original data:

                       treat1          treat2      TE   seTE
Wen,2023    GLP-1 + Metformin       Metformin -2.0700 0.8700
```

Xing,2022 GLP-1 + Metformin Metformin -4.0700 3.5600
Elkind-Hirsch,2022 GLP-1 Placebo -5.2000 1.3900
Soldat-Stanković,2022 Metformin Myoinositol + Folic acid 0.8500 5.4800
Tao,2021 GLP-1 Metformin -1.2200 3.0600
Zheng,2019 GLP-1 Metformin -1.6500 1.1500
Shokrpour,2019 Metformin Myoinositol + Folic acid 0.1000 0.3000
Frøssing,2018 GLP-1 Placebo -5.4000 3.5000
Jensterle,2017 GLP-1 GLP-1 + Metformin -2.7000 1.1900
Zahra,2016 Metformin Placebo -3.1500 7.8900
Ravn,2022 Metformin Myoinositol + Folic acid -3.8000 9.3300
Zheng,2017 GLP-1 Metformin -1.6500 4.1800
Jensterle,2016 GLP-1 GLP-1 + Metformin 2.4000 0.9200
Jensterle,2015 GLP-1 Metformin -0.7000 9.1300

Number of treatment arms (by study):

	narms
Wen,2023	2
Xing,2022	2
Elkind-Hirsch,2022	2
Soldat-Stanković,2022	2
Tao,2021	2
Zheng,2019	2
Shokrpour,2019	2
Frøssing,2018	2
Jensterle,2017	2
Zahra,2016	2
Ravn,2022	2
Zheng,2017	2
Jensterle,2016	2
Jensterle,2015	2

Results (random effects model):

	treat1	treat2	MD	95%-CI
Wen,2023	GLP-1 + Metformin	Metformin	-2.1066	[-3.9623; -0.2508]
Xing,2022	GLP-1 + Metformin	Metformin	-2.1066	[-3.9623; -0.2508]
Elkind-Hirsch,2022	GLP-1	Placebo	-5.2236	[-8.1221; -2.3251]
Soldat-Stanković,2022	Metformin	Myoinositol + Folic acid	0.0827	[-1.7386; 1.9039]
Tao,2021	GLP-1	Metformin	-1.7660	[-3.6561; 0.1242]
Zheng,2019	GLP-1	Metformin	-1.7660	[-3.6561; 0.1242]
Shokrpour,2019	Metformin	Myoinositol + Folic acid	0.0827	[-1.7386; 1.9039]
Frøssing,2018	GLP-1	Placebo	-5.2236	[-8.1221; -2.3251]
Jensterle,2017	GLP-1	GLP-1 + Metformin	0.3406	[-1.3191; 2.0004]
Zahra,2016	Metformin	Placebo	-3.4577	[-6.8820; -0.0333]

```
Ravn,2022                    Metformin Myoinositol + Folic acid 0.0827 [-1.7386; 1.9039]
Zheng,2017                      GLP-1              Metformin -1.7660 [-3.6561; 0.1242]
Jensterle,2016                  GLP-1       GLP-1 + Metformin 0.3406 [-1.3191; 2.0004]
Jensterle,2015                  GLP-1              Metformin -1.7660 [-3.6561; 0.1242]

Number of studies: k = 14
Number of pairwise comparisons: m = 14
Number of treatments: n = 5
Number of designs: d = 6

Random effects model

Treatment estimate (sm = 'MD', comparison: other treatments vs 'Placebo'):
                               MD            95%-CI     z p-value
GLP-1                     -5.2236 [-8.1221; -2.3251] -3.53 0.0004
GLP-1 + Metformin         -5.5643 [-8.8893; -2.2392] -3.28 0.0010
Metformin                 -3.4577 [-6.8820; -0.0333] -1.98 0.0478
Myoinositol + Folic acid  -3.5403 [-7.4189; 0.3382] -1.79 0.0736
Placebo                         .                  .     .      .

Quantifying heterogeneity / inconsistency:
tau^2 = 0.8074; tau = 0.8985; I^2 = 16.8% [0.0%; 57.1%]

Tests of heterogeneity (within designs) and inconsistency (between designs):
                    Q d.f. p-value
Total           12.02  10 0.2834
Within designs  12.02   8 0.1504
Between designs  0.01   2 0.9962

Details of network meta-analysis methods:
- Frequentist graph-theoretical approach
- DerSimonian-Laird estimator for tau^2
- Calculation of I^2 based on Q

[[5]]
Original data:

                          treat1              treat2       TE   seTE
Wen,2023            GLP-1 + Metformin         Metformin -2.0700 0.8700
Xing,2022           GLP-1 + Metformin         Metformin -4.0700 3.5600
Elkind-Hirsch,2022              GLP-1           Placebo -5.2000 1.3900
Ma,2021             GLP-1 + Metformin         Metformin -1.7100 0.8600
Tao,2021                        GLP-1         Metformin -1.2200 3.0600
Zheng,2019                      GLP-1         Metformin -1.6500 1.1500
```

Shokrpour,2019	Metformin	Myoinositol + Folic acid	0.1000	0.3000
Frøssing,2018	GLP-1	Placebo	-5.4000	3.5000
Jensterle,2017	GLP-1	GLP-1 + Metformin	-2.7000	1.1900
Zahra,2016	Metformin	Placebo	-3.1500	7.8900
Ravn,2022	Metformin	Myoinositol + Folic acid	-3.8000	9.3300
Zheng,2017	GLP-1	Metformin	-1.6500	4.1800
Jensterle,2016	GLP-1	GLP-1 + Metformin	2.4000	0.9200
Jensterle,2015	GLP-1	Metformin	-0.7000	9.1300

Number of treatment arms (by study):

	narms
Wen,2023	2
Xing,2022	2
Elkind-Hirsch,2022	2
Ma,2021	2
Tao,2021	2
Zheng,2019	2
Shokrpour,2019	2
Frøssing,2018	2
Jensterle,2017	2
Zahra,2016	2
Ravn,2022	2
Zheng,2017	2
Jensterle,2016	2
Jensterle,2015	2

Results (random effects model):

	treat1	treat2	MD	95%-CI
Wen,2023	GLP-1 + Metformin	Metformin	-1.9629	[-3.3426; -0.5832]
Xing,2022	GLP-1 + Metformin	Metformin	-1.9629	[-3.3426; -0.5832]
Elkind-Hirsch,2022	GLP-1	Placebo	-5.2188	[-8.0169; -2.4207]
Ma,2021	GLP-1 + Metformin	Metformin	-1.9629	[-3.3426; -0.5832]
Tao,2021	GLP-1	Metformin	-1.6501	[-3.3144; 0.0142]
Zheng,2019	GLP-1	Metformin	-1.6501	[-3.3144; 0.0142]
Shokrpour,2019	Metformin	Myoinositol + Folic acid	0.0701	[-1.5361; 1.6763]
Frøssing,2018	GLP-1	Placebo	-5.2188	[-8.0169; -2.4207]
Jensterle,2017	GLP-1	GLP-1 + Metformin	0.3128	[-1.1971; 1.8228]
Zahra,2016	Metformin	Placebo	-3.5687	[-6.7967; -0.3407]
Ravn,2022	Metformin	Myoinositol + Folic acid	0.0701	[-1.5361; 1.6763]
Zheng,2017	GLP-1	Metformin	-1.6501	[-3.3144; 0.0142]
Jensterle,2016	GLP-1	GLP-1 + Metformin	0.3128	[-1.1971; 1.8228]
Jensterle,2015	GLP-1	Metformin	-1.6501	[-3.3144; 0.0142]

Number of studies: k = 14
Number of pairwise comparisons: m = 14
Number of treatments: n = 5
Number of designs: d = 6

Random effects model

Treatment estimate (sm = 'MD', comparison: other treatments vs 'Placebo'):

	MD	95%-CI	z	p-value
GLP-1	-5.2188	[-8.0169; -2.4207]	-3.66	0.0003
GLP-1 + Metformin	-5.5316	[-8.6951; -2.3681]	-3.43	0.0006
Metformin	-3.5687	[-6.7967; -0.3407]	-2.17	0.0302
Myoinositol + Folic acid	-3.6388	[-7.2443; -0.0333]	-1.98	0.0479
Placebo	.	.	.	.

Quantifying heterogeneity / inconsistency:
tau^2 = 0.5868; tau = 0.7660; I^2 = 17.8% [0.0%; 57.9%]

Tests of heterogeneity (within designs) and inconsistency (between designs):

	Q	d.f.	p-value
Total	12.17	10	0.2741
Within designs	12.15	8	0.1445
Between designs	0.01	2	0.9933

Details of network meta-analysis methods:
- Frequentist graph-theoretical approach
- DerSimonian-Laird estimator for tau^2
- Calculation of I^2 based on Q

[[6]]
Original data:

	treat1	treat2	TE	seTE
Wen,2023	GLP-1 + Metformin	Metformin	-2.0700	0.8700
Xing,2022	GLP-1 + Metformin	Metformin	-4.0700	3.5600
Elkind-Hirsch,2022	GLP-1	Placebo	-5.2000	1.3900
Ma,2021	GLP-1 + Metformin	Metformin	-1.7100	0.8600
Soldat-Stanković,2022	Metformin	Myoinositol + Folic acid	0.8500	5.4800
Zheng,2019	GLP-1	Metformin	-1.6500	1.1500
Shokrpour,2019	Metformin	Myoinositol + Folic acid	0.1000	0.3000
Frøssing,2018	GLP-1	Placebo	-5.4000	3.5000
Jensterle,2017	GLP-1	GLP-1 + Metformin	-2.7000	1.1900
Zahra,2016	Metformin	Placebo	-3.1500	7.8900
Ravn,2022	Metformin	Myoinositol + Folic acid	-3.8000	9.3300

Zheng,2017	GLP-1	Metformin	-1.6500	4.1800
Jensterle,2016	GLP-1	GLP-1 + Metformin	2.4000	0.9200
Jensterle,2015	GLP-1	Metformin	-0.7000	9.1300

Number of treatment arms (by study):

	narms
Wen,2023	2
Xing,2022	2
Elkind-Hirsch,2022	2
Ma,2021	2
Soldat-Stanković,2022	2
Zheng,2019	2
Shokrpour,2019	2
Frøssing,2018	2
Jensterle,2017	2
Zahra,2016	2
Ravn,2022	2
Zheng,2017	2
Jensterle,2016	2
Jensterle,2015	2

Results (random effects model):

	treat1	treat2	MD	95%-CI
Wen,2023	GLP-1 + Metformin	Metformin	-1.9774	[-3.3792; -0.5757]
Xing,2022	GLP-1 + Metformin	Metformin	-1.9774	[-3.3792; -0.5757]
Elkind-Hirsch,2022	GLP-1	Placebo	-5.2200	[-8.0256; -2.4144]
Ma,2021	GLP-1 + Metformin	Metformin	-1.9774	[-3.3792; -0.5757]
Soldat-Stanković,2022	Metformin	Myoinositol + Folic acid	0.0865	[-1.5204; 1.6935]
Zheng,2019	GLP-1	Metformin	-1.6862	[-3.4221; 0.0497]
Shokrpour,2019	Metformin	Myoinositol + Folic acid	0.0865	[-1.5204; 1.6935]
Frøssing,2018	GLP-1	Placebo	-5.2200	[-8.0256; -2.4144]
Jensterle,2017	GLP-1	GLP-1 + Metformin	0.2912	[-1.2489; 1.8314]
Zahra,2016	Metformin	Placebo	-3.5338	[-6.8031; -0.2645]
Ravn,2022	Metformin	Myoinositol + Folic acid	0.0865	[-1.5204; 1.6935]
Zheng,2017	GLP-1	Metformin	-1.6862	[-3.4221; 0.0497]
Jensterle,2016	GLP-1	GLP-1 + Metformin	0.2912	[-1.2489; 1.8314]
Jensterle,2015	GLP-1	Metformin	-1.6862	[-3.4221; 0.0497]

Number of studies: k = 14
Number of pairwise comparisons: m = 14
Number of treatments: n = 5
Number of designs: d = 6

Random effects model

Treatment estimate (sm = 'MD', comparison: other treatments vs 'Placebo'):

	MD	95%-CI	z	p-value
GLP-1	-5.2200	[-8.0256; -2.4144]	-3.65	0.0003
GLP-1 + Metformin	-5.5112	[-8.6943; -2.3282]	-3.39	0.0007
Metformin	-3.5338	[-6.8031; -0.2645]	-2.12	0.0341
Myoinositol + Folic acid	-3.6204	[-7.2632; 0.0225]	-1.95	0.0514
Placebo	.	.	.	.

Quantifying heterogeneity / inconsistency:
tau^2 = 0.6027; tau = 0.7763; I^2 = 17.9% [0.0%; 58.0%]

Tests of heterogeneity (within designs) and inconsistency (between designs):

	Q	d.f.	p-value
Total	12.17	10	0.2736
Within designs	12.15	8	0.1444
Between designs	0.02	2	0.9902

Details of network meta-analysis methods:
- Frequentist graph-theoretical approach
- DerSimonian-Laird estimator for tau^2
- Calculation of I^2 based on Q

[[7]]
Original data:

	treat1	treat2	TE	seTE
Wen,2023	GLP-1 + Metformin	Metformin	-2.0700	0.8700
Xing,2022	GLP-1 + Metformin	Metformin	-4.0700	3.5600
Elkind-Hirsch,2022	GLP-1	Placebo	-5.2000	1.3900
Ma,2021	GLP-1 + Metformin	Metformin	-1.7100	0.8600
Soldat-Stanković,2022	Metformin	Myoinositol + Folic acid	0.8500	5.4800
Tao,2021	GLP-1	Metformin	-1.2200	3.0600
Shokrpour,2019	Metformin	Myoinositol + Folic acid	0.1000	0.3000
Frøssing,2018	GLP-1	Placebo	-5.4000	3.5000
Jensterle,2017	GLP-1	GLP-1 + Metformin	-2.7000	1.1900
Zahra,2016	Metformin	Placebo	-3.1500	7.8900
Ravn,2022	Metformin	Myoinositol + Folic acid	-3.8000	9.3300
Zheng,2017	GLP-1	Metformin	-1.6500	4.1800
Jensterle,2016	GLP-1	GLP-1 + Metformin	2.4000	0.9200
Jensterle,2015	GLP-1	Metformin	-0.7000	9.1300

Number of treatment arms (by study):

	narms
Wen,2023	2
Xing,2022	2
Elkind-Hirsch,2022	2
Ma,2021	2
Soldat-Stanković,2022	2
Tao,2021	2
Shokrpour,2019	2
Frøssing,2018	2
Jensterle,2017	2
Zahra,2016	2
Ravn,2022	2
Zheng,2017	2
Jensterle,2016	2
Jensterle,2015	2

Results (random effects model):

	treat1	treat2	MD	95%-CI
Wen,2023	GLP-1 + Metformin	Metformin	-1.9665	[-3.5292; -0.4038]
Xing,2022	GLP-1 + Metformin	Metformin	-1.9665	[-3.5292; -0.4038]
Elkind-Hirsch,2022	GLP-1	Placebo	-5.2204	[-8.0895; -2.3513]
Ma,2021	GLP-1 + Metformin	Metformin	-1.9665	[-3.5292; -0.4038]
Soldat-Stanković,2022	Metformin	Myoinositol + Folic acid	0.0839	[-1.6709; 1.8387]
Tao,2021	GLP-1	Metformin	-1.6803	[-3.8737; 0.5131]
Shokrpour,2019	Metformin	Myoinositol + Folic acid	0.0839	[-1.6709; 1.8387]
Frøssing,2018	GLP-1	Placebo	-5.2204	[-8.0895; -2.3513]
Jensterle,2017	GLP-1	GLP-1 + Metformin	0.2862	[-1.4639; 2.0363]
Zahra,2016	Metformin	Placebo	-3.5401	[-7.1060; 0.0258]
Ravn,2022	Metformin	Myoinositol + Folic acid	0.0839	[-1.6709; 1.8387]
Zheng,2017	GLP-1	Metformin	-1.6803	[-3.8737; 0.5131]
Jensterle,2016	GLP-1	GLP-1 + Metformin	0.2862	[-1.4639; 2.0363]
Jensterle,2015	GLP-1	Metformin	-1.6803	[-3.8737; 0.5131]

Number of studies: k = 14
Number of pairwise comparisons: m = 14
Number of treatments: n = 5
Number of designs: d = 6

Random effects model

Treatment estimate (sm = 'MD', comparison: other treatments vs 'Placebo'):

	MD	95%-CI	z	p-value
GLP-1	-5.2204	[-8.0895; -2.3513]	-3.57	0.0004

GLP-1 + Metformin -5.5066 [-8.8397; -2.1734] -3.24 0.0012
Metformin -3.5401 [-7.1060; 0.0258] -1.95 0.0517
Myoinositol + Folic acid -3.6240 [-7.5983; 0.3503] -1.79 0.0739
Placebo . . . .

Quantifying heterogeneity / inconsistency:
tau^2 = 0.7409; tau = 0.8607; I^2 = 17.8% [0.0%; 57.9%]

Tests of heterogeneity (within designs) and inconsistency (between designs):

	Q	d.f.	p-value
Total	12.16	10	0.2742
Within designs	12.16	8	0.1444
Between designs	0.01	2	0.9955

Details of network meta-analysis methods:
- Frequentist graph-theoretical approach
- DerSimonian-Laird estimator for tau^2
- Calculation of I^2 based on Q

[[8]]
Original data:

	treat1	treat2	TE	seTE
Wen,2023	GLP-1 + Metformin	Metformin	-2.0700	0.8700
Xing,2022	GLP-1 + Metformin	Metformin	-4.0700	3.5600
Elkind-Hirsch,2022	GLP-1	Placebo	-5.2000	1.3900
Ma,2021	GLP-1 + Metformin	Metformin	-1.7100	0.8600
Soldat-Stanković,2022	Metformin	Myoinositol + Folic acid	0.8500	5.4800
Tao,2021	GLP-1	Metformin	-1.2200	3.0600
Zheng,2019	GLP-1	Metformin	-1.6500	1.1500
Frøssing,2018	GLP-1	Placebo	-5.4000	3.5000
Jensterle,2017	GLP-1	GLP-1 + Metformin	-2.7000	1.1900
Zahra,2016	Metformin	Placebo	-3.1500	7.8900
Ravn,2022	Metformin	Myoinositol + Folic acid	-3.8000	9.3300
Zheng,2017	GLP-1	Metformin	-1.6500	4.1800
Jensterle,2016	GLP-1	GLP-1 + Metformin	2.4000	0.9200
Jensterle,2015	GLP-1	Metformin	-0.7000	9.1300

Number of treatment arms (by study):

	narms
Wen,2023	2
Xing,2022	2
Elkind-Hirsch,2022	2
Ma,2021	2

Soldat-Stanković,2022	2
Tao,2021	2
Zheng,2019	2
Frøssing,2018	2
Jensterle,2017	2
Zahra,2016	2
Ravn,2022	2
Zheng,2017	2
Jensterle,2016	2
Jensterle,2015	2

Results (random effects model):

	treat1	treat2	MD	95%-CI
Wen,2023	GLP-1 + Metformin	Metformin	-1.9629	[-3.3442; -0.5815]
Xing,2022	GLP-1 + Metformin	Metformin	-1.9629	[-3.3442; -0.5815]
Elkind-Hirsch,2022	GLP-1	Placebo	-5.2188	[-8.0187; -2.4190]
Ma,2021	GLP-1 + Metformin	Metformin	-1.9629	[-3.3442; -0.5815]
Soldat-Stanković,2022	Metformin	Myoinositol + Folic acid	-0.3540	[-9.6906; 8.9825]
Tao,2021	GLP-1	Metformin	-1.6506	[-3.3165; 0.0154]
Zheng,2019	GLP-1	Metformin	-1.6506	[-3.3165; 0.0154]
Frøssing,2018	GLP-1	Placebo	-5.2188	[-8.0187; -2.4190]
Jensterle,2017	GLP-1	GLP-1 + Metformin	0.3123	[-1.1993; 1.8239]
Zahra,2016	Metformin	Placebo	-3.5682	[-6.7985; -0.3380]
Ravn,2022	Metformin	Myoinositol + Folic acid	-0.3540	[-9.6906; 8.9825]
Zheng,2017	GLP-1	Metformin	-1.6506	[-3.3165; 0.0154]
Jensterle,2016	GLP-1	GLP-1 + Metformin	0.3123	[-1.1993; 1.8239]
Jensterle,2015	GLP-1	Metformin	-1.6506	[-3.3165; 0.0154]

Number of studies: k = 14
Number of pairwise comparisons: m = 14
Number of treatments: n = 5
Number of designs: d = 6

Random effects model

Treatment estimate (sm = 'MD', comparison: other treatments vs 'Placebo'):

	MD	95%-CI	z	p-value
GLP-1	-5.2188	[-8.0187; -2.4190]	-3.65	0.0003
GLP-1 + Metformin	-5.5311	[-8.6968; -2.3654]	-3.42	0.0006
Metformin	-3.5682	[-6.7985; -0.3380]	-2.17	0.0304
Myoinositol + Folic acid	-3.2142	[-13.0938; 6.6653]	-0.64	0.5237
Placebo	.	.	.	.

Quantifying heterogeneity / inconsistency:
tau^2 = 0.5904; tau = 0.7684; I^2 = 17.9% [0.0%; 58.0%]

Tests of heterogeneity (within designs) and inconsistency (between designs):

	Q	d.f.	p-value
Total	12.18	10	0.2735
Within designs	12.16	8	0.1441
Between designs	0.01	2	0.9933

Details of network meta-analysis methods:
- Frequentist graph-theoretical approach
- DerSimonian-Laird estimator for tau^2
- Calculation of I^2 based on Q

[[9]]
Original data:

	treat1	treat2	TE	seTE
Wen,2023	GLP-1 + Metformin	Metformin	-2.0700	0.8700
Xing,2022	GLP-1 + Metformin	Metformin	-4.0700	3.5600
Elkind-Hirsch,2022	GLP-1	Placebo	-5.2000	1.3900
Ma,2021	GLP-1 + Metformin	Metformin	-1.7100	0.8600
Soldat-Stanković,2022	Metformin	Myoinositol + Folic acid	0.8500	5.4800
Tao,2021	GLP-1	Metformin	-1.2200	3.0600
Zheng,2019	GLP-1	Metformin	-1.6500	1.1500
Shokrpour,2019	Metformin	Myoinositol + Folic acid	0.1000	0.3000
Jensterle,2017	GLP-1	GLP-1 + Metformin	-2.7000	1.1900
Zahra,2016	Metformin	Placebo	-3.1500	7.8900
Ravn,2022	Metformin	Myoinositol + Folic acid	-3.8000	9.3300
Zheng,2017	GLP-1	Metformin	-1.6500	4.1800
Jensterle,2016	GLP-1	GLP-1 + Metformin	2.4000	0.9200
Jensterle,2015	GLP-1	Metformin	-0.7000	9.1300

Number of treatment arms (by study):

	narms
Wen,2023	2
Xing,2022	2
Elkind-Hirsch,2022	2
Ma,2021	2
Soldat-Stanković,2022	2
Tao,2021	2
Zheng,2019	2
Shokrpour,2019	2
Jensterle,2017	2

Zahra,2016	2
Ravn,2022	2
Zheng,2017	2
Jensterle,2016	2
Jensterle,2015	2

Results (random effects model):

	treat1	treat2	MD	95%-CI
Wen,2023	GLP-1 + Metformin	Metformin	-1.9626	[-3.3495; -0.5756]
Xing,2022	GLP-1 + Metformin	Metformin	-1.9626	[-3.3495; -0.5756]
Elkind-Hirsch,2022	GLP-1	Placebo	-5.1846	[-8.2446; -2.1245]
Ma,2021	GLP-1 + Metformin	Metformin	-1.9626	[-3.3495; -0.5756]
Soldat-Stanković,2022	Metformin	Myoinositol + Folic acid	0.0866	[-1.5203; 1.6934]
Tao,2021	GLP-1	Metformin	-1.6518	[-3.3231; 0.0196]
Zheng,2019	GLP-1	Metformin	-1.6518	[-3.3231; 0.0196]
Shokrpour,2019	Metformin	Myoinositol + Folic acid	0.0866	[-1.5203; 1.6934]
Jensterle,2017	GLP-1	GLP-1 + Metformin	0.3108	[-1.2062; 1.8277]
Zahra,2016	Metformin	Placebo	-3.5328	[-6.9883; -0.0773]
Ravn,2022	Metformin	Myoinositol + Folic acid	0.0866	[-1.5203; 1.6934]
Zheng,2017	GLP-1	Metformin	-1.6518	[-3.3231; 0.0196]
Jensterle,2016	GLP-1	GLP-1 + Metformin	0.3108	[-1.2062; 1.8277]
Jensterle,2015	GLP-1	Metformin	-1.6518	[-3.3231; 0.0196]

Number of studies: k = 14
Number of pairwise comparisons: m = 14
Number of treatments: n = 5
Number of designs: d = 6

Random effects model

Treatment estimate (sm = 'MD', comparison: other treatments vs 'Placebo'):

	MD	95%-CI	z	p-value
GLP-1	-5.1846	[-8.2446; -2.1245]	-3.32	0.0009
GLP-1 + Metformin	-5.4953	[-8.8927; -2.0980]	-3.17	0.0015
Metformin	-3.5328	[-6.9883; -0.0773]	-2.00	0.0451
Myoinositol + Folic acid	-3.6193	[-7.4302; 0.1915]	-1.86	0.0627
Placebo	.	.	.	.

Quantifying heterogeneity / inconsistency:
tau^2 = 0.6026; tau = 0.7763; I^2 = 17.9% [0.0%; 58.0%]

Tests of heterogeneity (within designs) and inconsistency (between designs):

Q d.f. p-value

Total	12.18	10	0.2731
Within designs	12.17	8	0.1439
Between designs	0.01	2	0.9935

Details of network meta-analysis methods:
- Frequentist graph-theoretical approach
- DerSimonian-Laird estimator for tau^2
- Calculation of I^2 based on Q

[[10]]
Original data:

	treat1	treat2	TE	seTE
Wen,2023	GLP-1 + Metformin	Metformin	-2.0700	0.8700
Xing,2022	GLP-1 + Metformin	Metformin	-4.0700	3.5600
Elkind-Hirsch,2022	GLP-1	Placebo	-5.2000	1.3900
Ma,2021	GLP-1 + Metformin	Metformin	-1.7100	0.8600
Soldat-Stanković,2022	Metformin	Myoinositol + Folic acid	0.8500	5.4800
Tao,2021	GLP-1	Metformin	-1.2200	3.0600
Zheng,2019	GLP-1	Metformin	-1.6500	1.1500
Shokrpour,2019	Metformin	Myoinositol + Folic acid	0.1000	0.3000
Frøssing,2018	GLP-1	Placebo	-5.4000	3.5000
Zahra,2016	Metformin	Placebo	-3.1500	7.8900
Ravn,2022	Metformin	Myoinositol + Folic acid	-3.8000	9.3300
Zheng,2017	GLP-1	Metformin	-1.6500	4.1800
Jensterle,2016	GLP-1	GLP-1 + Metformin	2.4000	0.9200
Jensterle,2015	GLP-1	Metformin	-0.7000	9.1300

Number of treatment arms (by study):

	narms
Wen,2023	2
Xing,2022	2
Elkind-Hirsch,2022	2
Ma,2021	2
Soldat-Stanković,2022	2
Tao,2021	2
Zheng,2019	2
Shokrpour,2019	2
Frøssing,2018	2
Zahra,2016	2
Ravn,2022	2
Zheng,2017	2
Jensterle,2016	2
Jensterle,2015	2

Results (random effects model):

	treat1	treat2	MD	95%-CI
Wen,2023	GLP-1 + Metformin	Metformin	-2.2787	[-3.3613; -1.1962]
Xing,2022	GLP-1 + Metformin	Metformin	-2.2787	[-3.3613; -1.1962]
Elkind-Hirsch,2022	GLP-1	Placebo	-5.1898	[-7.6888; -2.6908]
Ma,2021	GLP-1 + Metformin	Metformin	-2.2787	[-3.3613; -1.1962]
Soldat-Stanković,2022	Metformin	Myoinositol + Folic acid	0.0982	[-0.4886; 0.6850]
Tao,2021	GLP-1	Metformin	-0.6434	[-2.1148; 0.8280]
Zheng,2019	GLP-1	Metformin	-0.6434	[-2.1148; 0.8280]
Shokrpour,2019	Metformin	Myoinositol + Folic acid	0.0982	[-0.4886; 0.6850]
Frøssing,2018	GLP-1	Placebo	-5.1898	[-7.6888; -2.6908]
Zahra,2016	Metformin	Placebo	-4.5464	[-7.4268; -1.6660]
Ravn,2022	Metformin	Myoinositol + Folic acid	0.0982	[-0.4886; 0.6850]
Zheng,2017	GLP-1	Metformin	-0.6434	[-2.1148; 0.8280]
Jensterle,2016	GLP-1	GLP-1 + Metformin	1.6353	[0.2083; 3.0623]
Jensterle,2015	GLP-1	Metformin	-0.6434	[-2.1148; 0.8280]

Number of studies: k = 14
Number of pairwise comparisons: m = 14
Number of treatments: n = 5
Number of designs: d = 6

Random effects model

Treatment estimate (sm = 'MD', comparison: other treatments vs 'Placebo'):

	MD	95%-CI	z	p-value
GLP-1	-5.1898	[-7.6888; -2.6908]	-4.07	< 0.0001
GLP-1 + Metformin	-6.8251	[-9.6891; -3.9612]	-4.67	< 0.0001
Metformin	-4.5464	[-7.4268; -1.6660]	-3.09	0.0020
Myoinositol + Folic acid	-4.6446	[-7.5842; -1.7050]	-3.10	0.0020
Placebo	.	.	.	.

Quantifying heterogeneity / inconsistency:
tau^2 = 0; tau = 0; I^2 = 0% [0.0%; 60.2%]

Tests of heterogeneity (within designs) and inconsistency (between designs):

	Q	d.f.	p-value
Total	2.53	10	0.9905
Within designs	0.67	8	0.9996
Between designs	1.85	2	0.3961

Details of network meta-analysis methods:

- Frequentist graph-theoretical approach
- DerSimonian-Laird estimator for tau^2
- Calculation of I^2 based on Q

[[11]]
Original data:

	treat1	treat2	TE	seTE
Wen,2023	GLP-1 + Metformin	Metformin	-2.0700	0.8700
Xing,2022	GLP-1 + Metformin	Metformin	-4.0700	3.5600
Elkind-Hirsch,2022	GLP-1	Placebo	-5.2000	1.3900
Ma,2021	GLP-1 + Metformin	Metformin	-1.7100	0.8600
Soldat-Stanković,2022	Metformin	Myoinositol + Folic acid	0.8500	5.4800
Tao,2021	GLP-1	Metformin	-1.2200	3.0600
Zheng,2019	GLP-1	Metformin	-1.6500	1.1500
Shokrpour,2019	Metformin	Myoinositol + Folic acid	0.1000	0.3000
Frøssing,2018	GLP-1	Placebo	-5.4000	3.5000
Jensterle,2017	GLP-1	GLP-1 + Metformin	-2.7000	1.1900
Ravn,2022	Metformin	Myoinositol + Folic acid	-3.8000	9.3300
Zheng,2017	GLP-1	Metformin	-1.6500	4.1800
Jensterle,2016	GLP-1	GLP-1 + Metformin	2.4000	0.9200
Jensterle,2015	GLP-1	Metformin	-0.7000	9.1300

Number of treatment arms (by study):

	narms
Wen,2023	2
Xing,2022	2
Elkind-Hirsch,2022	2
Ma,2021	2
Soldat-Stanković,2022	2
Tao,2021	2
Zheng,2019	2
Shokrpour,2019	2
Frøssing,2018	2
Jensterle,2017	2
Ravn,2022	2
Zheng,2017	2
Jensterle,2016	2
Jensterle,2015	2

Results (random effects model):

	treat1	treat2	MD	95%-CI
Wen,2023	GLP-1 + Metformin	Metformin	-1.9608	[-3.3420; -0.5796]

Xing,2022	GLP-1 + Metformin	Metformin	-1.9608	[-3.3420; -0.5796]
Elkind-Hirsch,2022	GLP-1	Placebo	-5.2328	[-8.0763; -2.3893]
Ma,2021	GLP-1 + Metformin	Metformin	-1.9608	[-3.3420; -0.5796]
Soldat-Stanković,2022	Metformin	Myoinositol + Folic acid	0.0869	[-1.5005; 1.6742]
Tao,2021	GLP-1	Metformin	-1.6450	[-3.3181; 0.0280]
Zheng,2019	GLP-1	Metformin	-1.6450	[-3.3181; 0.0280]
Shokrpour,2019	Metformin	Myoinositol + Folic acid	0.0869	[-1.5005; 1.6742]
Frøssing,2018	GLP-1	Placebo	-5.2328	[-8.0763; -2.3893]
Jensterle,2017	GLP-1	GLP-1 + Metformin	0.3158	[-1.1969; 1.8285]
Ravn,2022	Metformin	Myoinositol + Folic acid	0.0869	[-1.5005; 1.6742]
Zheng,2017	GLP-1	Metformin	-1.6450	[-3.3181; 0.0280]
Jensterle,2016	GLP-1	GLP-1 + Metformin	0.3158	[-1.1969; 1.8285]
Jensterle,2015	GLP-1	Metformin	-1.6450	[-3.3181; 0.0280]

Number of studies: k = 14
Number of pairwise comparisons: m = 14
Number of treatments: n = 5
Number of designs: d = 5

Random effects model

Treatment estimate (sm = 'MD', comparison: other treatments vs 'Placebo'):

	MD	95%-CI	z	p-value
GLP-1	-5.2328	[-8.0763; -2.3893]	-3.61	0.0003
GLP-1 + Metformin	-5.5486	[-8.7694; -2.3278]	-3.38	0.0007
Metformin	-3.5877	[-6.8869; -0.2886]	-2.13	0.0331
Myoinositol + Folic acid	-3.6746	[-7.3358; -0.0135]	-1.97	0.0492
Placebo	.	.	.	.

Quantifying heterogeneity / inconsistency:
tau^2 = 0.5855; tau = 0.7651; I^2 = 17.9% [0.0%; 58.0%]

Tests of heterogeneity (within designs) and inconsistency (between designs):

	Q	d.f.	p-value
Total	12.18	10	0.2732
Within designs	12.17	9	0.2038
Between designs	0.01	1	0.9261

Details of network meta-analysis methods:
- Frequentist graph-theoretical approach
- DerSimonian-Laird estimator for tau^2
- Calculation of I^2

based on Q [[12]]

Original data:

	treat1	treat2	TE	seTE
Wen,2023	GLP-1 + Metformin	Metformin	-2.0700	0.8700
Xing,2022	GLP-1 + Metformin	Metformin	-4.0700	3.5600
Elkind-Hirsch,2022	GLP-1	Placebo	-5.2000	1.3900
Ma,2021	GLP-1 + Metformin	Metformin	-1.7100	0.8600
Soldat-Stanković,2022	Metformin	Myoinositol + Folic acid	0.8500	5.4800
Tao,2021	GLP-1	Metformin	-1.2200	3.0600
Zheng,2019	GLP-1	Metformin	-1.6500	1.1500
Shokrpour,2019	Metformin	Myoinositol + Folic acid	0.1000	0.3000
Frøssing,2018	GLP-1	Placebo	-5.4000	3.5000
Jensterle,2017	GLP-1	GLP-1 + Metformin	-2.7000	1.1900
Zahra,2016	Metformin	Placebo	-3.1500	7.8900
Zheng,2017	GLP-1	Metformin	-1.6500	4.1800
Jensterle,2016	GLP-1	GLP-1 + Metformin	2.4000	0.9200
Jensterle,2015	GLP-1	Metformin	-0.7000	9.1300

Number of treatment arms (by study):

	narms
Wen,2023	2
Xing,2022	2
Elkind-Hirsch,2022	2
Ma,2021	2
Soldat-Stanković,2022	2
Tao,2021	2
Zheng,2019	2
Shokrpour,2019	2
Frøssing,2018	2
Jensterle,2017	2
Zahra,2016	2
Zheng,2017	2
Jensterle,2016	2
Jensterle,2015	2

Results (random effects model):

	treat1	treat2	MD	95%-CI
Wen,2023	GLP-1 + Metformin	Metformin	-1.9635	[-3.3207; -0.6064]
Xing,2022	GLP-1 + Metformin	Metformin	-1.9635	[-3.3207; -0.6064]
Elkind-Hirsch,2022	GLP-1	Placebo	-5.2184	[-7.9936; -2.4431]
Ma,2021	GLP-1 + Metformin	Metformin	-1.9635	[-3.3207; -0.6064]
Soldat-Stanković,2022	Metformin	Myoinositol + Folic acid	0.1151	[-1.4226; 1.6528]
Tao,2021	GLP-1	Metformin	-1.6433	[-3.2860; -0.0007]

Zheng,2019 GLP-1 Metformin -1.6433 [-3.2860; -0.0007]
Shokrpour,2019 Metformin Myoinositol + Folic acid 0.1151 [-1.4226; 1.6528]
Frøssing,2018 GLP-1 Placebo -5.2184 [-7.9936; -2.4431]
Jensterle,2017 GLP-1 GLP-1 + Metformin 0.3202 [-1.1682; 1.8086]
Zahra,2016 Metformin Placebo -3.5750 [-6.7732; -0.3769]
Zheng,2017 GLP-1 Metformin -1.6433 [-3.2860; -0.0007]
Jensterle,2016 GLP-1 GLP-1 + Metformin 0.3202 [-1.1682; 1.8086]
Jensterle,2015 GLP-1 Metformin -1.6433 [-3.2860; -0.0007]

Number of studies: k = 14
Number of pairwise comparisons: m = 14
Number of treatments: n = 5
Number of designs: d = 6

Random effects model

Treatment estimate (sm = 'MD', comparison: other treatments vs 'Placebo'):

	MD	95%-CI	z	p-value
GLP-1	-5.2184	[-7.9936; -2.4431]	-3.69	0.0002
GLP-1 + Metformin	-5.5385	[-8.6721; -2.4049]	-3.46	0.0005
Metformin	-3.5750	[-6.7732; -0.3769]	-2.19	0.0285
Myoinositol + Folic acid	-3.6901	[-7.2387; -0.1415]	-2.04	0.0415
Placebo	.	.	.	.

Quantifying heterogeneity / inconsistency:
tau^2 = 0.5382; tau = 0.7336; I^2 = 16.7% [0.0%; 57.1%]

Tests of heterogeneity (within designs) and inconsistency (between designs):

	Q	d.f.	p-value
Total	12.01	10	0.2844
Within designs	12.00	8	0.1514
Between designs	0.01	2	0.9933

Details of network meta-analysis methods:
- Frequentist graph-theoretical approach
- DerSimonian-Laird estimator for tau^2
- Calculation of I^2 based on Q

[[13]]
Original data:

	treat1	treat2	TE	seTE
Wen,2023	GLP-1 + Metformin	Metformin	-2.0700	0.8700
Xing,2022	GLP-1 + Metformin	Metformin	-4.0700	3.5600

Elkind-Hirsch,2022 GLP-1 Placebo -5.2000 1.3900
Ma,2021 GLP-1 + Metformin Metformin -1.7100 0.8600
Soldat-Stanković,2022 Metformin Myoinositol + Folic acid 0.8500 5.4800
Tao,2021 GLP-1 Metformin -1.2200 3.0600
Zheng,2019 GLP-1 Metformin -1.6500 1.1500
Shokrpour,2019 Metformin Myoinositol + Folic acid 0.1000 0.3000
Frøssing,2018 GLP-1 Placebo -5.4000 3.5000
Jensterle,2017 GLP-1 GLP-1 + Metformin -2.7000 1.1900
Zahra,2016 Metformin Placebo -3.1500 7.8900
Ravn,2022 Metformin Myoinositol + Folic acid -3.8000 9.3300
Jensterle,2016 GLP-1 GLP-1 + Metformin 2.4000 0.9200
Jensterle,2015 GLP-1 Metformin -0.7000 9.1300

Number of treatment arms (by study):

	narms
Wen,2023	2
Xing,2022	2
Elkind-Hirsch,2022	2
Ma,2021	2
Soldat-Stanković,2022	2
Tao,2021	2
Zheng,2019	2
Shokrpour,2019	2
Frøssing,2018	2
Jensterle,2017	2
Zahra,2016	2
Ravn,2022	2
Jensterle,2016	2
Jensterle,2015	2

Results (random effects model):

	treat1	treat2	MD	95%-CI
Wen,2023	GLP-1 + Metformin	Metformin	-1.9628	[-3.3537; -0.5720]
Xing,2022	GLP-1 + Metformin	Metformin	-1.9628	[-3.3537; -0.5720]
Elkind-Hirsch,2022	GLP-1	Placebo	-5.2188	[-8.0204; -2.4173]
Ma,2021	GLP-1 + Metformin	Metformin	-1.9628	[-3.3537; -0.5720]
Tao,2021	GLP-1	Metformin	-1.6511	[-3.3530; 0.0509]
Zheng,2019	GLP-1	Metformin	-1.6511	[-3.3530; 0.0509]
Shokrpour,2019	Metformin	Myoinositol + Folic acid	0.0867	[-1.5103; 1.683
Frøssing,2018	GLP-1	Placebo	-5.2188	[-8.0204; -2.4173]
Jensterle,2017	GLP-1	GLP-1 + Metformin	0.3118	[-1.2137; 1.8372]
Zahra,2016	Metformin	Placebo	-3.5678	[-6.8169; -0.3186]
Soldat-Stanković,2022	Metformin	Myoinositol + Folic acid	0.0867	[-1.5103; 1.6838]

8]

```
Ravn,2022                 Metformin Myoinositol + Folic acid 0.0867 [-1.5103; 1.6838]
Jensterle,2016                 GLP-1      GLP-1 + Metformin 0.3118 [-1.2137; 1.8372]
Jensterle,2015                 GLP-1              Metformin -1.6511 [-3.3530; 0.0509]

Number of studies: k = 14
Number of pairwise comparisons: m = 14
Number of treatments: n = 5
Number of designs: d = 6

Random effects model

Treatment estimate (sm = 'MD', comparison: other treatments vs 'Placebo'):
                           MD            95%-CI     z p-value
GLP-1                   -5.2188 [-8.0204; -2.4173] -3.65 0.0003
GLP-1 + Metformin        -5.5306 [-8.7037; -2.3575] -3.42 0.0006
Metformin               -3.5678 [-6.8169; -0.3186] -2.15 0.0314
Myoinositol + Folic acid -3.6545 [-7.2749; -0.0340] -1.98 0.0479
Placebo                       .                .     .       .

Quantifying heterogeneity / inconsistency:
tau^2 = 0.5940; tau = 0.7707; I^2 = 17.9% [0.0%; 58.0%]

Tests of heterogeneity (within designs) and inconsistency (between designs):
                  Q d.f. p-value
Total           12.18  10 0.2730
Within designs 12.17     8 0.1437
Between designs 0.01     2 0.9937

Details of network meta-analysis methods:
- Frequentist graph-theoretical approach
- DerSimonian-Laird estimator for tau^2
- Calculation of I^2 based on Q

[[14]]
Original data:

                              treat1                 treat2      TE  seTE
Wen,2023              GLP-1 + Metformin              Metformin -2.0700 0.8700
Xing,2022             GLP-1 + Metformin              Metformin -4.0700 3.5600
Elkind-Hirsch,2022               GLP-1               Placebo -5.2000 1.3900
Ma,2021               GLP-1 + Metformin              Metformin -1.7100 0.8600
Soldat-Stanković,2022         Metformin Myoinositol + Folic acid 0.8500 5.4800
Tao,2021                       GLP-1              Metformin -1.2200 3.0600
Zheng,2019                       GLP-1              Metformin -1.6500 1.1500
```

Shokrpour,2019	Metformin	Myoinositol + Folic acid	0.1000	0.3000
Frøssing,2018	GLP-1	Placebo	-5.4000	3.5000
Jensterle,2017	GLP-1	GLP-1 + Metformin	-2.7000	1.1900
Zahra,2016	Metformin	Placebo	-3.1500	7.8900
Ravn,2022	Metformin	Myoinositol + Folic acid	-3.8000	9.3300
Zheng,2017	GLP-1	Metformin	-1.6500	4.1800
Jensterle,2015	GLP-1	Metformin	-0.7000	9.1300

Number of treatment arms (by study):

	narms
Wen,2023	2
Xing,2022	2
Elkind-Hirsch,2022	2
Ma,2021	2
Soldat-Stanković,2022	2
Tao,2021	2
Zheng,2019	2
Shokrpour,2019	2
Frøssing,2018	2
Jensterle,2017	2
Zahra,2016	2
Ravn,2022	2
Zheng,2017	2
Jensterle,2015	2

Results (random effects model):

	treat1	treat2	MD	95%-CI
Wen,2023	GLP-1 + Metformin	Metformin	-1.5590	[-2.6621; -0.4559]
Xing,2022	GLP-1 + Metformin	Metformin	-1.5590	[-2.6621; -0.4559]
Elkind-Hirsch,2022	GLP-1	Placebo	-5.2444	[-7.7434; -2.7453]
Ma,2021	GLP-1 + Metformin	Metformin	-1.5590	[-2.6621; -0.4559]
Soldat-Stanković,2022	Metformin	Myoinositol + Folic acid	0.0982	[-0.4886; 0.6850]
Tao,2021	GLP-1	Metformin	-2.7333	[-4.3284; -1.1382]
Zheng,2019	GLP-1	Metformin	-2.7333	[-4.3284; -1.1382]
Shokrpour,2019	Metformin	Myoinositol + Folic acid	0.0982	[-0.4886; 0.6850]
Frøssing,2018	GLP-1	Placebo	-5.2444	[-7.7434; -2.7453]
Jensterle,2017	GLP-1	GLP-1 + Metformin	-1.1743	[-2.8242; 0.4756]
Zahra,2016	Metformin	Placebo	-2.5111	[-5.4533; 0.4312]
Ravn,2022	Metformin	Myoinositol + Folic acid	0.0982	[-0.4886; 0.6850]
Zheng,2017	GLP-1	Metformin	-2.7333	[-4.3284; -1.1382]
Jensterle,2015	GLP-1	Metformin	-2.7333	[-4.3284; -1.1382]

Number of studies: k = 14

Number of pairwise comparisons: m = 14
Number of treatments: n = 5
Number of designs: d = 6

Random effects model

Treatment estimate (sm = 'MD', comparison: other treatments vs 'Placebo'):

	MD	95%-CI	z	p-value
GLP-1	-5.2444	[-7.7434; -2.7453]	-4.11	< 0.0001
GLP-1 + Metformin	-4.0701	[-7.0469; -1.0932]	-2.68	0.0074
Metformin	-2.5111	[-5.4533; 0.4312]	-1.67	0.0944
Myoinositol + Folic acid	-2.6093	[-5.6095; 0.3909]	-1.70	0.0883
Placebo	.	.	.	.

Quantifying heterogeneity / inconsistency:
tau^2 = 0; tau = 0; I^2 = 0% [0.0%; 60.2%]

Tests of heterogeneity (within designs) and inconsistency (between designs):

	Q	d.f.	p-value
Total	3.97	10	0.9487
Within designs	0.67	8	0.9996
Between designs	3.29	2	0.1926

Details of network meta-analysis methods:
- Frequentist graph-theoretical approach
- DerSimonian-Laird estimator for tau^2
- Calculation of I^2 based on Q

[[15]]
Original data:

	treat1	treat2	TE	seTE
Wen,2023	GLP-1 + Metformin	Metformin	-2.0700	0.8700
Xing,2022	GLP-1 + Metformin	Metformin	-4.0700	3.5600
Elkind-Hirsch,2022	GLP-1	Placebo	-5.2000	1.3900
Ma,2021	GLP-1 + Metformin	Metformin	-1.7100	0.8600
Soldat-Stanković,2022	Metformin	Myoinositol + Folic acid	0.8500	5.4800
Tao,2021	GLP-1	Metformin	-1.2200	3.0600
Zheng,2019	GLP-1	Metformin	-1.6500	1.1500
Shokrpour,2019	Metformin	Myoinositol + Folic acid	0.1000	0.3000
Frøssing,2018	GLP-1	Placebo	-5.4000	3.5000
Jensterle,2017	GLP-1	GLP-1 + Metformin	-2.7000	1.1900
Zahra,2016	Metformin	Placebo	-3.1500	7.8900
Ravn,2022	Metformin	Myoinositol + Folic acid	-3.8000	9.3300

Zheng,2017	GLP-1	Metformin	-1.6500	4.1800
Jensterle,2016	GLP-1	GLP-1 + Metformin	2.4000	0.9200

Number of treatment arms (by study):

	narms
Wen,2023	2
Xing,2022	2
Elkind-Hirsch,2022	2
Ma,2021	2
Soldat-Stanković,2022	2
Tao,2021	2
Zheng,2019	2
Shokrpour,2019	2
Frøssing,2018	2
Jensterle,2017	2
Zahra,2016	2
Ravn,2022	2
Zheng,2017	2
Jensterle,2016	2

Results (random effects model):

	treat1	treat2	MD	95%-CI
Wen,2023	GLP-1 + Metformin	Metformin	-1.9665	[-3.3456; -0.5874]
Xing,2022	GLP-1 + Metformin	Metformin	-1.9665	[-3.3456; -0.5874]
Elkind-Hirsch,2022	GLP-1	Placebo	-5.2190	[-8.0149; -2.4231]
Ma,2021	GLP-1 + Metformin	Metformin	-1.9665	[-3.3456; -0.5874]
Soldat-Stanković,2022	Metformin	Myoinositol + Folic acid	0.0869	[-1.4964; 1.6702]
Tao,2021	GLP-1	Metformin	-1.6576	[-3.3270; 0.0117]
Zheng,2019	GLP-1	Metformin	-1.6576	[-3.3270; 0.0117]
Shokrpour,2019	Metformin	Myoinositol + Folic acid	0.0869	[-1.4964; 1.6702]
Frøssing,2018	GLP-1	Placebo	-5.2190	[-8.0149; -2.4231]
Jensterle,2017	GLP-1	GLP-1 + Metformin	0.3089	[-1.2015; 1.8193]
Zahra,2016	Metformin	Placebo	-3.5614	[-6.7899; -0.3329]
Ravn,2022	Metformin	Myoinositol + Folic acid	0.0869	[-1.4964; 1.6702]
Zheng,2017	GLP-1	Metformin	-1.6576	[-3.3270; 0.0117]
Jensterle,2016	GLP-1	GLP-1 + Metformin	0.3089	[-1.2015; 1.8193]

Number of studies: k = 14
Number of pairwise comparisons: m = 14
Number of treatments: n = 5
Number of designs: d = 6

Random effects model

Treatment estimate (sm = 'MD', comparison: other treatments vs 'Placebo'):

	MD	95%-CI	z	p-value
GLP-1	-5.2190	[-8.0149; -2.4231]	-3.66	0.0003
GLP-1 + Metformin	-5.5279	[-8.6895; -2.3663]	-3.43	0.0006
Metformin	-3.5614	[-6.7899; -0.3329]	-2.16	0.0306
Myoinositol + Folic acid	-3.6483	[-7.2441; -0.0525]	-1.99	0.0467
Placebo	.	.	.	.

Quantifying heterogeneity / inconsistency:
tau^2 = 0.5819; tau = 0.7628; I^2 = 17.9% [0.0%; 58.0%]

Tests of heterogeneity (within designs) and inconsistency (between designs):

	Q	d.f.	p-value
Total	12.18	10	0.2734
Within designs	12.16	8	0.1441
Between designs	0.01	2	0.9926

Details of network meta-analysis methods:
- Frequentist graph-theoretical approach
- DerSimonian-Laird estimator for tau^2
- Calculation of I^2 based on Q

=== Heterogeneity & Inconsistency ===
[1] 0.09720893
Q statistics to assess homogeneity / consistency

Design-specific decomposition of within-designs Q statistic

Design	Q	df	p-value
GLP-1 vs GLP-1 + Metformin	11.50	1	0.0007
GLP-1 + Metformin vs Metformin	0.45	2	0.7979
Metformin vs Myoinositol + Folic acid	0.19	2	0.9078
Placebo vs GLP-1	0.00	1	0.9576
GLP-1 vs Metformin	0.03	3	0.9988

Between-designs Q statistic after detaching of single designs
(influential designs have p-value markedly different from 0.9933)

Detached design	Q	df	p-value
GLP-1 vs GLP-1 + Metformin	0.00	1	0.9517
GLP-1 + Metformin vs Metformin	0.00	1	0.9517

GLP-1 vs Metformin 0.01 1 0.9387
Placebo vs GLP-1 0.01 1 0.9261
Placebo vs Metformin 0.01 1 0.9261

Q statistic to assess consistency under the assumption of
a full design-by-treatment interaction random effects model

BMI Change

== Sensitivity Analysis Results ===

=== Excluding High Risk of Bias (rob != 3) ===
❌ Not enough data to run network meta-analysis for low-risk studies.

=== Leave-One-Out Analysis ===
[[1]]
Original data:

	treat1	treat2	TE	seTE
Xing,2022	GLP-1 + Metformin	Metformin	-1.5300	1.2700
Elkind-Hirsch,2022	GLP-1	Placebo	-2.0000	0.5100
Ma,2021	GLP-1 + Metformin	Metformin	-0.6300	0.3200
Soldat-Stanković,2022	Metformin	Myoinositol + Folic acid	0.1900	1.6500
Tao,2021	GLP-1	Metformin	-0.1900	1.0200
Zheng,2019	GLP-1	Metformin	-0.7600	0.6900
Shokrpour,2019	Metformin	Myoinositol + Folic acid	0.1000	0.1200
Frøssing,2018	GLP-1	Placebo	-2.0000	0.0790
Jensterle,2017	GLP-1	GLP-1 + Metformin	-0.9000	0.4200
Zahra,2016	Metformin	Placebo	-1.5000	3.2700
Ravn,2022	Metformin	Myoinositol + Folic acid	-1.2600	3.1700
Zheng,2017	GLP-1	Metformin	-0.7600	1.5000
Jensterle,2016	GLP-1	GLP-1 + Metformin	0.8000	0.3100
Jensterle,2015	GLP-1	Metformin	-0.2000	2.7800

Number of treatment arms (by study):

	narms
Xing,2022	2
Elkind-Hirsch,2022	2
Ma,2021	2
Soldat-Stanković,2022	2

Tao,2021	2
Zheng,2019	2
Shokrpour,2019	2
Frøssing,2018	2
Jensterle,2017	2
Zahra,2016	2
Ravn,2022	2
Zheng,2017	2
Jensterle,2016	2
Jensterle,2015	2

Results (random effects model):

	treat1	treat2	MD	95%-CI
Xing,2022	GLP-1 + Metformin	Metformin	-0.7084	[-1.3574; -0.0594]
Elkind-Hirsch,2022	GLP-1	Placebo	-2.0005	[-2.4737; -1.5272]
Ma,2021	GLP-1 + Metformin	Metformin	-0.7084	[-1.3574; -0.0594]
Soldat-Stanković,2022	Metformin	Myoinositol + Folic acid	0.0921	[-0.4512; 0.6355]
Tao,2021	GLP-1	Metformin	-0.5869	[-1.3065; 0.1328]
Zheng,2019	GLP-1	Metformin	-0.5869	[-1.3065; 0.1328]
Shokrpour,2019	Metformin	Myoinositol + Folic acid	0.0921	[-0.4512; 0.6355]
Frøssing,2018	GLP-1	Placebo	-2.0005	[-2.4737; -1.5272]
Jensterle,2017	GLP-1	GLP-1 + Metformin	0.1215	[-0.4312; 0.6742]
Zahra,2016	Metformin	Placebo	-1.4136	[-2.2716; -0.5555]
Ravn,2022	Metformin	Myoinositol + Folic acid	0.0921	[-0.4512; 0.6355]
Zheng,2017	GLP-1	Metformin	-0.5869	[-1.3065; 0.1328]
Jensterle,2016	GLP-1	GLP-1 + Metformin	0.1215	[-0.4312; 0.6742]
Jensterle,2015	GLP-1	Metformin	-0.5869	[-1.3065; 0.1328]

Number of studies: k = 14
Number of pairwise comparisons: m = 14
Number of treatments: n = 5
Number of designs: d = 6

Random effects model

Treatment estimate (sm = 'MD', comparison: other treatments vs 'Placebo'):

	MD	95%-CI	z	p-value
GLP-1	-2.0005	[-2.4737; -1.5272]	-8.28	< 0.0001
GLP-1 + Metformin	-2.1220	[-2.8481; -1.3958]	-5.73	< 0.0001
Metformin	-1.4136	[-2.2716; -0.5555]	-3.23	0.0012
Myoinositol + Folic acid	-1.5057	[-2.5214; -0.4901]	-2.91	0.0037
Placebo	.	.	.	.

Quantifying heterogeneity / inconsistency:
tau^2 = 0.0653; tau = 0.2555; I^2 = 13.3% [0.0%; 53.9%]

Tests of heterogeneity (within designs) and inconsistency (between designs):

	Q	d.f.	p-value
Total	11.54	10	0.3172
Within designs	11.51	8	0.1744
Between designs	0.03	2	0.9871

Details of network meta-analysis methods:
- Frequentist graph-theoretical approach
- DerSimonian-Laird estimator for tau^2
- Calculation of I^2 based on Q

[[2]]
Original data:

	treat1	treat2	TE	seTE
Wen,2023	GLP-1 + Metformin	Metformin	-0.9500	0.3100
Elkind-Hirsch,2022	GLP-1	Placebo	-2.0000	0.5100
Ma,2021	GLP-1 + Metformin	Metformin	-0.6300	0.3200
Soldat-Stanković,2022	Metformin	Myoinositol + Folic acid	0.1900	1.6500
Tao,2021	GLP-1	Metformin	-0.1900	1.0200
Zheng,2019	GLP-1	Metformin	-0.7600	0.6900
Shokrpour,2019	Metformin	Myoinositol + Folic acid	0.1000	0.1200
Frøssing,2018	GLP-1	Placebo	-2.0000	0.0790
Jensterle,2017	GLP-1	GLP-1 + Metformin	-0.9000	0.4200
Zahra,2016	Metformin	Placebo	-1.5000	3.2700
Ravn,2022	Metformin	Myoinositol + Folic acid	-1.2600	3.1700
Zheng,2017	GLP-1	Metformin	-0.7600	1.5000
Jensterle,2016	GLP-1	GLP-1 + Metformin	0.8000	0.3100
Jensterle,2015	GLP-1	Metformin	-0.2000	2.7800

Number of treatment arms (by study):

	narms
Wen,2023	2
Elkind-Hirsch,2022	2
Ma,2021	2
Soldat-Stanković,2022	2
Tao,2021	2
Zheng,2019	2
Shokrpour,2019	2
Frøssing,2018	2
Jensterle,2017	2

Zahra,2016 2
Ravn,2022 2
Zheng,2017 2
Jensterle,2016 2
Jensterle,2015 2

Results (random effects model):

	treat1	treat2	MD	95%-CI
Wen,2023	GLP-1 + Metformin	Metformin	-0.7808	[-1.2679; -0.2937]
Elkind-Hirsch,2022	GLP-1	Placebo	-2.0006	[-2.4228; -1.5783]
Ma,2021	GLP-1 + Metformin	Metformin	-0.7808	[-1.2679; -0.2937]
Soldat-Stanković,2022	Metformin	Myoinositol + Folic acid	0.0937	[-0.3915; 0.5789]
Tao,2021	GLP-1	Metformin	-0.6311	[-1.2620; -0.0003]
Zheng,2019	GLP-1	Metformin	-0.6311	[-1.2620; -0.0003]
Shokrpour,2019	Metformin	Myoinositol + Folic acid	0.0937	[-0.3915; 0.5789]
Frøssing,2018	GLP-1	Placebo	-2.0006	[-2.4228; -1.5783]
Jensterle,2017	GLP-1	GLP-1 + Metformin	0.1496	[-0.3720; 0.6712]
Zahra,2016	Metformin	Placebo	-1.3694	[-2.1263; -0.6126]
Ravn,2022	Metformin	Myoinositol + Folic acid	0.0937	[-0.3915; 0.5789]
Zheng,2017	GLP-1	Metformin	-0.6311	[-1.2620; -0.0003]
Jensterle,2016	GLP-1	GLP-1 + Metformin	0.1496	[-0.3720; 0.6712]
Jensterle,2015	GLP-1	Metformin	-0.6311	[-1.2620; -0.0003]

Number of studies: k = 14
Number of pairwise comparisons: m = 14
Number of treatments: n = 5
Number of designs: d = 6

Random effects model

Treatment estimate (sm = 'MD', comparison: other treatments vs 'Placebo'):

	MD	95%-CI	z	p-value
GLP-1	-2.0006	[-2.4228; -1.5783]	-9.29	< 0.0001
GLP-1 + Metformin	-2.1502	[-2.8199; -1.4805]	-6.29	< 0.0001
Metformin	-1.3694	[-2.1263; -0.6126]	-3.55	0.0004
Myoinositol + Folic acid	-1.4632	[-2.3622; -0.5641]	-3.19	0.0014
Placebo	.	.	.	.

Quantifying heterogeneity / inconsistency:
tau^2 = 0.0487; tau = 0.2206; I^2 = 13.5% [0.0%; 54.0%]

Tests of heterogeneity (within designs) and inconsistency (between designs):
Q d.f. p-value

Total 11.56 10 0.3159
Within designs 11.56 8 0.1722
Between designs 0.00 2 0.9996

Details of network meta-analysis methods:
- Frequentist graph-theoretical approach
- DerSimonian-Laird estimator for tau^2
- Calculation of I^2 based on Q

[[3]]
Original data:

	treat1	treat2	TE	seTE
Wen,2023	GLP-1 + Metformin	Metformin	-0.9500	0.3100
Xing,2022	GLP-1 + Metformin	Metformin	-1.5300	1.2700
Ma,2021	GLP-1 + Metformin	Metformin	-0.6300	0.3200
Soldat-Stanković,2022	Metformin	Myoinositol + Folic acid	0.1900	1.6500
Tao,2021	GLP-1	Metformin	-0.1900	1.0200
Zheng,2019	GLP-1	Metformin	-0.7600	0.6900
Shokrpour,2019	Metformin	Myoinositol + Folic acid	0.1000	0.1200
Frøssing,2018	GLP-1	Placebo	-2.0000	0.0790
Jensterle,2017	GLP-1	GLP-1 + Metformin	-0.9000	0.4200
Zahra,2016	Metformin	Placebo	-1.5000	3.2700
Ravn,2022	Metformin	Myoinositol + Folic acid	-1.2600	3.1700
Zheng,2017	GLP-1	Metformin	-0.7600	1.5000
Jensterle,2016	GLP-1	GLP-1 + Metformin	0.8000	0.3100
Jensterle,2015	GLP-1	Metformin	-0.2000	2.7800

Number of treatment arms (by study):

	narms
Wen,2023	2
Xing,2022	2
Ma,2021	2
Soldat-Stanković,2022	2
Tao,2021	2
Zheng,2019	2
Shokrpour,2019	2
Frøssing,2018	2
Jensterle,2017	2
Zahra,2016	2
Ravn,2022	2
Zheng,2017	2
Jensterle,2016	2
Jensterle,2015	2

Results (random effects model):

	treat1	treat2	MD	95%-CI
Wen,2023	GLP-1 + Metformin	Metformin	-0.8054	[-1.3179; -0.2929]
Xing,2022	GLP-1 + Metformin	Metformin	-0.8054	[-1.3179; -0.2929]
Ma,2021	GLP-1 + Metformin	Metformin	-0.8054	[-1.3179; -0.2929]
Soldat-Stanković,2022	Metformin	Myoinositol + Folic acid	0.0913	[-0.4812; 0.6638]
Tao,2021	GLP-1	Metformin	-0.6644	[-1.3252; -0.0036]
Zheng,2019	GLP-1	Metformin	-0.6644	[-1.3252; -0.0036]
Shokrpour,2019	Metformin	Myoinositol + Folic acid	0.0913	[-0.4812; 0.6638]
Frøssing,2018	GLP-1	Placebo	-2.0012	[-2.5557; -1.4467]
Jensterle,2017	GLP-1	GLP-1 + Metformin	0.1410	[-0.4137; 0.6957]
Zahra,2016	Metformin	Placebo	-1.3368	[-2.1956; -0.4780]
Ravn,2022	Metformin	Myoinositol + Folic acid	0.0913	[-0.4812; 0.6638]
Zheng,2017	GLP-1	Metformin	-0.6644	[-1.3252; -0.0036]
Jensterle,2016	GLP-1	GLP-1 + Metformin	0.1410	[-0.4137; 0.6957]
Jensterle,2015	GLP-1	Metformin	-0.6644	[-1.3252; -0.0036]

Number of studies: k = 14
Number of pairwise comparisons: m = 14
Number of treatments: n = 5
Number of designs: d = 6

Random effects model

Treatment estimate (sm = 'MD', comparison: other treatments vs 'Placebo'):

	MD	95%-CI	z	p-value
GLP-1	-2.0012	[-2.5557; -1.4467]	-7.07	< 0.0001
GLP-1 + Metformin	-2.1422	[-2.9242; -1.3602]	-5.37	< 0.0001
Metformin	-1.3368	[-2.1956; -0.4780]	-3.05	0.0023
Myoinositol + Folic acid	-1.4281	[-2.4603; -0.3959]	-2.71	0.0067
Placebo	.	.	.	.

Quantifying heterogeneity / inconsistency:
tau^2 = 0.0744; tau = 0.2727; I^2 = 15.8% [0.0%; 56.3%]

Tests of heterogeneity (within designs) and inconsistency (between designs):

	Q	d.f.	p-value
Total	11.88	10	0.2930
Within designs	11.88	8	0.1566
Between designs	0.00	2	0.9985

Details of network meta-analysis methods:

- Frequentist graph-theoretical approach
- DerSimonian-Laird estimator for tau^2
- Calculation of I^2 based on Q

```
[[4]]
Original data:

                          treat1                 treat2      TE  seTE
Wen,2023             GLP-1 + Metformin              Metformin -0.9500 0.3100
Xing,2022            GLP-1 + Metformin              Metformin -1.5300 1.2700
Elkind-Hirsch,2022               GLP-1                Placebo -2.0000 0.5100
Soldat-Stanković,2022        Metformin Myoinositol + Folic acid 0.1900 1.6500
Tao,2021                         GLP-1              Metformin -0.1900 1.0200
Zheng,2019                       GLP-1              Metformin -0.7600 0.6900
Shokrpour,2019               Metformin Myoinositol + Folic acid 0.1000 0.1200
Frøssing,2018                    GLP-1                Placebo -2.0000 0.0790
Jensterle,2017                   GLP-1      GLP-1 + Metformin -0.9000 0.4200
Zahra,2016                   Metformin                Placebo -1.5000 3.2700
Ravn,2022                    Metformin Myoinositol + Folic acid -1.2600 3.1700
Zheng,2017                       GLP-1              Metformin -0.7600 1.5000
Jensterle,2016                   GLP-1      GLP-1 + Metformin 0.8000 0.3100
Jensterle,2015                   GLP-1              Metformin -0.2000 2.7800

Number of treatment arms (by study):
                  narms
Wen,2023              2
Xing,2022             2
Elkind-Hirsch,2022    2
Soldat-Stanković,2022 2
Tao,2021              2
Zheng,2019            2
Shokrpour,2019        2
Frøssing,2018         2
Jensterle,2017        2
Zahra,2016            2
Ravn,2022             2
Zheng,2017            2
Jensterle,2016        2
Jensterle,2015        2

Results (random effects model):

                          treat1                 treat2      MD            95%-CI
Wen,2023             GLP-1 + Metformin              Metformin -0.9210 [-1.5476; -0.2944]
```

Xing,2022 GLP-1 + Metformin Metformin -0.9210 [-1.5476; -0.2944]
Elkind-Hirsch,2022 GLP-1 Placebo -2.0012 [-2.4475; -1.5548]
Soldat-Stanković,2022 Metformin Myoinositol + Folic acid 0.0930 [-0.4194; 0.6054]
Tao,2021 GLP-1 Metformin -0.7425 [-1.4459; -0.0391]
Zheng,2019 GLP-1 Metformin -0.7425 [-1.4459; -0.0391]
Shokrpour,2019 Metformin Myoinositol + Folic acid 0.0930 [-0.4194; 0.6054]
Frøssing,2018 GLP-1 Placebo -2.0012 [-2.4475; -1.5548]
Jensterle,2017 GLP-1 GLP-1 + Metformin 0.1785 [-0.3611; 0.7181]
Zahra,2016 Metformin Placebo -1.2587 [-2.0889; -0.4285]
Ravn,2022 Metformin Myoinositol + Folic acid 0.0930 [-0.4194; 0.6054]
Zheng,2017 GLP-1 Metformin -0.7425 [-1.4459; -0.0391]
Jensterle,2016 GLP-1 GLP-1 + Metformin 0.1785 [-0.3611; 0.7181]
Jensterle,2015 GLP-1 Metformin -0.7425 [-1.4459; -0.0391]

Number of studies: k = 14
Number of pairwise comparisons: m = 14
Number of treatments: n = 5
Number of designs: d = 6

Random effects model

Treatment estimate (sm = 'MD', comparison: other treatments vs 'Placebo'):

	MD	95%-CI	z	p-value
GLP-1	-2.0012	[-2.4475; -1.5548]	-8.79	< 0.0001
GLP-1 + Metformin	-2.1797	[-2.8786; -1.4808]	-6.11	< 0.0001
Metformin	-1.2587	[-2.0889; -0.4285]	-2.97	0.0030
Myoinositol + Folic acid	-1.3517	[-2.3273; -0.3761]	-2.72	0.0066
Placebo	.	.	.	.

Quantifying heterogeneity / inconsistency:
tau^2 = 0.0562; tau = 0.2370; I^2 = 11.7% [0.0%; 52.0%]

Tests of heterogeneity (within designs) and inconsistency (between designs):

	Q	d.f.	p-value
Total	11.33	10	0.3326
Within designs	11.24	8	0.1887
Between designs	0.09	2	0.9555

Details of network meta-analysis methods:
- Frequentist graph-theoretical approach
- DerSimonian-Laird estimator for tau^2
- Calculation of I^2

based on Q [[5]]

Original data:

	treat1	treat2	TE	seTE
Wen,2023	GLP-1 + Metformin	Metformin	-0.9500	0.3100
Xing,2022	GLP-1 + Metformin	Metformin	-1.5300	1.2700
Elkind-Hirsch,2022	GLP-1	Placebo	-2.0000	0.5100
Ma,2021	GLP-1 + Metformin	Metformin	-0.6300	0.3200
Tao,2021	GLP-1	Metformin	-0.1900	1.0200
Zheng,2019	GLP-1	Metformin	-0.7600	0.6900
Shokrpour,2019	Metformin	Myoinositol + Folic acid	0.1000	0.1200
Frøssing,2018	GLP-1	Placebo	-2.0000	0.0790
Jensterle,2017	GLP-1	GLP-1 + Metformin	-0.9000	0.4200
Zahra,2016	Metformin	Placebo	-1.5000	3.2700
Ravn,2022	Metformin	Myoinositol + Folic acid	-1.2600	3.1700
Zheng,2017	GLP-1	Metformin	-0.7600	1.5000
Jensterle,2016	GLP-1	GLP-1 + Metformin	0.8000	0.3100
Jensterle,2015	GLP-1	Metformin	-0.2000	2.7800

Number of treatment arms (by study):

	narms
Wen,2023	2
Xing,2022	2
Elkind-Hirsch,2022	2
Ma,2021	2
Tao,2021	2
Zheng,2019	2
Shokrpour,2019	2
Frøssing,2018	2
Jensterle,2017	2
Zahra,2016	2
Ravn,2022	2
Zheng,2017	2
Jensterle,2016	2
Jensterle,2015	2

Results (random effects model):

	treat1	treat2	MD	95%-CI
Wen,2023	GLP-1 + Metformin	Metformin	-0.8067	[-1.2986; -0.3148]
Xing,2022	GLP-1 + Metformin	Metformin	-0.8067	[-1.2986; -0.3148]
Elkind-Hirsch,2022	GLP-1	Placebo	-2.0008	[-2.4546; -1.5470]
Ma,2021	GLP-1 + Metformin	Metformin	-0.8067	[-1.2986; -0.3148]
Tao,2021	GLP-1	Metformin	-0.6572	[-1.2976; -0.0167]
Zheng,2019	GLP-1	Metformin	-0.6572	[-1.2976; -0.0167]

Shokrpour,2019	Metformin	Myoinositol + Folic acid	0.0902	[-0.4373; 0.6178]
Frøssing,2018	GLP-1	Placebo	-2.0008	[-2.4546; -1.5470]
Jensterle,2017	GLP-1	GLP-1 + Metformin	0.1495	[-0.3849; 0.6840]
Zahra,2016	Metformin	Placebo	-1.3436	[-2.1259; -0.5613]
Ravn,2022	Metformin	Myoinositol + Folic acid	0.0902	[-0.4373; 0.6178]
Zheng,2017	GLP-1	Metformin	-0.6572	[-1.2976; -0.0167]
Jensterle,2016	GLP-1	GLP-1 + Metformin	0.1495	[-0.3849; 0.6840]
Jensterle,2015	GLP-1	Metformin	-0.6572	[-1.2976; -0.0167]

Number of studies: k = 14
Number of pairwise comparisons: m = 14
Number of treatments: n = 5
Number of designs: d = 6

Random effects model

Treatment estimate (sm = 'MD', comparison: other treatments vs 'Placebo'):

	MD	95%-CI	z	p-value
GLP-1	-2.0008	[-2.4546; -1.5470]	-8.64	< 0.0001
GLP-1 + Metformin	-2.1503	[-2.8498; -1.4508]	-6.03	< 0.0001
Metformin	-1.3436	[-2.1259; -0.5613]	-3.37	0.0008
Myoinositol + Folic acid	-1.4339	[-2.3775; -0.4903]	-2.98	0.0029
Placebo	.	.	.	.

Quantifying heterogeneity / inconsistency:
tau^2 = 0.0586; tau = 0.2420; I^2 = 15.8% [0.0%; 56.3%]

Tests of heterogeneity (within designs) and inconsistency (between designs):

	Q	d.f.	p-value
Total	11.88	10	0.2932
Within designs	11.88	8	0.1568
Between designs	0.00	2	0.9985

Details of network meta-analysis methods:
- Frequentist graph-theoretical approach
- DerSimonian-Laird estimator for tau^2
- Calculation of I^2 based on Q

[[6]]
Original data:

	treat1	treat2	TE	seTE
Wen,2023	GLP-1 + Metformin	Metformin	-0.9500	0.3100
Xing,2022	GLP-1 + Metformin	Metformin	-1.5300	1.2700

Elkind-Hirsch,2022	GLP-1	Placebo	-2.0000	0.5100
Ma,2021	GLP-1 + Metformin	Metformin	-0.6300	0.3200
Soldat-Stanković,2022	Metformin	Myoinositol + Folic acid	0.1900	1.6500
Zheng,2019	GLP-1	Metformin	-0.7600	0.6900
Shokrpour,2019	Metformin	Myoinositol + Folic acid	0.1000	0.1200
Frøssing,2018	GLP-1	Placebo	-2.0000	0.0790
Jensterle,2017	GLP-1	GLP-1 + Metformin	-0.9000	0.4200
Zahra,2016	Metformin	Placebo	-1.5000	3.2700
Ravn,2022	Metformin	Myoinositol + Folic acid	-1.2600	3.1700
Zheng,2017	GLP-1	Metformin	-0.7600	1.5000
Jensterle,2016	GLP-1	GLP-1 + Metformin	0.8000	0.3100
Jensterle,2015	GLP-1	Metformin	-0.2000	2.7800

Number of treatment arms (by study):

	narms
Wen,2023	2
Xing,2022	2
Elkind-Hirsch,2022	2
Ma,2021	2
Soldat-Stanković,2022	2
Zheng,2019	2
Shokrpour,2019	2
Frøssing,2018	2
Jensterle,2017	2
Zahra,2016	2
Ravn,2022	2
Zheng,2017	2
Jensterle,2016	2
Jensterle,2015	2

Results (random effects model):

	treat1	treat2	MD	95%-CI
Wen,2023	GLP-1 + Metformin	Metformin	-0.8291	[-1.3237; -0.3344]
Xing,2022	GLP-1 + Metformin	Metformin	-0.8291	[-1.3237; -0.3344]
Elkind-Hirsch,2022	GLP-1	Placebo	-2.0010	[-2.4413; -1.5606]
Ma,2021	GLP-1 + Metformin	Metformin	-0.8291	[-1.3237; -0.3344]
Soldat-Stanković,2022	Metformin	Myoinositol + Folic acid	0.0932	[-0.4124; 0.5988]
Zheng,2019	GLP-1	Metformin	-0.7041	[-1.3715; -0.0367]
Shokrpour,2019	Metformin	Myoinositol + Folic acid	0.0932	[-0.4124; 0.5988]
Frøssing,2018	GLP-1	Placebo	-2.0010	[-2.4413; -1.5606]
Jensterle,2017	GLP-1	GLP-1 + Metformin	0.1250	[-0.4159; 0.6659]
Zahra,2016	Metformin	Placebo	-1.2969	[-2.0938; -0.4999]
Ravn,2022	Metformin	Myoinositol + Folic acid	0.0932	[-0.4124; 0.5988]

```
Zheng,2017                  GLP-1             Metformin -0.7041 [-1.3715; -0.0367]
Jensterle,2016              GLP-1     GLP-1 + Metformin 0.1250 [-0.4159; 0.6659]
Jensterle,2015              GLP-1             Metformin -0.7041 [-1.3715; -0.0367]

Number of studies: k = 14
Number of pairwise comparisons: m = 14
Number of treatments: n = 5
Number of designs: d = 6

Random effects model

Treatment estimate (sm = 'MD', comparison: other treatments vs 'Placebo'):
                          MD           95%-CI     z p-value
GLP-1                    -2.0010 [-2.4413; -1.5606] -8.91 < 0.0001
GLP-1 + Metformin        -2.1259 [-2.8218; -1.4301] -5.99 < 0.0001
Metformin                -1.2969 [-2.0938; -0.4999] -3.19 0.0014
Myoinositol + Folic acid -1.3901 [-2.3339; -0.4463] -2.89 0.0039
Placebo                        .                  .     .      .

Quantifying heterogeneity / inconsistency:
tau^2 = 0.0542; tau = 0.2329; I^2 = 14.5% [0.0%; 55.1%]

Tests of heterogeneity (within designs) and inconsistency (between designs):
                    Q d.f. p-value
Total           11.70 10 0.3055
Within designs 11.67     8 0.1665
Between designs 0.03     2 0.9852

Details of network meta-analysis methods:
- Frequentist graph-theoretical approach
- DerSimonian-Laird estimator for tau^2
- Calculation of I^2 based on Q

[[7]]
Original data:

                          treat1                 treat2      TE  seTE
Wen,2023              GLP-1 + Metformin              Metformin -0.9500 0.3100
Xing,2022             GLP-1 + Metformin              Metformin -1.5300 1.2700
Elkind-Hirsch,2022               GLP-1               Placebo -2.0000 0.5100
Ma,2021             GLP-1 + Metformin              Metformin -0.6300 0.3200
Soldat-Stanković,2022         Metformin Myoinositol + Folic acid 0.1900 1.6500
Tao,2021                      GLP-1             Metformin -0.1900 1.0200
Shokrpour,2019              Metformin Myoinositol + Folic acid 0.1000 0.1200
```

Frøssing,2018	GLP-1	Placebo	-2.0000	0.0790
Jensterle,2017	GLP-1	GLP-1 + Metformin	-0.9000	0.4200
Zahra,2016	Metformin	Placebo	-1.5000	3.2700
Ravn,2022	Metformin	Myoinositol + Folic acid	-1.2600	3.1700
Zheng,2017	GLP-1	Metformin	-0.7600	1.5000
Jensterle,2016	GLP-1	GLP-1 + Metformin	0.8000	0.3100
Jensterle,2015	GLP-1	Metformin	-0.2000	2.7800

Number of treatment arms (by study):

Wen,2023	2
Xing,2022	2
Elkind-Hirsch,2022	2
Ma,2021	2
Soldat-Stanković,2022	
Tao,2021	2
Shokrpour,2019	2
Frøssing,2018	2
Jensterle,2017	2
Zahra,2016	2
Ravn,2022	2
Zheng,2017	2
Jensterle,2016	2
Jensterle,2015	2

narms

2

Results (random effects model):

	treat1	treat2	MD	95%-CI
Wen,2023	GLP-1 + Metformin	Metformin	-0.7951	[-1.3112; -0.2789]
Xing,2022	GLP-1 + Metformin	Metformin	-0.7951	[-1.3112; -0.2789]
Elkind-Hirsch,2022	GLP-1	Placebo	-2.0007	[-2.4626; -1.5388]
Ma,2021	GLP-1 + Metformin	Metformin	-0.7951	[-1.3112; -0.2789]
Soldat-Stanković,2022	Metformin	Myoinositol + Folic acid	0.0925	[-0.4377; 0.6227]

Tao,2021	GLP-1	Metformin	-0.6330 [-1.3535; 0.0875]
Shokrpour,2019	Metformin	Myoinositol + Folic acid	0.0925 [-0.4377; 0.6227]
Frøssing,2018	GLP-1	Placebo	-2.0007 [-2.4626; -1.5388]
Jensterle,2017	GLP-1	GLP-1 + Metformin	0.1620 [-0.4048; 0.7289]
Zahra,2016	Metformin	Placebo	-1.3677 [-2.2204; -0.5150]
Ravn,2022	Metformin	Myoinositol + Folic acid	0.0925 [-0.4377; 0.6227]
Zheng,2017	GLP-1	Metformin	-0.6330 [-1.3535; 0.0875]
Jensterle,2016	GLP-1	GLP-1 + Metformin	0.1620 [-0.4048; 0.7289]
Jensterle,2015	GLP-1	Metformin	-0.6330 [-1.3535; 0.0875]

Number of studies: k = 14

Number of pairwise comparisons: m = 14
Number of treatments: n = 5
Number of designs: d = 6

Random effects model

Treatment estimate (sm = 'MD', comparison: other treatments vs 'Placebo'):

	MD	95%-CI	z	p-value
GLP-1	-2.0007	[-2.4626; -1.5388]	-8.49	< 0.0001
GLP-1 + Metformin	-2.1627	[-2.8919; -1.4335]	-5.81	< 0.0001
Metformin	-1.3677	[-2.2204; -0.5150]	-3.14	0.0017
Myoinositol + Folic acid	-1.4602	[-2.4643; -0.4561]	-2.85	0.0044
Placebo	.	.	.	.

Quantifying heterogeneity / inconsistency:
tau^2 = 0.0613; tau = 0.2476; I^2 = 15.4% [0.0%; 55.9%]

Tests of heterogeneity (within designs) and inconsistency (between designs):

	Q	d.f.	p-value
Total	11.83	10	0.2969
Within designs	11.74	8	0.1634
Between designs	0.09	2	0.9561

Details of network meta-analysis methods:
- Frequentist graph-theoretical approach
- DerSimonian-Laird estimator for tau^2
- Calculation of I^2 based on Q

[[8]]
Original data:

	treat1	treat2	TE	seTE
Wen,2023	GLP-1 + Metformin	Metformin	-0.9500	0.3100
Xing,2022	GLP-1 + Metformin	Metformin	-1.5300	1.2700
Elkind-Hirsch,2022	GLP-1	Placebo	-2.0000	0.5100
Ma,2021	GLP-1 + Metformin	Metformin	-0.6300	0.3200
Soldat-Stanković,2022	Metformin	Myoinositol + Folic acid	0.1900	1.6500
Tao,2021	GLP-1	Metformin	-0.1900	1.0200
Zheng,2019	GLP-1	Metformin	-0.7600	0.6900
Frøssing,2018	GLP-1	Placebo	-2.0000	0.0790
Jensterle,2017	GLP-1	GLP-1 + Metformin	-0.9000	0.4200
Zahra,2016	Metformin	Placebo	-1.5000	3.2700
Ravn,2022	Metformin	Myoinositol + Folic acid	-1.2600	3.1700
Zheng,2017	GLP-1	Metformin	-0.7600	1.5000

Jensterle,2016 GLP-1 GLP-1 + Metformin 0.8000 0.3100
Jensterle,2015 GLP-1 Metformin -0.2000 2.7800

Number of treatment arms (by study):

	narms
Wen,2023	2
Xing,2022	2
Elkind-Hirsch,2022	2
Ma,2021	2
Soldat-Stanković,2022	2
Tao,2021	2
Zheng,2019	2
Frøssing,2018	2
Jensterle,2017	2
Zahra,2016	2
Ravn,2022	2
Zheng,2017	2
Jensterle,2016	2
Jensterle,2015	2

Results (random effects model):

	treat1	treat2	MD	95%-CI
Wen,2023	GLP-1 + Metformin	Metformin	-0.8068	[-1.2980; -0.3155]
Xing,2022	GLP-1 + Metformin	Metformin	-0.8068	[-1.2980; -0.3155]
Elkind-Hirsch,2022	GLP-1	Placebo	-2.0008	[-2.4530; -1.5486]
Ma,2021	GLP-1 + Metformin	Metformin	-0.8068	[-1.2980; -0.3155]
Soldat-Stanković,2022	Metformin	Myoinositol + Folic acid	-0.1228	[-3.0171; 2.7714]
Tao,2021	GLP-1	Metformin	-0.6569	[-1.2967; -0.0171]
Zheng,2019	GLP-1	Metformin	-0.6569	[-1.2967; -0.0171]
Frøssing,2018	GLP-1	Placebo	-2.0008	[-2.4530; -1.5486]
Jensterle,2017	GLP-1	GLP-1 + Metformin	0.1499	[-0.3839; 0.6836]
Zahra,2016	Metformin	Placebo	-1.3439	[-2.1247; -0.5630]
Ravn,2022	Metformin	Myoinositol + Folic acid	-0.1228	[-3.0171; 2.7714]
Zheng,2017	GLP-1	Metformin	-0.6569	[-1.2967; -0.0171]
Jensterle,2016	GLP-1	GLP-1 + Metformin	0.1499	[-0.3839; 0.6836]
Jensterle,2015	GLP-1	Metformin	-0.6569	[-1.2967; -0.0171]

Number of studies: k = 14
Number of pairwise comparisons: m = 14
Number of treatments: n = 5
Number of designs: d = 6

Random effects model

```
Treatment estimate (sm = 'MD', comparison: other treatments vs 'Placebo'):
                              MD             95%-CI     z p-value
GLP-1                    -2.0008 [-2.4530; -1.5486] -8.67 < 0.0001
GLP-1 + Metformin        -2.1506 [-2.8486; -1.4527] -6.04 < 0.0001
Metformin                -1.3439 [-2.1247; -0.5630] -3.37 0.0007
Myoinositol + Folic acid -1.2210 [-4.2188; 1.7767] -0.80 0.4247
Placebo                        .                 .     .       .

Quantifying heterogeneity / inconsistency:
tau^2 = 0.0581; tau = 0.2410; I^2 = 15.7% [0.0%; 56.2%]

Tests of heterogeneity (within designs) and inconsistency (between designs):
                    Q d.f. p-value
Total           11.86 10 0.2945
Within designs 11.86     8 0.1577
Between designs 0.00     2 0.9985

Details of network meta-analysis methods:
- Frequentist graph-theoretical approach
- DerSimonian-Laird estimator for tau^2
- Calculation of I^2 based on Q

[[9]]
Original data:

                               treat1                   treat2      TE  seTE
Wen,2023              GLP-1 + Metformin                Metformin -0.9500 0.3100
Xing,2022             GLP-1 + Metformin                Metformin -1.5300 1.2700
Elkind-Hirsch,2022                GLP-1                  Placebo -2.0000 0.5100
Ma,2021               GLP-1 + Metformin                Metformin -0.6300 0.3200
Soldat-Stanković,2022         Metformin Myoinositol + Folic acid 0.1900 1.6500
Tao,2021                          GLP-1                Metformin -0.1900 1.0200
Zheng,2019                        GLP-1                Metformin -0.7600 0.6900
Shokrpour,2019                Metformin Myoinositol + Folic acid 0.1000 0.1200
Jensterle,2017                    GLP-1        GLP-1 + Metformin -0.9000 0.4200
Zahra,2016                    Metformin                  Placebo -1.5000 3.2700
Ravn,2022                     Metformin Myoinositol + Folic acid -1.2600 3.1700
Zheng,2017                        GLP-1                Metformin -0.7600 1.5000
Jensterle,2016                    GLP-1        GLP-1 + Metformin 0.8000 0.3100
Jensterle,2015                    GLP-1                Metformin -0.2000 2.7800

Number of treatment arms (by study):
                        narms
```

Wen,2023	2
Xing,2022	2
Elkind-Hirsch,2022	2
Ma,2021	2
Soldat-Stanković,2022	2
Tao,2021	2
Zheng,2019	2
Shokrpour,2019	2
Jensterle,2017	2
Zahra,2016	2
Ravn,2022	2
Zheng,2017	2
Jensterle,2016	2
Jensterle,2015	2

Results (random effects model):

	treat1	treat2	MD	95%-CI
Wen,2023	GLP-1 + Metformin	Metformin	-0.8054	[-1.3180; -0.2928]
Xing,2022	GLP-1 + Metformin	Metformin	-0.8054	[-1.3180; -0.2928]
Elkind-Hirsch,2022	GLP-1	Placebo	-2.0050	[-3.1215; -0.8884]
Ma,2021	GLP-1 + Metformin	Metformin	-0.8054	[-1.3180; -0.2928]
Soldat-Stanković,2022	Metformin	Myoinositol + Folic acid	0.0913	[-0.4813; 0.6639]
Tao,2021	GLP-1	Metformin	-0.6645	[-1.3253; -0.0036]
Zheng,2019	GLP-1	Metformin	-0.6645	[-1.3253; -0.0036]
Shokrpour,2019	Metformin	Myoinositol + Folic acid	0.0913	[-0.4813; 0.6639]
Jensterle,2017	GLP-1	GLP-1 + Metformin	0.1410	[-0.4138; 0.6957]
Zahra,2016	Metformin	Placebo	-1.3405	[-2.6278; -0.0532]
Ravn,2022	Metformin	Myoinositol + Folic acid	0.0913	[-0.4813; 0.6639]
Zheng,2017	GLP-1	Metformin	-0.6645	[-1.3253; -0.0036]
Jensterle,2016	GLP-1	GLP-1 + Metformin	0.1410	[-0.4138; 0.6957]
Jensterle,2015	GLP-1	Metformin	-0.6645	[-1.3253; -0.0036]

Number of studies: k = 14
Number of pairwise comparisons: m = 14
Number of treatments: n = 5
Number of designs: d = 6

Random effects model

Treatment estimate (sm = 'MD', comparison: other treatments vs 'Placebo'):

	MD	95%-CI	z	p-value
GLP-1	-2.0050	[-3.1215; -0.8884]	-3.52	0.0004
GLP-1 + Metformin	-2.1459	[-3.3868; -0.9050]	-3.39	0.0007

```
Metformin                -1.3405 [-2.6278; -0.0532] -2.04 0.0413
Myoinositol + Folic acid -1.4318 [-2.8407; -0.0229] -1.99 0.0464
Placebo                        .                  .     .       .

Quantifying heterogeneity / inconsistency:
tau^2 = 0.0744; tau = 0.2728; I^2 = 15.8% [0.0%; 56.3%]

Tests of heterogeneity (within designs) and inconsistency (between designs):
                    Q d.f. p-value
Total           11.88 10 0.2930
Within designs 11.88     8 0.1566
Between designs 0.00     2 0.9985

Details of network meta-analysis methods:
- Frequentist graph-theoretical approach
- DerSimonian-Laird estimator for tau^2
- Calculation of I^2 based on Q

[[10]]
Original data:

                              treat1                  treat2      TE  seTE
Wen,2023              GLP-1 + Metformin               Metformin -0.9500 0.3100
Xing,2022             GLP-1 + Metformin               Metformin -1.5300 1.2700
Elkind-Hirsch,2022                GLP-1                 Placebo -2.0000 0.5100
Ma,2021               GLP-1 + Metformin               Metformin -0.6300 0.3200
Soldat-Stanković,2022         Metformin Myoinositol + Folic acid 0.1900 1.6500
Tao,2021                          GLP-1               Metformin -0.1900 1.0200
Zheng,2019                        GLP-1               Metformin -0.7600 0.6900
Shokrpour,2019                Metformin Myoinositol + Folic acid 0.1000 0.1200
Frøssing,2018                     GLP-1                 Placebo -2.0000 0.0790
Zahra,2016                    Metformin                 Placebo -1.5000 3.2700
Ravn,2022                     Metformin Myoinositol + Folic acid -1.2600 3.1700
Zheng,2017                        GLP-1               Metformin -0.7600 1.5000
Jensterle,2016                    GLP-1       GLP-1 + Metformin 0.8000 0.3100
Jensterle,2015                    GLP-1               Metformin -0.2000 2.7800

Number of treatment arms (by study):
                     narms
Wen,2023                 2
Xing,2022                2
Elkind-Hirsch,2022       2
Ma,2021                  2
Soldat-Stanković,2022    2
```

```
Tao,2021             2
Zheng,2019           2
Shokrpour,2019       2
Frøssing,2018        2
Zahra,2016           2
Ravn,2022            2
Zheng,2017           2
Jensterle,2016       2
Jensterle,2015       2

Results (random effects model):

                          treat1                 treat2      MD            95%-CI
Wen,2023              GLP-1 + Metformin               Metformin -0.8835 [-1.2875; -0.4795]
Xing,2022             GLP-1 + Metformin               Metformin -0.8835 [-1.2875; -0.4795]
Elkind-Hirsch,2022              GLP-1                 Placebo -1.9998 [-2.1528; -1.8469]
Ma,2021               GLP-1 + Metformin               Metformin -0.8835 [-1.2875; -0.4795]
Soldat-Stanković,2022         Metformin Myoinositol + Folic acid 0.0985 [-0.1359; 0.3329]
Tao,2021                        GLP-1                 Metformin -0.2164 [-0.8165; 0.3838]
Zheng,2019                      GLP-1                 Metformin -0.2164 [-0.8165; 0.3838]
Shokrpour,2019                Metformin Myoinositol + Folic acid 0.0985 [-0.1359; 0.3329]
Frøssing,2018                   GLP-1                 Placebo -1.9998 [-2.1528; -1.8469]
Zahra,2016                   Metformin                Placebo -1.7835 [-2.4025; -1.1644]
Ravn,2022                    Metformin Myoinositol + Folic acid 0.0985 [-0.1359; 0.3329]
Zheng,2017                      GLP-1                 Metformin -0.2164 [-0.8165; 0.3838]
Jensterle,2016                  GLP-1       GLP-1 + Metformin 0.6671 [ 0.1350; 1.1992]
Jensterle,2015                  GLP-1                 Metformin -0.2164 [-0.8165; 0.3838]

Number of studies: k = 14
Number of pairwise comparisons: m = 14
Number of treatments: n = 5
Number of designs: d = 6

Random effects model

Treatment estimate (sm = 'MD', comparison: other treatments vs 'Placebo'):
                          MD            95%-CI      z p-value
GLP-1                   -1.9998 [-2.1528; -1.8469] -25.62 < 0.0001
GLP-1 + Metformin       -2.6670 [-3.2204; -2.1136] -9.45 < 0.0001
Metformin               -1.7835 [-2.4025; -1.1644] -5.65 < 0.0001
Myoinositol + Folic acid -1.8820 [-2.5440; -1.2201] -5.57 < 0.0001
Placebo                        .                 .      .       .

Quantifying heterogeneity / inconsistency:
```

tau^2 = 0; tau = 0; I^2 = 0% [0.0%; 60.2%]

Tests of heterogeneity (within designs) and inconsistency (between designs):

	Q	d.f.	p-value
Total	2.06	10	0.9958
Within designs	1.27	8	0.9958
Between designs	0.79	2	0.6740

Details of network meta-analysis methods:
- Frequentist graph-theoretical approach
- DerSimonian-Laird estimator for tau^2
- Calculation of I^2 based on Q

[[11]]
Original data:

	treat1	treat2	TE	seTE
Wen,2023	GLP-1 + Metformin	Metformin	-0.9500	0.3100
Xing,2022	GLP-1 + Metformin	Metformin	-1.5300	1.2700
Elkind-Hirsch,2022	GLP-1	Placebo	-2.0000	0.5100
Ma,2021	GLP-1 + Metformin	Metformin	-0.6300	0.3200
Soldat-Stanković,2022	Metformin	Myoinositol + Folic acid	0.1900	1.6500
Tao,2021	GLP-1	Metformin	-0.1900	1.0200
Zheng,2019	GLP-1	Metformin	-0.7600	0.6900
Shokrpour,2019	Metformin	Myoinositol + Folic acid	0.1000	0.1200
Frøssing,2018	GLP-1	Placebo	-2.0000	0.0790
Jensterle,2017	GLP-1	GLP-1 + Metformin	-0.9000	0.4200
Ravn,2022	Metformin	Myoinositol + Folic acid	-1.2600	3.1700
Zheng,2017	GLP-1	Metformin	-0.7600	1.5000
Jensterle,2016	GLP-1	GLP-1 + Metformin	0.8000	0.3100
Jensterle,2015	GLP-1	Metformin	-0.2000	2.7800

Number of treatment arms (by study):

	narms
Wen,2023	2
Xing,2022	2
Elkind-Hirsch,2022	2
Ma,2021	2
Soldat-Stanković,2022	2
Tao,2021	2
Zheng,2019	2
Shokrpour,2019	2
Frøssing,2018	2
Jensterle,2017	2

Ravn,2022	2
Zheng,2017	2
Jensterle,2016	2
Jensterle,2015	2

Results (random effects model):

	treat1	treat2	MD	95%-CI
Wen,2023	GLP-1 + Metformin	Metformin	-0.8075	[-1.2991; -0.3159]
Xing,2022	GLP-1 + Metformin	Metformin	-0.8075	[-1.2991; -0.3159]
Elkind-Hirsch,2022	GLP-1	Placebo	-2.0000	[-2.4524; -1.5476]
Ma,2021	GLP-1 + Metformin	Metformin	-0.8075	[-1.2991; -0.3159]
Soldat-Stanković,2022	Metformin	Myoinositol + Folic acid	0.0929	[-0.4251; 0.6108]
Tao,2021	GLP-1	Metformin	-0.6583	[-1.3008; -0.0158]
Zheng,2019	GLP-1	Metformin	-0.6583	[-1.3008; -0.0158]
Shokrpour,2019	Metformin	Myoinositol + Folic acid	0.0929	[-0.4251; 0.6108]
Frøssing,2018	GLP-1	Placebo	-2.0000	[-2.4524; -1.5476]
Jensterle,2017	GLP-1	GLP-1 + Metformin	0.1492	[-0.3853; 0.6837]
Ravn,2022	Metformin	Myoinositol + Folic acid	0.0929	[-0.4251; 0.6108]
Zheng,2017	GLP-1	Metformin	-0.6583	[-1.3008; -0.0158]
Jensterle,2016	GLP-1	GLP-1 + Metformin	0.1492	[-0.3853; 0.6837]
Jensterle,2015	GLP-1	Metformin	-0.6583	[-1.3008; -0.0158]

Number of studies: k = 14
Number of pairwise comparisons: m = 14
Number of treatments: n = 5
Number of designs: d = 5

Random effects model

Treatment estimate (sm = 'MD', comparison: other treatments vs 'Placebo'):

	MD	95%-CI	z	p-value
GLP-1	-2.0000	[-2.4524; -1.5476]	-8.67	< 0.0001
GLP-1 + Metformin	-2.1492	[-2.8494; -1.4489]	-6.02	< 0.0001
Metformin	-1.3417	[-2.1275; -0.5559]	-3.35	0.0008
Myoinositol + Folic acid	-1.4346	[-2.3757; -0.4934]	-2.99	0.0028
Placebo	.	.	.	.

Quantifying heterogeneity / inconsistency:
$\tau^2 = 0.0578$; $\tau = 0.2403$; $I^2 = 15.8\%$ [0.0%; 56.3%]

(printed as: tau^2 = 0.0578; tau = 0.2403; I^2 = 15.8% [0.0%; 56.3%])

Tests of heterogeneity (within designs) and inconsistency (between designs):

	Q	d.f.	p-value
Total	11.88	10	0.2930

Within designs 11.88 9 0.2202
Between designs 0.00 1 0.9651

Details of network meta-analysis methods:
- Frequentist graph-theoretical approach
- DerSimonian-Laird estimator for tau^2
- Calculation of I^2 based on Q

[[12]]
Original data:

	treat1	treat2	TE	seTE
Wen,2023	GLP-1 + Metformin	Metformin	-0.9500	0.3100
Xing,2022	GLP-1 + Metformin	Metformin	-1.5300	1.2700
Elkind-Hirsch,2022	GLP-1	Placebo	-2.0000	0.5100
Ma,2021	GLP-1 + Metformin	Metformin	-0.6300	0.3200
Soldat-Stanković,2022	Metformin	Myoinositol + Folic acid	0.1900	1.6500
Tao,2021	GLP-1	Metformin	-0.1900	1.0200
Zheng,2019	GLP-1	Metformin	-0.7600	0.6900
Shokrpour,2019	Metformin	Myoinositol + Folic acid	0.1000	0.1200
Frøssing,2018	GLP-1	Placebo	-2.0000	0.0790
Jensterle,2017	GLP-1	GLP-1 + Metformin	-0.9000	0.4200
Zahra,2016	Metformin	Placebo	-1.5000	3.2700
Zheng,2017	GLP-1	Metformin	-0.7600	1.5000
Jensterle,2016	GLP-1	GLP-1 + Metformin	0.8000	0.3100
Jensterle,2015	GLP-1	Metformin	-0.2000	2.7800

Number of treatment arms (by study):

	narms
Wen,2023	2
Xing,2022	2
Elkind-Hirsch,2022	2
Ma,2021	2
Soldat-Stanković,2022	2
Tao,2021	2
Zheng,2019	2
Shokrpour,2019	2
Frøssing,2018	2
Jensterle,2017	2
Zahra,2016	2
Zheng,2017	2
Jensterle,2016	2
Jensterle,2015	2

Results (random effects model):

	treat1	treat2	MD	95%-CI
Wen,2023	GLP-1 + Metformin	Metformin	-0.8073	[-1.2903; -0.3242]
Xing,2022	GLP-1 + Metformin	Metformin	-0.8073	[-1.2903; -0.3242]
Elkind-Hirsch,2022	GLP-1	Placebo	-2.0007	[-2.4342; -1.5672]
Ma,2021	GLP-1 + Metformin	Metformin	-0.8073	[-1.2903; -0.3242]
Soldat-Stanković,2022	Metformin	Myoinositol + Folic acid	0.1021	[-0.3973; 0.6015]
Tao,2021	GLP-1	Metformin	-0.6537	[-1.2853; -0.0220]
Zheng,2019	GLP-1	Metformin	-0.6537	[-1.2853; -0.0220]
Shokrpour,2019	Metformin	Myoinositol + Folic acid	0.1021	[-0.3973; 0.6015]
Frøssing,2018	GLP-1	Placebo	-2.0007	[-2.4342; -1.5672]
Jensterle,2017	GLP-1	GLP-1 + Metformin	0.1536	[-0.3722; 0.6794]
Zahra,2016	Metformin	Placebo	-1.3470	[-2.1108; -0.5833]
Zheng,2017	GLP-1	Metformin	-0.6537	[-1.2853; -0.0220]
Jensterle,2016	GLP-1	GLP-1 + Metformin	0.1536	[-0.3722; 0.6794]
Jensterle,2015	GLP-1	Metformin	-0.6537	[-1.2853; -0.0220]

Number of studies: k = 14
Number of pairwise comparisons: m = 14
Number of treatments: n = 5
Number of designs: d = 6

Random effects model

Treatment estimate (sm = 'MD', comparison: other treatments vs 'Placebo'):

	MD	95%-CI	z	p-value
GLP-1	-2.0007	[-2.4342; -1.5672]	-9.05	< 0.0001
GLP-1 + Metformin	-2.1543	[-2.8343; -1.4743]	-6.21	< 0.0001
Metformin	-1.3470	[-2.1108; -0.5833]	-3.46	0.0005
Myoinositol + Folic acid	-1.4491	[-2.3617; -0.5366]	-3.11	0.0019
Placebo	.	.	.	.

Quantifying heterogeneity / inconsistency:
tau^2 = 0.0521; tau = 0.2282; I^2 = 14.5% [0.0%; 55.1%]

Tests of heterogeneity (within designs) and inconsistency (between designs):

	Q	d.f.	p-value
Total	11.7	10	0.3057
Within designs	11.7	8	0.1653
Between designs	0.0	2	0.9985

Details of network meta-analysis methods:
- Frequentist graph-theoretical approach

- DerSimonian-Laird estimator for tau^2
- Calculation of I^2 based on Q

[[13]]
Original data:

	treat1	treat2	TE	seTE
Wen,2023	GLP-1 + Metformin	Metformin	-0.9500	0.3100
Xing,2022	GLP-1 + Metformin	Metformin	-1.5300	1.2700
Elkind-Hirsch,2022	GLP-1	Placebo	-2.0000	0.5100
Ma,2021	GLP-1 + Metformin	Metformin	-0.6300	0.3200
Soldat-Stanković,2022	Metformin	Myoinositol + Folic acid	0.1900	1.6500
Tao,2021	GLP-1	Metformin	-0.1900	1.0200
Zheng,2019	GLP-1	Metformin	-0.7600	0.6900
Shokrpour,2019	Metformin	Myoinositol + Folic acid	0.1000	0.1200
Frøssing,2018	GLP-1	Placebo	-2.0000	0.0790
Jensterle,2017	GLP-1	GLP-1 + Metformin	-0.9000	0.4200
Zahra,2016	Metformin	Placebo	-1.5000	3.2700
Ravn,2022	Metformin	Myoinositol + Folic acid	-1.2600	3.1700
Jensterle,2016	GLP-1	GLP-1 + Metformin	0.8000	0.3100
Jensterle,2015	GLP-1	Metformin	-0.2000	2.7800

Number of treatment arms (by study):

	narms
Wen,2023	2
Xing,2022	2
Elkind-Hirsch,2022	2
Ma,2021	2
Soldat-Stanković,2022	2
Tao,2021	2
Zheng,2019	2
Shokrpour,2019	2
Frøssing,2018	2
Jensterle,2017	2
Zahra,2016	2
Ravn,2022	2
Jensterle,2016	2
Jensterle,2015	2

Results (random effects model):

	treat1	treat2	MD	95%-CI
Wen,2023	GLP-1 + Metformin	Metformin	-0.8045	[-1.3000; -0.3090]
Xing,2022	GLP-1 + Metformin	Metformin	-0.8045	[-1.3000; -0.3090]

Elkind-Hirsch,2022 GLP-1 Placebo -2.0008 [-2.4535; -1.5480]
Ma,2021 GLP-1 + Metformin Metformin -0.8045 [-1.3000; -0.3090]
Soldat-Stanković,2022 Metformin Myoinositol + Folic acid 0.0928 [-0.4269; 0.6125]
Tao,2021 GLP-1 Metformin -0.6520 [-1.3073; 0.0033]
Zheng,2019 GLP-1 Metformin -0.6520 [-1.3073; 0.0033]
Shokrpour,2019 Metformin Myoinositol + Folic acid 0.0928 [-0.4269; 0.6125]
Frøssing,2018 GLP-1 Placebo -2.0008 [-2.4535; -1.5480]
Jensterle,2017 GLP-1 GLP-1 + Metformin 0.1525 [-0.3872; 0.6922]
Zahra,2016 Metformin Placebo -1.3488 [-2.1426; -0.5549]
Ravn,2022 Metformin Myoinositol + Folic acid 0.0928 [-0.4269; 0.6125]
Jensterle,2016 GLP-1 GLP-1 + Metformin 0.1525 [-0.3872; 0.6922]
Jensterle,2015 GLP-1 Metformin -0.6520 [-1.3073; 0.0033]

Number of studies: k = 14
Number of pairwise comparisons: m = 14
Number of treatments: n = 5
Number of designs: d = 6

Random effects model

Treatment estimate (sm = 'MD', comparison: other treatments vs 'Placebo'):

	MD	95%-CI	z	p-value
GLP-1	-2.0008	[-2.4535; -1.5480]	-8.66	< 0.0001
GLP-1 + Metformin	-2.1533	[-2.8560; -1.4505]	-6.01	< 0.0001
Metformin	-1.3488	[-2.1426; -0.5549]	-3.33	0.0009
Myoinositol + Folic acid	-1.4416	[-2.3904; -0.4927]	-2.98	0.0029
Placebo	.	.	.	.

Quantifying heterogeneity / inconsistency:
tau^2 = 0.0583; tau = 0.2414; I^2 = 15.8% [0.0%; 56.2%]

Tests of heterogeneity (within designs) and inconsistency (between designs):

	Q	d.f.	p-value
Total	11.87	10	0.2937
Within designs	11.87	8	0.1573
Between designs	0.01	2	0.9965

Details of network meta-analysis methods:
- Frequentist graph-theoretical approach
- DerSimonian-Laird estimator for tau^2
- Calculation of I^2 based on Q

[[14]]
Original data:

	treat1	treat2	TE	seTE
Wen,2023	GLP-1 + Metformin	Metformin	-0.9500	0.3100
Xing,2022	GLP-1 + Metformin	Metformin	-1.5300	1.2700
Elkind-Hirsch,2022	GLP-1	Placebo	-2.0000	0.5100
Ma,2021	GLP-1 + Metformin	Metformin	-0.6300	0.3200
Soldat-Stanković,2022	Metformin	Myoinositol + Folic acid	0.1900	1.6500
Tao,2021	GLP-1	Metformin	-0.1900	1.0200
Zheng,2019	GLP-1	Metformin	-0.7600	0.6900
Shokrpour,2019	Metformin	Myoinositol + Folic acid	0.1000	0.1200
Frøssing,2018	GLP-1	Placebo	-2.0000	0.0790
Jensterle,2017	GLP-1	GLP-1 + Metformin	-0.9000	0.4200
Zahra,2016	Metformin	Placebo	-1.5000	3.2700
Ravn,2022	Metformin	Myoinositol + Folic acid	-1.2600	3.1700
Zheng,2017	GLP-1	Metformin	-0.7600	1.5000
Jensterle,2015	GLP-1	Metformin	-0.2000	2.7800

Number of treatment arms (by study):

	narms
Wen,2023	2
Xing,2022	2
Elkind-Hirsch,2022	2
Ma,2021	2
Soldat-Stanković,2022	2
Tao,2021	2
Zheng,2019	2
Shokrpour,2019	2
Frøssing,2018	2
Jensterle,2017	2
Zahra,2016	2
Ravn,2022	2
Zheng,2017	2
Jensterle,2015	2

Results (random effects model):

	treat1	treat2	MD	95%-CI
Wen,2023	GLP-1 + Metformin	Metformin	-0.7067	[-1.1150; -0.2984]
Xing,2022	GLP-1 + Metformin	Metformin	-0.7067	[-1.1150; -0.2984]
Elkind-Hirsch,2022	GLP-1	Placebo	-2.0004	[-2.1534; -1.8474]
Ma,2021	GLP-1 + Metformin	Metformin	-0.7067	[-1.1150; -0.2984]
Soldat-Stanković,2022	Metformin	Myoinositol + Folic acid	0.0985	[-0.1359; 0.3329]
Tao,2021	GLP-1	Metformin	-1.2021	[-1.8873; -0.5170]
Zheng,2019	GLP-1	Metformin	-1.2021	[-1.8873; -0.5170]

```
Shokrpour,2019            Metformin Myoinositol + Folic acid 0.0985 [-0.1359; 0.3329]
Frøssing,2018                 GLP-1              Placebo -2.0004 [-2.1534; -1.8474]
Jensterle,2017                GLP-1      GLP-1 + Metformin -0.4955 [-1.1550; 0.1641]
Zahra,2016                Metformin              Placebo -0.7983 [-1.4999; -0.0966]
Ravn,2022                 Metformin Myoinositol + Folic acid 0.0985 [-0.1359; 0.3329]
Zheng,2017                    GLP-1            Metformin -1.2021 [-1.8873; -0.5170]
Jensterle,2015                GLP-1            Metformin -1.2021 [-1.8873; -0.5170]

Number of studies: k = 14
Number of pairwise comparisons: m = 14
Number of treatments: n = 5
Number of designs: d = 6

Random effects model

Treatment estimate (sm = 'MD', comparison: other treatments vs 'Placebo'):
                            MD             95%-CI      z p-value
GLP-1                  -2.0004 [-2.1534; -1.8474] -25.63 < 0.0001
GLP-1 + Metformin        -1.5049 [-2.1817; -0.8282] -4.36 < 0.0001
Metformin               -0.7983 [-1.4999; -0.0966] -2.23  0.0258
Myoinositol + Folic acid -0.8968 [-1.6365; -0.1570] -2.38 0.0175
Placebo                        .                  .      .       .

Quantifying heterogeneity / inconsistency:
tau^2 = 0; tau = 0; I^2 = 0% [0.0%; 60.2%]

Tests of heterogeneity (within designs) and inconsistency (between designs):
                   Q d.f. p-value
Total           3.87  10 0.9532
Within designs 1.27     8 0.9958
Between designs 2.59    2 0.2736

Details of network meta-analysis methods:
- Frequentist graph-theoretical approach
- DerSimonian-Laird estimator for tau^2
- Calculation of I^2 based on Q

[[15]]
Original data:

                              treat1                  treat2      TE  seTE
Wen,2023              GLP-1 + Metformin              Metformin -0.9500 0.3100
Xing,2022             GLP-1 + Metformin              Metformin -1.5300 1.2700
Elkind-Hirsch,2022               GLP-1               Placebo -2.0000 0.5100
```

Ma,2021	GLP-1 + Metformin	Metformin	-0.6300	0.3200
Soldat-Stanković,2022	Metformin	Myoinositol + Folic acid	0.1900	1.6500
Tao,2021	GLP-1	Metformin	-0.1900	1.0200
Zheng,2019	GLP-1	Metformin	-0.7600	0.6900
Shokrpour,2019	Metformin	Myoinositol + Folic acid	0.1000	0.1200
Frøssing,2018	GLP-1	Placebo	-2.0000	0.0790
Jensterle,2017	GLP-1	GLP-1 + Metformin	-0.9000	0.4200
Zahra,2016	Metformin	Placebo	-1.5000	3.2700
Ravn,2022	Metformin	Myoinositol + Folic acid	-1.2600	3.1700
Zheng,2017	GLP-1	Metformin	-0.7600	1.5000
Jensterle,2016	GLP-1	GLP-1 + Metformin	0.8000	0.3100

Number of treatment arms (by study):

	narms
Wen,2023	2
Xing,2022	2
Elkind-Hirsch,2022	2
Ma,2021	2
Soldat-Stanković,2022	2
Tao,2021	2
Zheng,2019	2
Shokrpour,2019	2
Frøssing,2018	2
Jensterle,2017	2
Zahra,2016	2
Ravn,2022	2
Zheng,2017	2
Jensterle,2016	2

Results (random effects model):

	treat1	treat2	MD	95%-CI
Wen,2023	GLP-1 + Metformin	Metformin	-0.8097	[-1.3006; -0.3187]
Xing,2022	GLP-1 + Metformin	Metformin	-0.8097	[-1.3006; -0.3187]
Elkind-Hirsch,2022	GLP-1	Placebo	-2.0008	[-2.4499; -1.5517]
Ma,2021	GLP-1 + Metformin	Metformin	-0.8097	[-1.3006; -0.3187]
Soldat-Stanković,2022	Metformin	Myoinositol + Folic acid	0.0929	[-0.4226; 0.6085]
Tao,2021	GLP-1	Metformin	-0.6627	[-1.3055; -0.0199]
Zheng,2019	GLP-1	Metformin	-0.6627	[-1.3055; -0.0199]
Shokrpour,2019	Metformin	Myoinositol + Folic acid	0.0929	[-0.4226; 0.6085]
Frøssing,2018	GLP-1	Placebo	-2.0008	[-2.4499; -1.5517]
Jensterle,2017	GLP-1	GLP-1 + Metformin	0.1470	[-0.3871; 0.6810]
Zahra,2016	Metformin	Placebo	-1.3381	[-2.1197; -0.5565]
Ravn,2022	Metformin	Myoinositol + Folic acid	0.0929	[-0.4226; 0.6085]

Zheng,2017 GLP-1 Metformin -0.6627 [-1.3055; -0.0199]
Jensterle,2016 GLP-1 GLP-1 + Metformin 0.1470 [-0.3871; 0.6810]

Number of studies: k = 14
Number of pairwise comparisons: m = 14
Number of treatments: n = 5
Number of designs: d = 6

Random effects model

Treatment estimate (sm = 'MD', comparison: other treatments vs 'Placebo'):

	MD	95%-CI	z	p-value
GLP-1	-2.0008	[-2.4499; -1.5517]	-8.73	< 0.0001
GLP-1 + Metformin	-2.1478	[-2.8440; -1.4516]	-6.05	< 0.0001
Metformin	-1.3381	[-2.1197; -0.5565]	-3.36	0.0008
Myoinositol + Folic acid	-1.4311	[-2.3674; -0.4947]	-3.00	0.0027
Placebo	.	.	.	.

Quantifying heterogeneity / inconsistency:
tau^2 = 0.0571; tau = 0.2389; I^2 = 15.7% [0.0%; 56.2%]

Tests of heterogeneity (within designs) and inconsistency (between designs):

	Q	d.f.	p-value
Total	11.86	10	0.2944
Within designs	11.86	8	0.1576
Between designs	0.00	2	0.9992

Details of network meta-analysis methods:
- Frequentist graph-theoretical approach
- DerSimonian-Laird estimator for tau^2
- Calculation of I^2 based on Q

=== Heterogeneity & Inconsistency ===
[1] 0.07432066
Q statistics to assess homogeneity / consistency

Design-specific decomposition of within-designs Q statistic

Design	Q	df	p-value
GLP-1 vs GLP-1 + Metformin	10.61	1	0.0011
GLP-1 + Metformin vs Metformin	0.84	2	0.6568
Metformin vs Myoinositol + Folic acid	0.19	2	0.9108

GLP-1 vs Metformin 0.25 3 0.9697
Placebo vs GLP-1 0.00 1 1.0000

Between-designs Q statistic after detaching of single designs
(influential designs have p-value markedly different from 0.9985)

Detached design	Q	df	p-value
GLP-1 + Metformin vs Metformin	0.00	1	0.9785
GLP-1 vs GLP-1 + Metformin	0.00	1	0.9785
GLP-1 vs Metformin	0.00	1	0.9717
Placebo vs GLP-1	0.00	1	0.9651
Placebo vs Metformin	0.00	1	0.9651

Q statistic to assess consistency under the assumption of
a full design-by-treatment interaction random effects model

Waist Circumference Change

=== Sensitivity Analysis Results ===

=== Excluding High Risk of Bias (rob != 3) ===
❌ Not enough data to run network meta-analysis for low-risk studies.

=== Leave-One-Out Analysis ===
[[1]]
Original data:

	treat1	treat2	TE	seTE
Xing,2022	GLP-1 + Metformin	Metformin	-3.9000	0.9300
Elkind-Hirsch,2022	GLP-1	Placebo	-4.0000	0.9600
Ma,2021	GLP-1 + Metformin	Metformin	-2.9700	3.5800
Soldat-Stanković,2022	Metformin	Myoinositol + Folic acid	1.6900	4.2400
Frøssing,2018	GLP-1	Placebo	-5.2000	0.3700
Jensterle,2017	GLP-1	GLP-1 + Metformin	-2.0000	1.8900
Ravn,2022	Metformin	Myoinositol + Folic acid	1.6600	0.4600
Zheng,2017	GLP-1	Metformin	-3.1600	3.9100
Jensterle,2016	GLP-1	GLP-1 + Metformin	6.5000	7.2100
Jensterle,2015	GLP-1	Metformin	-1.8000	7.3600

Number of treatment arms (by study):

	narms
Xing,2022	2
Elkind-Hirsch,2022	2
Ma,2021	2
Soldat-Stanković,2022	2
Frøssing,2018	2
Jensterle,2017	2
Ravn,2022	2
Zheng,2017	2
Jensterle,2016	2
Jensterle,2015	2

Results (random effects model):

	treat1	treat2	MD	95%-CI
Xing,2022	GLP-1 + Metformin	Metformin	-3.7185	[-5.4377; -1.9994]
Elkind-Hirsch,2022	GLP-1	Placebo	-5.0448	[-5.7215; -4.3681]
Ma,2021	GLP-1 + Metformin	Metformin	-3.7185	[-5.4377; -1.9994]
Soldat-Stanković,2022	Metformin	Myoinositol + Folic acid	1.6603	[0.7640; 2.5567]
Frøssing,2018	GLP-1	Placebo	-5.0448	[-5.7215; -4.3681]
Jensterle,2017	GLP-1	GLP-1 + Metformin	-0.9474	[-4.1365; 2.2417]
Ravn,2022	Metformin	Myoinositol + Folic acid	1.6603	[0.7640; 2.5567]
Zheng,2017	GLP-1	Metformin	-4.6659	[-8.1056; -1.2262]
Jensterle,2016	GLP-1	GLP-1 + Metformin	-0.9474	[-4.1365; 2.2417]
Jensterle,2015	GLP-1	Metformin	-4.6659	[-8.1056; -1.2262]

Number of studies: k = 10
Number of pairwise comparisons: m = 10
Number of treatments: n = 5
Number of designs: d = 5

Random effects model

Treatment estimate (sm = 'MD', comparison: other treatments vs 'Placebo'):

	MD	95%-CI	z	p-value
GLP-1	-5.0448	[-5.7215; -4.3681]	-14.61	< 0.0001
GLP-1 + Metformin	-4.0974	[-7.3575; -0.8373]	-2.46	0.0138
Metformin	-0.3789	[-3.8845; 3.1267]	-0.21	0.8322
Myoinositol + Folic acid	-2.0392	[-5.6576; 1.5792]	-1.10	0.2693
Placebo	.	.	.	.

Quantifying heterogeneity / inconsistency:
tau^2 = 0; tau = 0; I^2 = 0% [0.0%; 70.8%]

Tests of heterogeneity (within designs) and inconsistency (between designs):

	Q	d.f.	p-value
Total	3.12	6	0.7937
Within designs	2.75	5	0.7383
Between designs	0.37	1	0.5438

Details of network meta-analysis methods:
- Frequentist graph-theoretical approach
- DerSimonian-Laird estimator for tau^2
- Calculation of I^2 based on Q

[[2]]
Original data:

	treat1	treat2	TE	seTE
Wen,2023	GLP-1 + Metformin	Metformin	-0.3500	0.5100
Elkind-Hirsch,2022	GLP-1	Placebo	-4.0000	0.9600
Ma,2021	GLP-1 + Metformin	Metformin	-2.9700	3.5800
Soldat-Stanković,2022	Metformin	Myoinositol + Folic acid	1.6900	4.2400
Frøssing,2018	GLP-1	Placebo	-5.2000	0.3700
Jensterle,2017	GLP-1	GLP-1 + Metformin	-2.0000	1.8900
Ravn,2022	Metformin	Myoinositol + Folic acid	1.6600	0.4600
Zheng,2017	GLP-1	Metformin	-3.1600	3.9100
Jensterle,2016	GLP-1	GLP-1 + Metformin	6.5000	7.2100
Jensterle,2015	GLP-1	Metformin	-1.8000	7.3600

Number of treatment arms (by study):

	narms
Wen,2023	2
Elkind-Hirsch,2022	2
Ma,2021	2
Soldat-Stanković,2022	2
Frøssing,2018	2
Jensterle,2017	2
Ravn,2022	2
Zheng,2017	2
Jensterle,2016	2
Jensterle,2015	2

Results (random effects model):

	treat1	treat2	MD	95%-CI
Wen,2023	GLP-1 + Metformin	Metformin	-0.4186	[-1.4001; 0.5628]
Elkind-Hirsch,2022	GLP-1	Placebo	-5.0448	[-5.7215; -4.3681]

```
Ma,2021               GLP-1 + Metformin               Metformin -0.4186 [-1.4001; 0.5628]
Soldat-Stanković,2022         Metformin Myoinositol + Folic acid 1.6603 [ 0.7640; 2.5567]
Frøssing,2018                   GLP-1                   Placebo -5.0448 [-5.7215; -4.3681]
Jensterle,2017                  GLP-1        GLP-1 + Metformin -1.6699 [-4.8440; 1.5041]
Ravn,2022                   Metformin Myoinositol + Folic acid 1.6603 [ 0.7640; 2.5567]
Zheng,2017                      GLP-1                Metformin -2.0885 [-5.3468; 1.1697]
Jensterle,2016                  GLP-1        GLP-1 + Metformin -1.6699 [-4.8440; 1.5041]
Jensterle,2015                  GLP-1                Metformin -2.0885 [-5.3468; 1.1697]

Number of studies: k = 10
Number of pairwise comparisons: m = 10
Number of treatments: n = 5
Number of designs: d = 5

Random effects model

Treatment estimate (sm = 'MD', comparison: other treatments vs 'Placebo'):
                              MD             95%-CI      z p-value
GLP-1                    -5.0448 [-5.7215; -4.3681] -14.61 < 0.0001
GLP-1 + Metformin        -3.3749 [-6.6203; -0.1295] -2.04  0.0415
Metformin                -2.9563 [-6.2840; 0.3715] -1.74  0.0817
Myoinositol + Folic acid -4.6166 [-8.0630; -1.1703] -2.63 0.0087
Placebo                        .                  .      .       .

Quantifying heterogeneity / inconsistency:
tau^2 = 0; tau = 0; I^2 = 0% [0.0%; 70.8%]

Tests of heterogeneity (within designs) and inconsistency (between designs):
                   Q d.f. p-value
Total           3.28    6 0.7733
Within designs 3.21     5 0.6673
Between designs 0.07    1 0.7986

Details of network meta-analysis methods:
- Frequentist graph-theoretical approach
- DerSimonian-Laird estimator for tau^2
- Calculation of I^2 based on Q

[[3]]
Original data:

                          treat1                 treat2      TE  seTE
Wen,2023              GLP-1 + Metformin               Metformin -0.3500 0.5100
Xing,2022             GLP-1 + Metformin               Metformin -3.9000 0.9300
```

Ma,2021	GLP-1 + Metformin	Metformin	-2.9700	3.5800
Soldat-Stanković,2022	Metformin	Myoinositol + Folic acid	1.6900	4.2400
Frøssing,2018	GLP-1	Placebo	-5.2000	0.3700
Jensterle,2017	GLP-1	GLP-1 + Metformin	-2.0000	1.8900
Ravn,2022	Metformin	Myoinositol + Folic acid	1.6600	0.4600
Zheng,2017	GLP-1	Metformin	-3.1600	3.9100
Jensterle,2016	GLP-1	GLP-1 + Metformin	6.5000	7.2100
Jensterle,2015	GLP-1	Metformin	-1.8000	7.3600

Number of treatment arms (by study):

	narms
Wen,2023	2
Xing,2022	2
Ma,2021	2
Soldat-Stanković,2022	2
Frøssing,2018	2
Jensterle,2017	2
Ravn,2022	2
Zheng,2017	2
Jensterle,2016	2
Jensterle,2015	2

Results (random effects model):

	treat1	treat2	MD	95%-CI
Wen,2023	GLP-1 + Metformin	Metformin	-2.0525	[-4.4469; 0.3419]
Xing,2022	GLP-1 + Metformin	Metformin	-2.0525	[-4.4469; 0.3419]
Ma,2021	GLP-1 + Metformin	Metformin	-2.0525	[-4.4469; 0.3419]
Soldat-Stanković,2022	Metformin	Myoinositol + Folic acid	1.6640	[-1.6322; 4.9603]
Frøssing,2018	GLP-1	Placebo	-5.2000	[-8.7024; -1.6976]
Jensterle,2017	GLP-1	GLP-1 + Metformin	-0.9941	[-5.0499; 3.0617]
Ravn,2022	Metformin	Myoinositol + Folic acid	1.6640	[-1.6322; 4.9603]
Zheng,2017	GLP-1	Metformin	-3.0466	[-7.3777; 1.2845]
Jensterle,2016	GLP-1	GLP-1 + Metformin	-0.9941	[-5.0499; 3.0617]
Jensterle,2015	GLP-1	Metformin	-3.0466	[-7.3777; 1.2845]

Number of studies: k = 10
Number of pairwise comparisons: m = 10
Number of treatments: n = 5
Number of designs: d = 5

Random effects model

Treatment estimate (sm = 'MD', comparison: other treatments vs 'Placebo'):

```
                         MD             95%-CI     z p-value
GLP-1                    -5.2000 [ -8.7024; -1.6976] -2.91 0.0036
GLP-1 + Metformin        -4.2059 [ -9.5647; 1.1528] -1.54 0.1240
Metformin                -2.1534 [ -7.7234; 3.4166] -0.76 0.4486
Myoinositol + Folic acid -3.8174 [-10.2897; 2.6548] -1.16 0.2477
Placebo                        .                  .     .      .
```

Quantifying heterogeneity / inconsistency:
tau^2 = 3.0563; tau = 1.7482; I^2 = 53.1% [0.0%; 80.0%]

Tests of heterogeneity (within designs) and inconsistency (between designs):

```
                    Q d.f. p-value
Total           12.78    6 0.0467
Within designs 12.78     5 0.0255
Between designs 0.00     1 0.9577
```

Details of network meta-analysis methods:
- Frequentist graph-theoretical approach
- DerSimonian-Laird estimator for tau^2
- Calculation of I^2 based on Q

[[4]]
Original data:

```
                            treat1                  treat2      TE  seTE
Wen,2023               GLP-1 + Metformin               Metformin -0.3500 0.5100
Xing,2022              GLP-1 + Metformin               Metformin -3.9000 0.9300
Elkind-Hirsch,2022               GLP-1                 Placebo -4.0000 0.9600
Soldat-Stanković,2022        Metformin Myoinositol + Folic acid 1.6900 4.2400
Frøssing,2018                    GLP-1                 Placebo -5.2000 0.3700
Jensterle,2017                   GLP-1       GLP-1 + Metformin -2.0000 1.8900
Ravn,2022                    Metformin Myoinositol + Folic acid 1.6600 0.4600
Zheng,2017                       GLP-1               Metformin -3.1600 3.9100
Jensterle,2016                   GLP-1       GLP-1 + Metformin 6.5000 7.2100
Jensterle,2015                   GLP-1               Metformin -1.8000 7.3600
```

Number of treatment arms (by study):

```
                      narms
Wen,2023                 2
Xing,2022                2
Elkind-Hirsch,2022       2
Soldat-Stanković,2022    2
Frøssing,2018            2
Jensterle,2017           2
```

Ravn,2022 2
Zheng,2017 2
Jensterle,2016 2
Jensterle,2015 2

Results (random effects model):

	treat1	treat2	MD	95%-CI
Wen,2023	GLP-1 + Metformin	Metformin	-1.8960	[-4.0192; 0.2272]
Xing,2022	GLP-1 + Metformin	Metformin	-1.8960	[-4.0192; 0.2272]
Elkind-Hirsch,2022	GLP-1	Placebo	-4.6938	[-6.8629; -2.5246]
Soldat-Stanković,2022	Metformin	Myoinositol + Folic acid	1.6630	[-1.0923; 4.4182]
Frøssing,2018	GLP-1	Placebo	-4.6938	[-6.8629; -2.5246]
Jensterle,2017	GLP-1	GLP-1 + Metformin	-1.1343	[-4.9222; 2.6536]
Ravn,2022	Metformin	Myoinositol + Folic acid	1.6630	[-1.0923; 4.4182]
Zheng,2017	GLP-1	Metformin	-3.0303	[-7.0760; 1.0154]
Jensterle,2016	GLP-1	GLP-1 + Metformin	-1.1343	[-4.9222; 2.6536]
Jensterle,2015	GLP-1	Metformin	-3.0303	[-7.0760; 1.0154]

Number of studies: k = 10
Number of pairwise comparisons: m = 10
Number of treatments: n = 5
Number of designs: d = 5

Random effects model

Treatment estimate (sm = 'MD', comparison: other treatments vs 'Placebo'):

	MD	95%-CI	z	p-value
GLP-1	-4.6938	[-6.8629; -2.5246]	-4.24	< 0.0001
GLP-1 + Metformin	-3.5594	[-7.9244; 0.8056]	-1.60	0.1100
Metformin	-1.6634	[-6.2539; 2.9271]	-0.71	0.4776
Myoinositol + Folic acid	-3.3264	[-8.6803; 2.0275]	-1.22	0.2233
Placebo	.	.	.	.

Quantifying heterogeneity / inconsistency:
tau^2 = 1.9818; tau = 1.4078; I^2 = 56.8% [0.0%; 81.4%]

Tests of heterogeneity (within designs) and inconsistency (between designs):

	Q	d.f.	p-value
Total	13.89	6	0.0308
Within designs	13.89	5	0.0163
Between designs	0.00	1	0.9521

Details of network meta-analysis methods:

- Frequentist graph-theoretical approach
- DerSimonian-Laird estimator for tau^2
- Calculation of I^2 based on Q

[[5]]
Original data:

	treat1	treat2	TE	seTE
Wen,2023	GLP-1 + Metformin	Metformin	-0.3500	0.5100
Xing,2022	GLP-1 + Metformin	Metformin	-3.9000	0.9300
Elkind-Hirsch,2022	GLP-1	Placebo	-4.0000	0.9600
Ma,2021	GLP-1 + Metformin	Metformin	-2.9700	3.5800
Frøssing,2018	GLP-1	Placebo	-5.2000	0.3700
Jensterle,2017	GLP-1	GLP-1 + Metformin	-2.0000	1.8900
Ravn,2022	Metformin	Myoinositol + Folic acid	1.6600	0.4600
Zheng,2017	GLP-1	Metformin	-3.1600	3.9100
Jensterle,2016	GLP-1	GLP-1 + Metformin	6.5000	7.2100
Jensterle,2015	GLP-1	Metformin	-1.8000	7.3600

Number of treatment arms (by study):

	narms
Wen,2023	2
Xing,2022	2
Elkind-Hirsch,2022	2
Ma,2021	2
Frøssing,2018	2
Jensterle,2017	2
Ravn,2022	2
Zheng,2017	2
Jensterle,2016	2
Jensterle,2015	2

Results (random effects model):

	treat1	treat2	MD	95%-CI
Wen,2023	GLP-1 + Metformin	Metformin	-1.9800	[-4.0437; 0.0836]
Xing,2022	GLP-1 + Metformin	Metformin	-1.9800	[-4.0437; 0.0836]
Elkind-Hirsch,2022	GLP-1	Placebo	-4.6918	[-6.8855; -2.4980]
Ma,2021	GLP-1 + Metformin	Metformin	-1.9800	[-4.0437; 0.0836]
Frøssing,2018	GLP-1	Placebo	-4.6918	[-6.8855; -2.4980]
Jensterle,2017	GLP-1	GLP-1 + Metformin	-1.1060	[-4.9051; 2.6931]
Ravn,2022	Metformin	Myoinositol + Folic acid	1.6600	[-1.2785; 4.5985]
Zheng,2017	GLP-1	Metformin	-3.0860	[-7.1271; 0.9550]
Jensterle,2016	GLP-1	GLP-1 + Metformin	-1.1060	[-4.9051; 2.6931]

Jensterle,2015 GLP-1 Metformin -3.0860 [-7.1271; 0.9550]

Number of studies: k = 10
Number of pairwise comparisons: m = 10
Number of treatments: n = 5
Number of designs: d = 5

Random effects model

Treatment estimate (sm = 'MD', comparison: other treatments vs 'Placebo'):

	MD	95%-CI	z	p-value
GLP-1	-4.6918	[-6.8855; -2.4980]	-4.19	< 0.0001
GLP-1 + Metformin	-3.5858	[-7.9728; 0.8012]	-1.60	0.1092
Metformin	-1.6057	[-6.2038; 2.9924]	-0.68	0.4937
Myoinositol + Folic acid	-3.2657	[-8.7226; 2.1911]	-1.17	0.2408
Placebo	.	.	.	.

Quantifying heterogeneity / inconsistency:
tau^2 = 2.0363; tau = 1.4270; I^2 = 57.6% [1.8%; 81.7%]

Tests of heterogeneity (within designs) and inconsistency (between designs):

	Q	d.f.	p-value
Total	14.14	6	0.0281
Within designs	14.14	5	0.0148
Between designs	0.00	1	0.9577

Details of network meta-analysis methods:
- Frequentist graph-theoretical approach
- DerSimonian-Laird estimator for tau^2
- Calculation of I^2 based on Q

[[6]]
Original data:

	treat1	treat2	TE	seTE
Wen,2023	GLP-1 + Metformin	Metformin	-0.3500	0.5100
Xing,2022	GLP-1 + Metformin	Metformin	-3.9000	0.9300
Elkind-Hirsch,2022	GLP-1	Placebo	-4.0000	0.9600
Ma,2021	GLP-1 + Metformin	Metformin	-2.9700	3.5800
Soldat-Stanković,2022	Metformin	Myoinositol + Folic acid	1.6900	4.2400
Jensterle,2017	GLP-1	GLP-1 + Metformin	-2.0000	1.8900
Ravn,2022	Metformin	Myoinositol + Folic acid	1.6600	0.4600
Zheng,2017	GLP-1	Metformin	-3.1600	3.9100
Jensterle,2016	GLP-1	GLP-1 + Metformin	6.5000	7.2100

Jensterle,2015 GLP-1 Metformin -1.8000 7.3600

Number of treatment arms (by study):

	narms
Wen,2023	2
Xing,2022	2
Elkind-Hirsch,2022	2
Ma,2021	2
Soldat-Stanković,2022	2
Jensterle,2017	2
Ravn,2022	2
Zheng,2017	2
Jensterle,2016	2
Jensterle,2015	2

Results (random effects model):

	treat1	treat2	MD	95%-CI
Wen,2023	GLP-1 + Metformin	Metformin	-2.0525	[-4.4469; 0.3419]
Xing,2022	GLP-1 + Metformin	Metformin	-2.0525	[-4.4469; 0.3419]
Elkind-Hirsch,2022	GLP-1	Placebo	-4.0000	[-7.9091; -0.0909]
Ma,2021	GLP-1 + Metformin	Metformin	-2.0525	[-4.4469; 0.3419]
Soldat-Stanković,2022	Metformin	Myoinositol + Folic acid	1.6640	[-1.6322; 4.9603]
Jensterle,2017	GLP-1	GLP-1 + Metformin	-0.9941	[-5.0499; 3.0617]
Ravn,2022	Metformin	Myoinositol + Folic acid	1.6640	[-1.6322; 4.9603]
Zheng,2017	GLP-1	Metformin	-3.0466	[-7.3777; 1.2845]
Jensterle,2016	GLP-1	GLP-1 + Metformin	-0.9941	[-5.0499; 3.0617]
Jensterle,2015	GLP-1	Metformin	-3.0466	[-7.3777; 1.2845]

Number of studies: k = 10
Number of pairwise comparisons: m = 10
Number of treatments: n = 5
Number of designs: d = 5

Random effects model

Treatment estimate (sm = 'MD', comparison: other treatments vs 'Placebo'):

	MD	95%-CI	z	p-value
GLP-1	-4.0000	[-7.9091; -0.0909]	-2.01	0.0449
GLP-1 + Metformin	-3.0059	[-8.6389; 2.6271]	-1.05	0.2956
Metformin	-0.9534	[-6.7877; 4.8809]	-0.32	0.7488
Myoinositol + Folic acid	-2.6174	[-9.3185; 4.0836]	-0.77	0.4439
Placebo	.	.	.	.

Quantifying heterogeneity / inconsistency:
tau^2 = 3.0563; tau = 1.7482; I^2 = 53.1% [0.0%; 80.0%]

Tests of heterogeneity (within designs) and inconsistency (between designs):

	Q	d.f.	p-value
Total	12.78	6	0.0467
Within designs	12.78	5	0.0255
Between designs	0.00	1	0.9577

Details of network meta-analysis methods:
- Frequentist graph-theoretical approach
- DerSimonian-Laird estimator for tau^2
- Calculation of I^2 based on Q

[[7]]
Original data:

	treat1	treat2	TE	seTE
Wen,2023	GLP-1 + Metformin	Metformin	-0.3500	0.5100
Xing,2022	GLP-1 + Metformin	Metformin	-3.9000	0.9300
Elkind-Hirsch,2022	GLP-1	Placebo	-4.0000	0.9600
Ma,2021	GLP-1 + Metformin	Metformin	-2.9700	3.5800
Soldat-Stanković,2022	Metformin	Myoinositol + Folic acid	1.6900	4.2400
Frøssing,2018	GLP-1	Placebo	-5.2000	0.3700
Ravn,2022	Metformin	Myoinositol + Folic acid	1.6600	0.4600
Zheng,2017	GLP-1	Metformin	-3.1600	3.9100
Jensterle,2016	GLP-1	GLP-1 + Metformin	6.5000	7.2100
Jensterle,2015	GLP-1	Metformin	-1.8000	7.3600

Number of treatment arms (by study):

	narms
Wen,2023	2
Xing,2022	2
Elkind-Hirsch,2022	2
Ma,2021	2
Soldat-Stanković,2022	2
Frøssing,2018	2
Ravn,2022	2
Zheng,2017	2
Jensterle,2016	2
Jensterle,2015	2

Results (random effects model):

	treat1	treat2	MD	95%-CI
Wen,2023	GLP-1 + Metformin	Metformin	-2.1213	[-4.2122; -0.0304]
Xing,2022	GLP-1 + Metformin	Metformin	-2.1213	[-4.2122; -0.0304]
Elkind-Hirsch,2022	GLP-1	Placebo	-4.6936	[-6.8644; -2.5229]
Ma,2021	GLP-1 + Metformin	Metformin	-2.1213	[-4.2122; -0.0304]
Soldat-Stanković,2022	Metformin	Myoinositol + Folic acid	1.6630	[-1.0943; 4.4202]
Frøssing,2018	GLP-1	Placebo	-4.6936	[-6.8644; -2.5229]
Ravn,2022	Metformin	Myoinositol + Folic acid	1.6630	[-1.0943; 4.4202]
Zheng,2017	GLP-1	Metformin	-1.4206	[-7.8187; 4.9775]
Jensterle,2016	GLP-1	GLP-1 + Metformin	0.7007	[-5.9015; 7.3029]
Jensterle,2015	GLP-1	Metformin	-1.4206	[-7.8187; 4.9775]

Number of studies: k = 10
Number of pairwise comparisons: m = 10
Number of treatments: n = 5
Number of designs: d = 5

Random effects model

Treatment estimate (sm = 'MD', comparison: other treatments vs 'Placebo'):

	MD	95%-CI	z	p-value
GLP-1	-4.6936	[-6.8644; -2.5229]	-4.24	< 0.0001
GLP-1 + Metformin	-5.3943	[-12.3442; 1.5555]	-1.52	0.1282
Metformin	-3.2730	[-10.0294; 3.4833]	-0.95	0.3424
Myoinositol + Folic acid	-4.9360	[-12.2333; 2.3613]	-1.33	0.1849
Placebo	.	.	.	.

Quantifying heterogeneity / inconsistency:
tau^2 = 1.9852; tau = 1.4090; I^2 = 56.8% [0.0%; 81.4%]

Tests of heterogeneity (within designs) and inconsistency (between designs):

	Q	d.f.	p-value
Total	13.88	6	0.0310
Within designs	12.84	5	0.0249
Between designs	1.04	1	0.3080

Details of network meta-analysis methods:
- Frequentist graph-theoretical approach
- DerSimonian-Laird estimator for tau^2
- Calculation of I^2 based on Q

[[8]]
Original data:

	treat1	treat2	TE	seTE
Wen,2023	GLP-1 + Metformin	Metformin	-0.3500	0.5100
Xing,2022	GLP-1 + Metformin	Metformin	-3.9000	0.9300
Elkind-Hirsch,2022	GLP-1	Placebo	-4.0000	0.9600
Ma,2021	GLP-1 + Metformin	Metformin	-2.9700	3.5800
Soldat-Stanković,2022	Metformin	Myoinositol + Folic acid	1.6900	4.2400
Frøssing,2018	GLP-1	Placebo	-5.2000	0.3700
Jensterle,2017	GLP-1	GLP-1 + Metformin	-2.0000	1.8900
Zheng,2017	GLP-1	Metformin	-3.1600	3.9100
Jensterle,2016	GLP-1	GLP-1 + Metformin	6.5000	7.2100
Jensterle,2015	GLP-1	Metformin	-1.8000	7.3600

Number of treatment arms (by study):

	narms
Wen,2023	2
Xing,2022	2
Elkind-Hirsch,2022	2
Ma,2021	2
Soldat-Stanković,2022	2
Frøssing,2018	2
Jensterle,2017	2
Zheng,2017	2
Jensterle,2016	2
Jensterle,2015	2

Results (random effects model):

	treat1	treat2	MD	95%-CI
Wen,2023	GLP-1 + Metformin	Metformin	-1.9800	[-4.0437; 0.0836]
Xing,2022	GLP-1 + Metformin	Metformin	-1.9800	[-4.0437; 0.0836]
Elkind-Hirsch,2022	GLP-1	Placebo	-4.6918	[-6.8855; -2.4980]
Ma,2021	GLP-1 + Metformin	Metformin	-1.9800	[-4.0437; 0.0836]
Soldat-Stanković,2022	Metformin	Myoinositol + Folic acid	1.6900	[-7.0783; 10.4583]
Frøssing,2018	GLP-1	Placebo	-4.6918	[-6.8855; -2.4980]
Jensterle,2017	GLP-1	GLP-1 + Metformin	-1.1060	[-4.9051; 2.6931]
Zheng,2017	GLP-1	Metformin	-3.0860	[-7.1271; 0.9550]
Jensterle,2016	GLP-1	GLP-1 + Metformin	-1.1060	[-4.9051; 2.6931]
Jensterle,2015	GLP-1	Metformin	-3.0860	[-7.1271; 0.9550]

Number of studies: k = 10
Number of pairwise comparisons: m = 10
Number of treatments: n = 5
Number of designs: d = 5

Random effects model

Treatment estimate (sm = 'MD', comparison: other treatments vs 'Placebo'):

	MD	95%-CI	z	p-value
GLP-1	-4.6918	[-6.8855; -2.4980]	-4.19	< 0.0001
GLP-1 + Metformin	-3.5858	[-7.9728; 0.8012]	-1.60	0.1092
Metformin	-1.6057	[-6.2038; 2.9924]	-0.68	0.4937
Myoinositol + Folic acid	-3.2957	[-13.1965; 6.6050]	-0.65	0.5141
Placebo	.	.	.	.

Quantifying heterogeneity / inconsistency:
tau^2 = 2.0363; tau = 1.4270; I^2 = 57.6% [1.8%; 81.7%]

Tests of heterogeneity (within designs) and inconsistency (between designs):

	Q	d.f.	p-value
Total	14.14	6	0.0281
Within designs	14.14	5	0.0148
Between designs	0.00	1	0.9577

Details of network meta-analysis methods:
- Frequentist graph-theoretical approach
- DerSimonian-Laird estimator for tau^2
- Calculation of I^2 based on Q

[[9]]
Original data:

	treat1	treat2	TE	seTE
Wen,2023	GLP-1 + Metformin	Metformin	-0.3500	0.5100
Xing,2022	GLP-1 + Metformin	Metformin	-3.9000	0.9300
Elkind-Hirsch,2022	GLP-1	Placebo	-4.0000	0.9600
Ma,2021	GLP-1 + Metformin	Metformin	-2.9700	3.5800
Soldat-Stanković,2022	Metformin	Myoinositol + Folic acid	1.6900	4.2400
Frøssing,2018	GLP-1	Placebo	-5.2000	0.3700
Jensterle,2017	GLP-1	GLP-1 + Metformin	-2.0000	1.8900
Ravn,2022	Metformin	Myoinositol + Folic acid	1.6600	0.4600
Jensterle,2016	GLP-1	GLP-1 + Metformin	6.5000	7.2100
Jensterle,2015	GLP-1	Metformin	-1.8000	7.3600

Number of treatment arms (by study):

	narms
Wen,2023	2
Xing,2022	2
Elkind-Hirsch,2022	2

Ma,2021 2
Soldat-Stanković,2022 2
Frøssing,2018 2
Jensterle,2017 2
Ravn,2022 2
Jensterle,2016 2
Jensterle,2015 2

Results (random effects model):

	treat1	treat2	MD	95%-CI
Wen,2023	GLP-1 + Metformin	Metformin	-1.9758	[-4.0860; 0.1344]
Xing,2022	GLP-1 + Metformin	Metformin	-1.9758	[-4.0860; 0.1344]
Elkind-Hirsch,2022	GLP-1	Placebo	-4.6916	[-6.8869; -2.4963]
Ma,2021	GLP-1 + Metformin	Metformin	-1.9758	[-4.0860; 0.1344]
Soldat-Stanković,2022	Metformin	Myoinositol + Folic acid	1.6630	[-1.1252; 4.4513]
Frøssing,2018	GLP-1	Placebo	-4.6916	[-6.8869; -2.4963]
Jensterle,2017	GLP-1	GLP-1 + Metformin	-1.0860	[-5.3216; 3.1496]
Ravn,2022	Metformin	Myoinositol + Folic acid	1.6630	[-1.1252; 4.4513]
Jensterle,2016	GLP-1	GLP-1 + Metformin	-1.0860	[-5.3216; 3.1496]
Jensterle,2015	GLP-1	Metformin	-3.0618	[-7.7152; 1.5916]

Number of studies: k = 10
Number of pairwise comparisons: m = 10
Number of treatments: n = 5
Number of designs: d = 5

Random effects model

Treatment estimate (sm = 'MD', comparison: other treatments vs 'Placebo'):

	MD	95%-CI	z	p-value
GLP-1	-4.6916	[-6.8869; -2.4963]	-4.19	< 0.0001
GLP-1 + Metformin	-3.6056	[-8.3763; 1.1651]	-1.48	0.1385
Metformin	-1.6298	[-6.7751; 3.5154]	-0.62	0.5347
Myoinositol + Folic acid	-3.2928	[-9.1450; 2.5593]	-1.10	0.2701
Placebo	.	.	.	.

Quantifying heterogeneity / inconsistency:
tau^2 = 2.0398; tau = 1.4282; I^2 = 57.5% [1.6%; 81.7%]

Tests of heterogeneity (within designs) and inconsistency (between designs):

	Q	d.f.	p-value
Total	14.12	6	0.0283
Within designs	14.11	5	0.0149

Between designs 0.01 1 0.9107

Details of network meta-analysis methods:
- Frequentist graph-theoretical approach
- DerSimonian-Laird estimator for tau^2
- Calculation of I^2 based on Q

[[10]]
Original data:

	treat1	treat2	TE	seTE
Wen,2023	GLP-1 + Metformin	Metformin	-0.3500	0.5100
Xing,2022	GLP-1 + Metformin	Metformin	-3.9000	0.9300
Elkind-Hirsch,2022	GLP-1	Placebo	-4.0000	0.9600
Ma,2021	GLP-1 + Metformin	Metformin	-2.9700	3.5800
Soldat-Stanković,2022	Metformin	Myoinositol + Folic acid	1.6900	4.2400
Frøssing,2018	GLP-1	Placebo	-5.2000	0.3700
Jensterle,2017	GLP-1	GLP-1 + Metformin	-2.0000	1.8900
Ravn,2022	Metformin	Myoinositol + Folic acid	1.6600	0.4600
Zheng,2017	GLP-1	Metformin	-3.1600	3.9100
Jensterle,2015	GLP-1	Metformin	-1.8000	7.3600

Number of treatment arms (by study):

	narms
Wen,2023	2
Xing,2022	2
Elkind-Hirsch,2022	2
Ma,2021	2
Soldat-Stanković,2022	2
Frøssing,2018	2
Jensterle,2017	2
Ravn,2022	2
Zheng,2017	2
Jensterle,2015	2

Results (random effects model):

	treat1	treat2	MD	95%-CI
Wen,2023	GLP-1 + Metformin	Metformin	-1.9027	[-3.8314; 0.0260]
Xing,2022	GLP-1 + Metformin	Metformin	-1.9027	[-3.8314; 0.0260]
Elkind-Hirsch,2022	GLP-1	Placebo	-4.7066	[-6.7332; -2.6801]
Ma,2021	GLP-1 + Metformin	Metformin	-1.9027	[-3.8314; 0.0260]
Soldat-Stanković,2022	Metformin	Myoinositol + Folic acid	1.6626	[-0.9112; 4.2364]
Frøssing,2018	GLP-1	Placebo	-4.7066	[-6.7332; -2.6801]

Jensterle,2017 GLP-1 GLP-1 + Metformin -1.6954 [-5.5267; 2.1360]
Ravn,2022 Metformin Myoinositol + Folic acid 1.6626 [-0.9112; 4.2364]
Zheng,2017 GLP-1 Metformin -3.5981 [-7.6305; 0.4343]
Jensterle,2015 GLP-1 Metformin -3.5981 [-7.6305; 0.4343]

Number of studies: k = 10
Number of pairwise comparisons: m = 10
Number of treatments: n = 5
Number of designs: d = 5

Random effects model

Treatment estimate (sm = 'MD', comparison: other treatments vs 'Placebo'):

	MD	95%-CI	z	p-value
GLP-1	-4.7066	[-6.7332; -2.6801]	-4.55	< 0.0001
GLP-1 + Metformin	-3.0112	[-7.3455; 1.3231]	-1.36	0.1733
Metformin	-1.1086	[-5.6216; 3.4045]	-0.48	0.6302
Myoinositol + Folic acid	-2.7712	[-7.9665; 2.4242]	-1.05	0.2958
Placebo	.	.	.	.

Quantifying heterogeneity / inconsistency:
tau^2 = 1.6787; tau = 1.2956; I^2 = 53.3% [0.0%; 80.1%]

Tests of heterogeneity (within designs) and inconsistency (between designs):

	Q	d.f.	p-value
Total	12.85	6	0.0456
Within designs	12.84	5	0.0249
Between designs	0.01	1	0.9321

Details of network meta-analysis methods:
- Frequentist graph-theoretical approach
- DerSimonian-Laird estimator for tau^2
- Calculation of I^2 based on Q

[[11]]
Original data:

	treat1	treat2	TE	seTE
Wen,2023	GLP-1 + Metformin	Metformin	-0.3500	0.5100
Xing,2022	GLP-1 + Metformin	Metformin	-3.9000	0.9300
Elkind-Hirsch,2022	GLP-1	Placebo	-4.0000	0.9600
Ma,2021	GLP-1 + Metformin	Metformin	-2.9700	3.5800
Soldat-Stanković,2022	Metformin	Myoinositol + Folic acid	1.6900	4.2400
Frøssing,2018	GLP-1	Placebo	-5.2000	0.3700

Jensterle,2017	GLP-1	GLP-1 + Metformin	-2.0000	1.8900
Ravn,2022	Metformin	Myoinositol + Folic acid	1.6600	0.4600
Zheng,2017	GLP-1	Metformin	-3.1600	3.9100
Jensterle,2016	GLP-1	GLP-1 + Metformin	6.5000	7.2100

Number of treatment arms (by study):

	narms
Wen,2023	2
Xing,2022	2
Elkind-Hirsch,2022	2
Ma,2021	2
Soldat-Stanković,2022	2
Frøssing,2018	2
Jensterle,2017	2
Ravn,2022	2
Zheng,2017	2
Jensterle,2016	2

Results (random effects model):

	treat1	treat2	MD	95%-CI
Wen,2023	GLP-1 + Metformin	Metformin	-1.9958	[-4.0556; 0.0641]
Xing,2022	GLP-1 + Metformin	Metformin	-1.9958	[-4.0556; 0.0641]
Elkind-Hirsch,2022	GLP-1	Placebo	-4.6933	[-6.8685; -2.5180]
Ma,2021	GLP-1 + Metformin	Metformin	-1.9958	[-4.0556; 0.0641]
Soldat-Stanković,2022	Metformin	Myoinositol + Folic acid	1.6630	[-1.1000; 4.4259]
Frøssing,2018	GLP-1	Placebo	-4.6933	[-6.8685; -2.5180]
Jensterle,2017	GLP-1	GLP-1 + Metformin	-1.1960	[-5.0976; 2.7055]
Ravn,2022	Metformin	Myoinositol + Folic acid	1.6630	[-1.1000; 4.4259]
Zheng,2017	GLP-1	Metformin	-3.1918	[-7.3807; 0.9972]
Jensterle,2016	GLP-1	GLP-1 + Metformin	-1.1960	[-5.0976; 2.7055]

Number of studies: k = 10
Number of pairwise comparisons: m = 10
Number of treatments: n = 5
Number of designs: d = 5

Random effects model

Treatment estimate (sm = 'MD', comparison: other treatments vs 'Placebo'):

	MD	95%-CI	z	p-value
GLP-1	-4.6933	[-6.8685; -2.5180]	-4.23	< 0.0001
GLP-1 + Metformin	-3.4972	[-7.9642; 0.9697]	-1.53	0.1249
Metformin	-1.5015	[-6.2215; 3.2186]	-0.62	0.5330

Myoinositol + Folic acid -3.1645 [-8.6337; 2.3048] -1.13 0.2568
Placebo . . . .

Quantifying heterogeneity / inconsistency:
tau^2 = 1.9952; tau = 1.4125; I^2 = 57.5% [1.6%; 81.7%]

Tests of heterogeneity (within designs) and inconsistency (between designs):

	Q	d.f.	p-value
Total	14.13	6	0.0283
Within designs	14.11	5	0.0149
Between designs	0.01	1	0.9068

Details of network meta-analysis methods:
- Frequentist graph-theoretical approach
- DerSimonian-Laird estimator for tau^2
- Calculation of I^2 based on Q

=== Heterogeneity & Inconsistency ===
[1] 0.504997
Q statistics to assess homogeneity / consistency

Design-specific decomposition of within-designs Q statistic

Design	Q	df	p-value
GLP-1 + Metformin vs Metformin	11.45	2	0.0033
Placebo vs GLP-1	1.36	1	0.2435
GLP-1 vs GLP-1 + Metformin	1.30	1	0.2541
GLP-1 vs Metformin	0.03	1	0.8704
Metformin vs Myoinositol + Folic acid	0.00	1	0.9944

Between-designs Q statistic after detaching of single designs
(influential designs have p-value markedly different from 0.9577)

Detached design	Q	df	p-value
GLP-1 + Metformin vs Metformin	0.00	0	--
GLP-1 vs GLP-1 + Metformin	0.00	0	--
GLP-1 vs Metformin	0.00	0	--

Q statistic to assess consistency under the assumption of
a full design-by-treatment interaction random effects model

HOMA-IR Change

=== Sensitivity Analysis Results ===

=== Excluding High Risk of Bias (rob != 3) ===
❌ Not enough data to run network meta-analysis for low-risk studies.

=== Leave-One-Out Analysis ===
[[1]]
Original data:

	treat1	treat2	TE	seTE
Xing,2022	GLP-1 + Metformin	Metformin	-0.5600	0.7800
Elkind-Hirsch,2022	GLP-1	Placebo	-0.8000	0.3000
Ma,2021	GLP-1 + Metformin	Metformin	-0.9000	0.9300
Soldat-Stanković,2022	Metformin	Myoinositol + Folic acid	0.4600	0.6100
Tao,2021	GLP-1	Metformin	1.3600	0.0800
Zheng,2019	GLP-1	Metformin	-0.6100	0.3900
Shokrpour,2019	Metformin	Myoinositol + Folic acid	0.3000	0.2700
Jensterle,2017	GLP-1	GLP-1 + Metformin	1.4000	1.2100
Zahra,2016	Metformin	Placebo	0.9000	0.8200
Ravn,2022	Metformin	Myoinositol + Folic acid	-14.0700	7.3100
Zheng,2017	GLP-1	Metformin	-0.6100	0.5000
Jensterle,2016	GLP-1	GLP-1 + Metformin	-0.3000	0.6300
Jensterle,2015	GLP-1	Metformin	-0.2000	1.2200

Number of treatment arms (by study):

	narms
Xing,2022	2
Elkind-Hirsch,2022	2
Ma,2021	2
Soldat-Stanković,2022	2
Tao,2021	2
Zheng,2019	2
Shokrpour,2019	2
Jensterle,2017	2
Zahra,2016	2
Ravn,2022	2
Zheng,2017	2
Jensterle,2016	2
Jensterle,2015	2

Results (random effects model):

```
                         treat1                  treat2      MD           95%-CI
Xing,2022            GLP-1 + Metformin              Metformin -0.6286 [-2.2097; 0.9524]
Elkind-Hirsch,2022              GLP-1                 Placebo -0.1537 [-2.0930; 1.7857]
Ma,2021              GLP-1 + Metformin              Metformin -0.6286 [-2.2097; 0.9524]
Soldat-Stanković,2022         Metformin Myoinositol + Folic acid 0.1520 [-1.6452; 1.9492]
Tao,2021                        GLP-1              Metformin -0.1703 [-1.3244; 0.9838]
Zheng,2019                      GLP-1              Metformin -0.1703 [-1.3244; 0.9838]
Shokrpour,2019              Metformin Myoinositol + Folic acid 0.1520 [-1.6452; 1.9492]
Jensterle,2017                  GLP-1       GLP-1 + Metformin 0.4583 [-1.1305; 2.0471]
Zahra,2016                  Metformin                 Placebo 0.0167 [-1.9752; 2.0085]
Ravn,2022                   Metformin Myoinositol + Folic acid 0.1520 [-1.6452; 1.9492]
Zheng,2017                      GLP-1              Metformin -0.1703 [-1.3244; 0.9838]
Jensterle,2016                  GLP-1       GLP-1 + Metformin 0.4583 [-1.1305; 2.0471]
Jensterle,2015                  GLP-1              Metformin -0.1703 [-1.3244; 0.9838]

Number of studies: k = 13
Number of pairwise comparisons: m = 13
Number of treatments: n = 5
Number of designs: d = 6

Random effects model

Treatment estimate (sm = 'MD', comparison: other treatments vs 'Placebo'):
                              MD           95%-CI     z p-value
GLP-1                    -0.1537 [-2.0930; 1.7857] -0.16 0.8766
GLP-1 + Metformin        -0.6120 [-3.0020; 1.7780] -0.50 0.6158
Metformin                 0.0167 [-1.9752; 2.0085] 0.02 0.9869
Myoinositol + Folic acid -0.1353 [-2.8181; 2.5475] -0.10 0.9212
Placebo                        .                .     .       .

Quantifying heterogeneity / inconsistency:
tau^2 = 1.4983; tau = 1.2240; I^2 = 85.4% [74.9%; 91.5%]

Tests of heterogeneity (within designs) and inconsistency (between designs):
                    Q d.f. p-value
Total           61.58    9 < 0.0001
Within designs 45.38     7 < 0.0001
Between designs 16.21    2  0.0003

Details of network meta-analysis methods:
- Frequentist graph-theoretical approach
- DerSimonian-Laird estimator for tau^2
- Calculation of I^2 based on Q
```

[[2]]
Original data:

	treat1	treat2	TE	seTE
Wen,2023	GLP-1 + Metformin	Metformin	-0.6700	0.1400
Elkind-Hirsch,2022	GLP-1	Placebo	-0.8000	0.3000
Ma,2021	GLP-1 + Metformin	Metformin	-0.9000	0.9300
Soldat-Stanković,2022	Metformin	Myoinositol + Folic acid	0.4600	0.6100
Tao,2021	GLP-1	Metformin	1.3600	0.0800
Zheng,2019	GLP-1	Metformin	-0.6100	0.3900
Shokrpour,2019	Metformin	Myoinositol + Folic acid	0.3000	0.2700
Jensterle,2017	GLP-1	GLP-1 + Metformin	1.4000	1.2100
Zahra,2016	Metformin	Placebo	0.9000	0.8200
Ravn,2022	Metformin	Myoinositol + Folic acid	-14.0700	7.3100
Zheng,2017	GLP-1	Metformin	-0.6100	0.5000
Jensterle,2016	GLP-1	GLP-1 + Metformin	-0.3000	0.6300
Jensterle,2015	GLP-1	Metformin	-0.2000	1.2200

Number of treatment arms (by study):

	narms
Wen,2023	2
Elkind-Hirsch,2022	2
Ma,2021	2
Soldat-Stanković,2022	2
Tao,2021	2
Zheng,2019	2
Shokrpour,2019	2
Jensterle,2017	2
Zahra,2016	2
Ravn,2022	2
Zheng,2017	2
Jensterle,2016	2
Jensterle,2015	2

Results (random effects model):

	treat1	treat2	MD	95%-CI
Wen,2023	GLP-1 + Metformin	Metformin	-0.6596	[-2.1226; 0.8033]
Elkind-Hirsch,2022	GLP-1	Placebo	-0.1616	[-2.0572; 1.7340]
Ma,2021	GLP-1 + Metformin	Metformin	-0.6596	[-2.1226; 0.8033]
Soldat-Stanković,2022	Metformin	Myoinositol + Folic acid	0.1612	[-1.5954; 1.9179]
Tao,2021	GLP-1	Metformin	-0.1772	[-1.2985; 0.9442]
Zheng,2019	GLP-1	Metformin	-0.1772	[-1.2985; 0.9442]
Shokrpour,2019	Metformin	Myoinositol + Folic acid	0.1612	[-1.5954; 1.9179]

```
Jensterle,2017              GLP-1      GLP-1 + Metformin 0.4825 [-1.0295; 1.9944]
Zahra,2016              Metformin              Placebo 0.0156 [-1.9328; 1.9640]
Ravn,2022               Metformin Myoinositol + Folic acid 0.1612 [-1.5954; 1.9179]
Zheng,2017                GLP-1             Metformin -0.1772 [-1.2985; 0.9442]
Jensterle,2016              GLP-1      GLP-1 + Metformin 0.4825 [-1.0295; 1.9944]
Jensterle,2015              GLP-1             Metformin -0.1772 [-1.2985; 0.9442]

Number of studies: k = 13
Number of pairwise comparisons: m = 13
Number of treatments: n = 5
Number of designs: d = 6

Random effects model

Treatment estimate (sm = 'MD', comparison: other treatments vs 'Placebo'):
                              MD          95%-CI     z p-value
GLP-1                    -0.1616 [-2.0572; 1.7340] -0.17 0.8673
GLP-1 + Metformin        -0.6440 [-2.9443; 1.6562] -0.55 0.5832
Metformin                 0.0156 [-1.9328; 1.9640] 0.02 0.9875
Myoinositol + Folic acid -0.1456 [-2.7690; 2.4778] -0.11 0.9134
Placebo                        .                .     .      .

Quantifying heterogeneity / inconsistency:
tau^2 = 1.4216; tau = 1.1923; I^2 = 86.4% [77.0%; 92.0%]

Tests of heterogeneity (within designs) and inconsistency (between designs):
                    Q d.f. p-value
Total           66.34    9 < 0.0001
Within designs  45.36    7 < 0.0001
Between designs 20.98    2 < 0.0001

Details of network meta-analysis methods:
- Frequentist graph-theoretical approach
- DerSimonian-Laird estimator for tau^2
- Calculation of I^2 based on Q

[[3]]
Original data:

                              treat1                   treat2      TE   seTE
Wen,2023                GLP-1 + Metformin               Metformin -0.6700 0.1400
Xing,2022               GLP-1 + Metformin               Metformin -0.5600 0.7800
Ma,2021                 GLP-1 + Metformin               Metformin -0.9000 0.9300
Soldat-Stanković,2022          Metformin Myoinositol + Folic acid 0.4600 0.6100
```

Tao,2021	GLP-1	Metformin	1.3600	0.0800
Zheng,2019	GLP-1	Metformin	-0.6100	0.3900
Shokrpour,2019	Metformin	Myoinositol + Folic acid	0.3000	0.2700
Jensterle,2017	GLP-1	GLP-1 + Metformin	1.4000	1.2100
Zahra,2016	Metformin	Placebo	0.9000	0.8200
Ravn,2022	Metformin	Myoinositol + Folic acid	-14.0700	7.3100
Zheng,2017	GLP-1	Metformin	-0.6100	0.5000
Jensterle,2016	GLP-1	GLP-1 + Metformin	-0.3000	0.6300
Jensterle,2015	GLP-1	Metformin	-0.2000	1.2200

Number of treatment arms (by study):

	narms
Wen,2023	2
Xing,2022	2
Ma,2021	2
Soldat-Stanković,2022	2
Tao,2021	2
Zheng,2019	2
Shokrpour,2019	2
Jensterle,2017	2
Zahra,2016	2
Ravn,2022	2
Zheng,2017	2
Jensterle,2016	2
Jensterle,2015	2

Results (random effects model):

	treat1	treat2	MD	95%-CI
Wen,2023	GLP-1 + Metformin	Metformin	-0.5755	[-1.7844; 0.6333]
Xing,2022	GLP-1 + Metformin	Metformin	-0.5755	[-1.7844; 0.6333]
Ma,2021	GLP-1 + Metformin	Metformin	-0.5755	[-1.7844; 0.6333]
Soldat-Stanković,2022	Metformin	Myoinositol + Folic acid	0.1914	[-1.4257; 1.8085]
Tao,2021	GLP-1	Metformin	-0.0151	[-1.0870; 1.0567]
Zheng,2019	GLP-1	Metformin	-0.0151	[-1.0870; 1.0567]
Shokrpour,2019	Metformin	Myoinositol + Folic acid	0.1914	[-1.4257; 1.8085]
Jensterle,2017	GLP-1	GLP-1 + Metformin	0.5604	[-0.7838; 1.9046]
Zahra,2016	Metformin	Placebo	0.9000	[-1.7619; 3.5619]
Ravn,2022	Metformin	Myoinositol + Folic acid	0.1914	[-1.4257; 1.8085]
Zheng,2017	GLP-1	Metformin	-0.0151	[-1.0870; 1.0567]
Jensterle,2016	GLP-1	GLP-1 + Metformin	0.5604	[-0.7838; 1.9046]
Jensterle,2015	GLP-1	Metformin	-0.0151	[-1.0870; 1.0567]

Number of studies: k = 13

```
Number of pairwise comparisons: m = 13
Number of treatments: n = 5
Number of designs: d = 5

Random effects model

Treatment estimate (sm = 'MD', comparison: other treatments vs 'Placebo'):
                              MD            95%-CI    z p-value
GLP-1                     0.8849 [-1.9848; 3.7545] 0.60 0.5456
GLP-1 + Metformin         0.3245 [-2.5991; 3.2480] 0.22 0.8278
Metformin                 0.9000 [-1.7619; 3.5619] 0.66 0.5075
Myoinositol + Folic acid  0.7086 [-2.4060; 3.8232] 0.45 0.6557
Placebo                        .                 .    .       .

Quantifying heterogeneity / inconsistency:
tau^2 = 1.1722; tau = 1.0827; I^2 = 83.8% [71.7%; 90.7%]

Tests of heterogeneity (within designs) and inconsistency (between designs):
                    Q d.f.  p-value
Total           55.42    9 < 0.0001
Within designs  45.38    8 < 0.0001
Between designs 10.03    1   0.0015

Details of network meta-analysis methods:
- Frequentist graph-theoretical approach
- DerSimonian-Laird estimator for tau^2
- Calculation of I^2 based on Q

[[4]]
Original data:

                                   treat1                   treat2       TE   seTE
Wen,2023                GLP-1 + Metformin                Metformin  -0.6700 0.1400
Xing,2022               GLP-1 + Metformin                Metformin  -0.5600 0.7800
Elkind-Hirsch,2022                  GLP-1                  Placebo  -0.8000 0.3000
Soldat-Stanković,2022           Metformin Myoinositol + Folic acid   0.4600 0.6100
Tao,2021                            GLP-1                Metformin   1.3600 0.0800
Zheng,2019                          GLP-1                Metformin  -0.6100 0.3900
Shokrpour,2019                  Metformin Myoinositol + Folic acid   0.3000 0.2700
Jensterle,2017                      GLP-1        GLP-1 + Metformin   1.4000 1.2100
Zahra,2016                      Metformin                  Placebo   0.9000 0.8200
Ravn,2022                       Metformin Myoinositol + Folic acid -14.0700 7.3100
Zheng,2017                          GLP-1                Metformin  -0.6100 0.5000
Jensterle,2016                      GLP-1        GLP-1 + Metformin  -0.3000 0.6300
```

Jensterle,2015 GLP-1 Metformin -0.2000 1.2200

Number of treatment arms (by study):

	narms
Wen,2023	2
Xing,2022	2
Elkind-Hirsch,2022	2
Soldat-Stanković,2022	2
Tao,2021	2
Zheng,2019	2
Shokrpour,2019	2
Jensterle,2017	2
Zahra,2016	2
Ravn,2022	2
Zheng,2017	2
Jensterle,2016	2
Jensterle,2015	2

Results (random effects model):

	treat1	treat2	MD	95%-CI
Wen,2023	GLP-1 + Metformin	Metformin	-0.5749	[-2.0021; 0.8522]
Xing,2022	GLP-1 + Metformin	Metformin	-0.5749	[-2.0021; 0.8522]
Elkind-Hirsch,2022	GLP-1	Placebo	-0.1545	[-2.0309; 1.7219]
Soldat-Stanković,2022	Metformin	Myoinositol + Folic acid	0.1653	[-1.5735; 1.9040]
Tao,2021	GLP-1	Metformin	-0.1546	[-1.2631; 0.9538]
Zheng,2019	GLP-1	Metformin	-0.1546	[-1.2631; 0.9538]
Shokrpour,2019	Metformin	Myoinositol + Folic acid	0.1653	[-1.5735; 1.9040]
Jensterle,2017	GLP-1	GLP-1 + Metformin	0.4203	[-1.0665; 1.9071]
Zahra,2016	Metformin	Placebo	0.0002	[-1.9294; 1.9297]
Ravn,2022	Metformin	Myoinositol + Folic acid	0.1653	[-1.5735; 1.9040]
Zheng,2017	GLP-1	Metformin	-0.1546	[-1.2631; 0.9538]
Jensterle,2016	GLP-1	GLP-1 + Metformin	0.4203	[-1.0665; 1.9071]
Jensterle,2015	GLP-1	Metformin	-0.1546	[-1.2631; 0.9538]

Number of studies: k = 13
Number of pairwise comparisons: m = 13
Number of treatments: n = 5
Number of designs: d = 6

Random effects model

Treatment estimate (sm = 'MD', comparison: other treatments vs 'Placebo'):

MD 95%-CI z p-value

```
GLP-1                      -0.1545 [-2.0309; 1.7219] -0.16 0.8718
GLP-1 + Metformin          -0.5748 [-2.8432; 1.6936] -0.50 0.6195
Metformin                   0.0002 [-1.9294; 1.9297]  0.00 0.9999
Myoinositol + Folic acid   -0.1651 [-2.7625; 2.4323] -0.12 0.9009
Placebo                          .                  .     .      .

Quantifying heterogeneity / inconsistency:
tau^2 = 1.3883; tau = 1.1782; I^2 = 86.4% [76.9%; 92.0%]

Tests of heterogeneity (within designs) and inconsistency (between designs):
                    Q d.f. p-value
Total           66.21    9 < 0.0001
Within designs  45.32    7 < 0.0001
Between designs 20.89    2 < 0.0001

Details of network meta-analysis methods:
- Frequentist graph-theoretical approach
- DerSimonian-Laird estimator for tau^2
- Calculation of I^2 based on Q

[[5]]
Original data:

                              treat1                   treat2        TE   seTE

Elkind-Hirsch,2022            GLP-1                   Placebo -0.8000 0.3000
Ma,2021             GLP-1 + Metformin               Metformin -0.9000 0.9300
Tao,2021                      GLP-1                 Metformin  1.3600 0.0800
Zheng,2019                    GLP-1                 Metformin -0.6100 0.3900
Shokrpour,2019            Metformin Myoinositol + Folic acid 0.3000 0.270
Jensterle,2017                GLP-1       GLP-1 + Metformin 1.4000 1.2100
Zahra,2016                Metformin                 Placebo  0.9000 0.8200
Ravn,2022                 Metformin Myoinositol + Folic acid -14.0700 7.3100
Zheng,2017                    GLP-1                 Metformin -0.6100 0.5000
Jensterle,2016                GLP-1        GLP-1 + Metformin -0.3000 0.6300
Jensterle,2015                GLP-1                 Metformin -0.2000 1.2200
Wen,2023             GLP-1 + Metformin              Metformin -0.6700 0.1400
Xing,2022            GLP-1 + Metformin              Metformin -0.5600 0.7800
```

Number of treatment arms (by study):

	narms
Wen,2023	2
Xing,2022	2
Elkind-Hirsch,2022	2
Ma,2021	2

Tao,2021 2
Zheng,2019 2
Shokrpour,2019 2
Jensterle,2017 2
Zahra,2016 2
Ravn,2022 2
Zheng,2017 2
Jensterle,2016 2
Jensterle,2015 2

Results (random effects model):

	treat1	treat2	MD	95%-CI
Wen,2023	GLP-1 + Metformin	Metformin	-0.6402	[-1.9551; 0.6747]
Xing,2022	GLP-1 + Metformin	Metformin	-0.6402	[-1.9551; 0.6747]
Elkind-Hirsch,2022	GLP-1	Placebo	-0.1562	[-2.0807; 1.7682]
Ma,2021	GLP-1 + Metformin	Metformin	-0.6402	[-1.9551; 0.6747]
Tao,2021	GLP-1	Metformin	-0.1730	[-1.2976; 0.9516]
Zheng,2019	GLP-1	Metformin	-0.1730	[-1.2976; 0.9516]
Shokrpour,2019	Metformin	Myoinositol + Folic acid	-0.0941	[-2.4994; 2.3112]
Jensterle,2017	GLP-1	GLP-1 + Metformin	0.4672	[-0.9789; 1.9133]
Zahra,2016	Metformin	Placebo	0.0168	[-1.9586; 1.9921]
Ravn,2022	Metformin	Myoinositol + Folic acid	-0.0941	[-2.4994; 2.3112]
Zheng,2017	GLP-1	Metformin	-0.1730	[-1.2976; 0.9516]
Jensterle,2016	GLP-1	GLP-1 + Metformin	0.4672	[-0.9789; 1.9133]
Jensterle,2015	GLP-1	Metformin	-0.1730	[-1.2976; 0.9516]

Number of studies: k = 13
Number of pairwise comparisons: m = 13
Number of treatments: n = 5
Number of designs: d = 6

Random effects model

Treatment estimate (sm = 'MD', comparison: other treatments vs 'Placebo'):

	MD	95%-CI	z	p-value
GLP-1	-0.1562	[-2.0807; 1.7682]	-0.16	0.8736
GLP-1 + Metformin	-0.6235	[-2.8837; 1.6368]	-0.54	0.5888
Metformin	0.0168	[-1.9586; 1.9921]	0.02	0.9867
Myoinositol + Folic acid	0.1109	[-3.0016; 3.2234]	0.07	0.9443
Placebo	.	.	.	.

Quantifying heterogeneity / inconsistency:
tau^2 = 1.4756; tau = 1.2148; I^2 = 86.4% [76.9%; 92.0%]

```
Tests of heterogeneity (within designs) and inconsistency (between designs):
                    Q d.f. p-value
Total           66.27    9 < 0.0001
Within designs 45.31     7 < 0.0001
Between designs 20.96    2 < 0.0001

Details of network meta-analysis methods:
- Frequentist graph-theoretical approach
- DerSimonian-Laird estimator for tau^2
- Calculation of I^2 based on Q

[[6]]
Original data:

                                treat1                  treat2       TE  seTE
Wen,2023              GLP-1 + Metformin               Metformin -0.6700 0.1400
Xing,2022             GLP-1 + Metformin               Metformin -0.5600 0.7800
Elkind-Hirsch,2022                GLP-1                 Placebo -0.8000 0.3000
Ma,2021               GLP-1 + Metformin               Metformin -0.9000 0.9300
Soldat-Stanković,2022         Metformin Myoinositol + Folic acid 0.4600 0.6100
Zheng,2019                        GLP-1               Metformin -0.6100 0.3900
Shokrpour,2019                Metformin Myoinositol + Folic acid 0.3000 0.2700
Jensterle,2017                    GLP-1       GLP-1 + Metformin 1.4000 1.2100
Zahra,2016                    Metformin                 Placebo 0.9000 0.8200
Ravn,2022                     Metformin Myoinositol + Folic acid -14.0700 7.3100
Zheng,2017                        GLP-1               Metformin -0.6100 0.5000
Jensterle,2016                    GLP-1       GLP-1 + Metformin -0.3000 0.6300
Jensterle,2015                    GLP-1               Metformin -0.2000 1.2200

Number of treatment arms (by study):
                      narms
Wen,2023                  2
Xing,2022                 2
Elkind-Hirsch,2022        2
Ma,2021                   2
Soldat-Stanković,2022     2
Zheng,2019                2
Shokrpour,2019            2
Jensterle,2017            2
Zahra,2016                2
Ravn,2022                 2
Zheng,2017                2
Jensterle,2016            2
```

Jensterle,2015 2

Results (random effects model):

	treat1	treat2	MD	95%-CI
Wen,2023	GLP-1 + Metformin	Metformin	-0.6758	[-0.9368; -0.4147]
Xing,2022	GLP-1 + Metformin	Metformin	-0.6758	[-0.9368; -0.4147]
Elkind-Hirsch,2022	GLP-1	Placebo	-0.6800	[-1.2353; -0.1248]
Ma,2021	GLP-1 + Metformin	Metformin	-0.6758	[-0.9368; -0.4147]
Soldat-Stanković,2022	Metformin	Myoinositol + Folic acid	0.3098	[-0.1738; 0.7934]
Zheng,2019	GLP-1	Metformin	-0.6839	[-1.1804; -0.1874]
Shokrpour,2019	Metformin	Myoinositol + Folic acid	0.3098	[-0.1738; 0.7934]
Jensterle,2017	GLP-1	GLP-1 + Metformin	-0.0081	[-0.5438; 0.5276]
Zahra,2016	Metformin	Placebo	0.0038	[-0.7009; 0.7086]
Ravn,2022	Metformin	Myoinositol + Folic acid	0.3098	[-0.1738; 0.7934]
Zheng,2017	GLP-1	Metformin	-0.6839	[-1.1804; -0.1874]
Jensterle,2016	GLP-1	GLP-1 + Metformin	-0.0081	[-0.5438; 0.5276]
Jensterle,2015	GLP-1	Metformin	-0.6839	[-1.1804; -0.1874]

Number of studies: k = 13
Number of pairwise comparisons: m = 13
Number of treatments: n = 5
Number of designs: d = 6

Random effects model

Treatment estimate (sm = 'MD', comparison: other treatments vs 'Placebo'):

	MD	95%-CI	z	p-value
GLP-1	-0.6800	[-1.2353; -0.1248]	-2.40	0.0164
GLP-1 + Metformin	-0.6719	[-1.4071; 0.0632]	-1.79	0.0732
Metformin	0.0038	[-0.7009; 0.7086]	0.01	0.9915
Myoinositol + Folic acid	-0.3060	[-1.1607; 0.5488]	-0.70	0.4829
Placebo	.	.	.	.

Quantifying heterogeneity / inconsistency:
tau^2 = 0; tau = 0; I^2 = 0% [0.0%; 62.4%]

Tests of heterogeneity (within designs) and inconsistency (between designs):

	Q	d.f.	p-value
Total	7.15	9	0.6213
Within designs	5.67	7	0.5786
Between designs	1.48	2	0.4771

Details of network meta-analysis methods:

- Frequentist graph-theoretical approach
- DerSimonian-Laird estimator for tau^2
- Calculation of I^2 based on Q

[[7]]
Original data:

	treat1	treat2	TE	seTE
Wen,2023	GLP-1 + Metformin	Metformin	-0.6700	0.1400
Xing,2022	GLP-1 + Metformin	Metformin	-0.5600	0.7800
Elkind-Hirsch,2022	GLP-1	Placebo	-0.8000	0.3000
Ma,2021	GLP-1 + Metformin	Metformin	-0.9000	0.9300
Soldat-Stanković,2022	Metformin	Myoinositol + Folic acid	0.4600	0.6100
Tao,2021	GLP-1	Metformin	1.3600	0.0800
Shokrpour,2019	Metformin	Myoinositol + Folic acid	0.3000	0.2700
Jensterle,2017	GLP-1	GLP-1 + Metformin	1.4000	1.2100
Zahra,2016	Metformin	Placebo	0.9000	0.8200
Ravn,2022	Metformin	Myoinositol + Folic acid	-14.0700	7.3100
Zheng,2017	GLP-1	Metformin	-0.6100	0.5000
Jensterle,2016	GLP-1	GLP-1 + Metformin	-0.3000	0.6300
Jensterle,2015	GLP-1	Metformin	-0.2000	1.2200

Number of treatment arms (by study):

Wen,2023	2
Xing,2022	2
Elkind-Hirsch,2022	2
Ma,2021	2
Soldat-Stanković,2022	
Tao,2021	2
Shokrpour,2019	2
Jensterle,2017	2
Zahra,2016	2
Ravn,2022	2
Zheng,2017	2
Jensterle,2016	2
Jensterle,2015	2

narms

2

Results (random effects model):

	treat1	treat2	MD	95%-CI
Wen,2023	GLP-1 + Metformin	Metformin	-0.5847	[-1.7878; 0.6183]
Xing,2022	GLP-1 + Metformin	Metformin	-0.5847	[-1.7878; 0.6183]
Elkind-Hirsch,2022	GLP-1	Placebo	-0.1312	[-1.8681; 1.6056]

Ma,2021 GLP-1 + Metformin Metformin -0.5847 [-1.7878; 0.6183]
Soldat-Stanković,2022 Metformin Myoinositol + Folic acid 0.1958 [-1.3998; 1.7914]
Tao,2021 GLP-1 Metformin -0.0447 [-1.1795; 1.0900]
Shokrpour,2019 Metformin Myoinositol + Folic acid 0.1958 [-1.3998; 1.7914]
Jensterle,2017 GLP-1 GLP-1 + Metformin 0.5400 [-0.8155; 1.8955]
Zahra,2016 Metformin Placebo -0.0865 [-1.8931; 1.7201]
Ravn,2022 Metformin Myoinositol + Folic acid 0.1958 [-1.3998; 1.7914]
Zheng,2017 GLP-1 Metformin -0.0447 [-1.1795; 1.0900]
Jensterle,2016 GLP-1 GLP-1 + Metformin 0.5400 [-0.8155; 1.8955]
Jensterle,2015 GLP-1 Metformin -0.0447 [-1.1795; 1.0900]

Number of studies: k = 13
Number of pairwise comparisons: m = 13
Number of treatments: n = 5
Number of designs: d = 6

Random effects model

Treatment estimate (sm = 'MD', comparison: other treatments vs 'Placebo'):

	MD	95%-CI	z	p-value
GLP-1	-0.1312	[-1.8681; 1.6056]	-0.15	0.8823
GLP-1 + Metformin	-0.6712	[-2.7148; 1.3723]	-0.64	0.5197
Metformin	-0.0865	[-1.8931; 1.7201]	-0.09	0.9252
Myoinositol + Folic acid	-0.2823	[-2.6926; 2.1280]	-0.23	0.8184
Placebo	.	.	.	.

Quantifying heterogeneity / inconsistency:
tau^2 = 1.1358; tau = 1.0657; I^2 = 79.8% [63.6%; 88.8%]

Tests of heterogeneity (within designs) and inconsistency (between designs):

	Q	d.f.	p-value
Total	44.56	9	< 0.0001
Within designs	22.23	7	0.0023
Between designs	22.34	2	< 0.0001

Details of network meta-analysis methods:
- Frequentist graph-theoretical approach
- DerSimonian-Laird estimator for tau^2
- Calculation of I^2 based on Q

[[8]]
Original data:

treat1 treat2 TE seTE

Wen,2023	GLP-1 + Metformin	Metformin	-0.6700	0.1400
Xing,2022	GLP-1 + Metformin	Metformin	-0.5600	0.7800
Elkind-Hirsch,2022	GLP-1	Placebo	-0.8000	0.3000
Ma,2021	GLP-1 + Metformin	Metformin	-0.9000	0.9300
Soldat-Stanković,2022	Metformin	Myoinositol + Folic acid	0.4600	0.6100
Tao,2021	GLP-1	Metformin	1.3600	0.0800
Zheng,2019	GLP-1	Metformin	-0.6100	0.3900
Jensterle,2017	GLP-1	GLP-1 + Metformin	1.4000	1.2100
Zahra,2016	Metformin	Placebo	0.9000	0.8200
Ravn,2022	Metformin	Myoinositol + Folic acid	-14.0700	7.3100
Zheng,2017	GLP-1	Metformin	-0.6100	0.5000
Jensterle,2016	GLP-1	GLP-1 + Metformin	-0.3000	0.6300
Jensterle,2015	GLP-1	Metformin	-0.2000	1.2200

Number of treatment arms (by study):

	narms
Wen,2023	2
Xing,2022	2
Elkind-Hirsch,2022	2
Ma,2021	2
Soldat-Stanković,2022	2
Tao,2021	2
Zheng,2019	2
Jensterle,2017	2
Zahra,2016	2
Ravn,2022	2
Zheng,2017	2
Jensterle,2016	2
Jensterle,2015	2

Results (random effects model):

	treat1	treat2	MD	95%-CI
Wen,2023	GLP-1 + Metformin	Metformin	-0.6403	[-1.9557; 0.6752]
Xing,2022	GLP-1 + Metformin	Metformin	-0.6403	[-1.9557; 0.6752]
Elkind-Hirsch,2022	GLP-1	Placebo	-0.1562	[-2.0815; 1.7692]
Ma,2021	GLP-1 + Metformin	Metformin	-0.6403	[-1.9557; 0.6752]
Soldat-Stanković,2022	Metformin	Myoinositol + Folic acid	-0.0134	[-2.6350; 2.6082]
Tao,2021	GLP-1	Metformin	-0.1731	[-1.2982; 0.9520]
Zheng,2019	GLP-1	Metformin	-0.1731	[-1.2982; 0.9520]
Jensterle,2017	GLP-1	GLP-1 + Metformin	0.4672	[-0.9794; 1.9139]
Zahra,2016	Metformin	Placebo	0.0169	[-1.9593; 1.9931]
Ravn,2022	Metformin	Myoinositol + Folic acid	-0.0134	[-2.6350; 2.6082]
Zheng,2017	GLP-1	Metformin	-0.1731	[-1.2982; 0.9520]

```
Jensterle,2016                GLP-1       GLP-1 + Metformin 0.4672 [-0.9794; 1.9139]
Jensterle,2015                GLP-1             Metformin -0.1731 [-1.2982; 0.9520]

Number of studies: k = 13
Number of pairwise comparisons: m = 13
Number of treatments: n = 5
Number of designs: d = 6

Random effects model

Treatment estimate (sm = 'MD', comparison: other treatments vs 'Placebo'):
                              MD            95%-CI     z p-value
GLP-1                     -0.1562 [-2.0815; 1.7692] -0.16 0.8737
GLP-1 + Metformin         -0.6234 [-2.8847; 1.6379] -0.54 0.5890
Metformin                  0.0169 [-1.9593; 1.9931] 0.02 0.9866
Myoinositol + Folic acid 0.0303 [-3.2527; 3.3134] 0.02 0.9856
Placebo                        .                 .     .       .

Quantifying heterogeneity / inconsistency:
tau^2 = 1.4773; tau = 1.2154; I^2 = 86.4% [77.0%; 92.0%]

Tests of heterogeneity (within designs) and inconsistency (between designs):
                    Q d.f. p-value
Total           66.33    9 < 0.0001
Within designs 45.37     7 < 0.0001
Between designs 20.96    2 < 0.0001

Details of network meta-analysis methods:
- Frequentist graph-theoretical approach
- DerSimonian-Laird estimator for tau^2
- Calculation of I^2 based on Q

[[9]]
Original data:

                              treat1                 treat2       TE  seTE
Wen,2023              GLP-1 + Metformin              Metformin -0.6700 0.1400
Xing,2022             GLP-1 + Metformin              Metformin -0.5600 0.7800
Elkind-Hirsch,2022               GLP-1               Placebo -0.8000 0.3000
Ma,2021               GLP-1 + Metformin              Metformin -0.9000 0.9300
Soldat-Stanković,2022        Metformin Myoinositol + Folic acid 0.4600 0.6100
Tao,2021                         GLP-1              Metformin 1.3600 0.0800
Zheng,2019                       GLP-1              Metformin -0.6100 0.3900
Shokrpour,2019               Metformin Myoinositol + Folic acid 0.3000 0.2700
```

Zahra,2016	Metformin	Placebo	0.9000	0.8200
Ravn,2022	Metformin	Myoinositol + Folic acid	-14.0700	7.3100
Zheng,2017	GLP-1	Metformin	-0.6100	0.5000
Jensterle,2016	GLP-1	GLP-1 + Metformin	-0.3000	0.6300
Jensterle,2015	GLP-1	Metformin	-0.2000	1.2200

Number of treatment arms (by study):

	narms
Wen,2023	2
Xing,2022	2
Elkind-Hirsch,2022	2
Ma,2021	2
Soldat-Stanković,2022	2
Tao,2021	2
Zheng,2019	2
Shokrpour,2019	2
Zahra,2016	2
Ravn,2022	2
Zheng,2017	2
Jensterle,2016	2
Jensterle,2015	2

Results (random effects model):

	treat1	treat2	MD	95%-CI
Wen,2023	GLP-1 + Metformin	Metformin	-0.5094	[-1.8525; 0.8337]
Xing,2022	GLP-1 + Metformin	Metformin	-0.5094	[-1.8525; 0.8337]
Elkind-Hirsch,2022	GLP-1	Placebo	-0.1955	[-2.0675; 1.6765]
Ma,2021	GLP-1 + Metformin	Metformin	-0.5094	[-1.8525; 0.8337]
Soldat-Stanković,2022	Metformin	Myoinositol + Folic acid	0.1666	[-1.5661; 1.8993]
Tao,2021	GLP-1	Metformin	-0.2510	[-1.3741; 0.8721]
Zheng,2019	GLP-1	Metformin	-0.2510	[-1.3741; 0.8721]
Shokrpour,2019	Metformin	Myoinositol + Folic acid	0.1666	[-1.5661; 1.8993]
Zahra,2016	Metformin	Placebo	0.0555	[-1.8715; 1.9825]
Ravn,2022	Metformin	Myoinositol + Folic acid	0.1666	[-1.5661; 1.8993]
Zheng,2017	GLP-1	Metformin	-0.2510	[-1.3741; 0.8721]
Jensterle,2016	GLP-1	GLP-1 + Metformin	0.2584	[-1.2996; 1.8164]
Jensterle,2015	GLP-1	Metformin	-0.2510	[-1.3741; 0.8721]

Number of studies: k = 13
Number of pairwise comparisons: m = 13
Number of treatments: n = 5
Number of designs: d = 6

Random effects model

Treatment estimate (sm = 'MD', comparison: other treatments vs 'Placebo'):

	MD	95%-CI	z	p-value
GLP-1	-0.1955	[-2.0675; 1.6765]	-0.20	0.8378
GLP-1 + Metformin	-0.4538	[-2.7222; 1.8145]	-0.39	0.6950
Metformin	0.0555	[-1.8715; 1.9825]	0.06	0.9550
Myoinositol + Folic acid	-0.1111	[-2.7025; 2.4804]	-0.08	0.9331
Placebo	.	.	.	.

Quantifying heterogeneity / inconsistency:
tau^2 = 1.3772; tau = 1.1736; I^2 = 86.4% [76.9%; 92.0%]

Tests of heterogeneity (within designs) and inconsistency (between designs):

	Q	d.f.	p-value
Total	66.26	9	< 0.0001
Within designs	43.83	7	< 0.0001
Between designs	22.43	2	< 0.0001

Details of network meta-analysis methods:
- Frequentist graph-theoretical approach
- DerSimonian-Laird estimator for tau^2
- Calculation of I^2 based on Q

[[10]]
Original data:

	treat1	treat2	TE	seTE
Wen,2023	GLP-1 + Metformin	Metformin	-0.6700	0.1400
Xing,2022	GLP-1 + Metformin	Metformin	-0.5600	0.7800
Elkind-Hirsch,2022	GLP-1	Placebo	-0.8000	0.3000
Ma,2021	GLP-1 + Metformin	Metformin	-0.9000	0.9300
Soldat-Stanković,2022	Metformin	Myoinositol + Folic acid	0.4600	0.6100
Tao,2021	GLP-1	Metformin	1.3600	0.0800
Zheng,2019	GLP-1	Metformin	-0.6100	0.3900
Shokrpour,2019	Metformin	Myoinositol + Folic acid	0.3000	0.2700
Jensterle,2017	GLP-1	GLP-1 + Metformin	1.4000	1.2100
Ravn,2022	Metformin	Myoinositol + Folic acid	-14.0700	7.3100
Zheng,2017	GLP-1	Metformin	-0.6100	0.5000
Jensterle,2016	GLP-1	GLP-1 + Metformin	-0.3000	0.6300
Jensterle,2015	GLP-1	Metformin	-0.2000	1.2200

Number of treatment arms (by study):
narms

Wen,2023	2
Xing,2022	2
Elkind-Hirsch,2022	2
Ma,2021	2
Soldat-Stanković,2022	2
Tao,2021	2
Zheng,2019	2
Shokrpour,2019	2
Jensterle,2017	2
Ravn,2022	2
Zheng,2017	2
Jensterle,2016	2
Jensterle,2015	2

Results (random effects model):

	treat1	treat2	MD	95%-CI
Wen,2023	GLP-1 + Metformin	Metformin	-0.5755	[-1.7844; 0.6333]
Xing,2022	GLP-1 + Metformin	Metformin	-0.5755	[-1.7844; 0.6333]
Elkind-Hirsch,2022	GLP-1	Placebo	-0.8000	[-3.0020; 1.4020]
Ma,2021	GLP-1 + Metformin	Metformin	-0.5755	[-1.7844; 0.6333]
Soldat-Stanković,2022	Metformin	Myoinositol + Folic acid	0.1914	[-1.4257; 1.8085]
Tao,2021	GLP-1	Metformin	-0.0151	[-1.0870; 1.0567]
Zheng,2019	GLP-1	Metformin	-0.0151	[-1.0870; 1.0567]
Shokrpour,2019	Metformin	Myoinositol + Folic acid	0.1914	[-1.4257; 1.8085]
Jensterle,2017	GLP-1	GLP-1 + Metformin	0.5604	[-0.7838; 1.9046]
Ravn,2022	Metformin	Myoinositol + Folic acid	0.1914	[-1.4257; 1.8085]
Zheng,2017	GLP-1	Metformin	-0.0151	[-1.0870; 1.0567]
Jensterle,2016	GLP-1	GLP-1 + Metformin	0.5604	[-0.7838; 1.9046]
Jensterle,2015	GLP-1	Metformin	-0.0151	[-1.0870; 1.0567]

Number of studies: k = 13
Number of pairwise comparisons: m = 13
Number of treatments: n = 5
Number of designs: d = 5

Random effects model

Treatment estimate (sm = 'MD', comparison: other treatments vs 'Placebo'):

	MD	95%-CI	z	p-value
GLP-1	-0.8000	[-3.0020; 1.4020]	-0.71	0.4764
GLP-1 + Metformin	-1.3604	[-3.9402; 1.2195]	-1.03	0.3014
Metformin	-0.7849	[-3.2338; 1.6641]	-0.63	0.5299
Myoinositol + Folic acid	-0.9762	[-3.9110; 1.9585]	-0.65	0.5144

Placebo . . . .

Quantifying heterogeneity / inconsistency:
tau^2 = 1.1722; tau = 1.0827; I^2 = 83.8% [71.7%; 90.7%]

Tests of heterogeneity (within designs) and inconsistency (between designs):

	Q	d.f.	p-value
Total	55.42	9	< 0.0001
Within designs	45.38	8	< 0.0001
Between designs	10.03	1	0.0015

Details of network meta-analysis methods:
- Frequentist graph-theoretical approach
- DerSimonian-Laird estimator for tau^2
- Calculation of I^2 based on Q

[[11]]
Original data:

	treat1	treat2	TE	seTE
Wen,2023	GLP-1 + Metformin	Metformin	-0.6700	0.1400
Xing,2022	GLP-1 + Metformin	Metformin	-0.5600	0.7800
Elkind-Hirsch,2022	GLP-1	Placebo	-0.8000	0.3000
Ma,2021	GLP-1 + Metformin	Metformin	-0.9000	0.9300
Soldat-Stanković,2022	Metformin	Myoinositol + Folic acid	0.4600	0.6100
Tao,2021	GLP-1	Metformin	1.3600	0.0800
Zheng,2019	GLP-1	Metformin	-0.6100	0.3900
Shokrpour,2019	Metformin	Myoinositol + Folic acid	0.3000	0.2700
Jensterle,2017	GLP-1	GLP-1 + Metformin	1.4000	1.2100
Zahra,2016	Metformin	Placebo	0.9000	0.8200
Zheng,2017	GLP-1	Metformin	-0.6100	0.5000
Jensterle,2016	GLP-1	GLP-1 + Metformin	-0.3000	0.6300
Jensterle,2015	GLP-1	Metformin	-0.2000	1.2200

Number of treatment arms (by study):

	narms
Wen,2023	2
Xing,2022	2
Elkind-Hirsch,2022	2
Ma,2021	2
Soldat-Stanković,2022	2
Tao,2021	2
Zheng,2019	2
Shokrpour,2019	2

Jensterle,2017 2
Zahra,2016 2
Zheng,2017 2
Jensterle,2016 2
Jensterle,2015 2

Results (random effects model):

treat1 treat2 MD 95%-CI
Wen,2023 GLP-1 + Metformin Metformin -0.6303 [-1.8583; 0.5977]
Xing,2022 GLP-1 + Metformin Metformin -0.6303 [-1.8583; 0.5977]
Elkind-Hirsch,2022 GLP-1 Placebo -0.1710 [-1.9567; 1.6147]
Ma,2021 GLP-1 + Metformin Metformin -0.6303 [-1.8583; 0.5977]

Tao,2021 GLP-1 Metformin -0.1656 [-1.2114; 0.8801]
Zheng,2019 GLP-1 Metformin -0.1656 [-1.2114; 0.8801]
Shokrpour,2019 Metformin Myoinositol + Folic acid 0.3718 [-1.2930; 2.036
Jensterle,2017 GLP-1 GLP-1 + Metformin 0.4646 [-0.8893; 1.8186]
Zahra,2016 Metformin Placebo -0.0053 [-1.8454; 1.8347]
Zheng,2017 GLP-1 Metformin -0.1656 [-1.2114; 0.8801]
Jensterle,2016 GLP-1 GLP-1 + Metformin 0.4646 [-0.8893; 1.8186]
Jensterle,2015 GLP-1 Metformin -0.1656 [-1.2114; 0.8801]
Soldat-Stanković,2022 Metformin Myoinositol + Folic acid 0.3718 [-1.2930; 2.0366]

6]

Number of studies: k = 13
Number of pairwise comparisons: m = 13
Number of treatments: n = 5
Number of designs: d = 6

Random effects model

Treatment estimate (sm = 'MD', comparison: other treatments vs 'Placebo'):

	MD	95%-CI	z	p-value
GLP-1	-0.1710	[-1.9567; 1.6147]	-0.19	0.8511
GLP-1 + Metformin	-0.6356	[-2.7429; 1.4716]	-0.59	0.5544
Metformin	-0.0053	[-1.8454; 1.8347]	-0.01	0.9955
Myoinositol + Folic acid	-0.3771	[-2.8585; 2.1042]	-0.30	0.7658
Placebo	.	.	.	.

Quantifying heterogeneity / inconsistency:

tau^2 = 1.2358; tau = 1.1116; I^2 = 85.6% [75.3%; 91.6%]

Tests of heterogeneity (within designs) and inconsistency (between designs):

	Q	d.f.	p-value
Total	62.46	9	< 0.0001

Within designs 41.51 7 < 0.0001
Between designs 20.96 2 < 0.0001

Details of network meta-analysis methods:
- Frequentist graph-theoretical approach
- DerSimonian-Laird estimator for tau^2
- Calculation of I^2 based on Q

[[12]]
Original data:

	treat1	treat2	TE	seTE
Wen,2023	GLP-1 + Metformin	Metformin	-0.6700	0.1400
Xing,2022	GLP-1 + Metformin	Metformin	-0.5600	0.7800
Elkind-Hirsch,2022	GLP-1	Placebo	-0.8000	0.3000
Ma,2021	GLP-1 + Metformin	Metformin	-0.9000	0.9300
Soldat-Stanković,2022	Metformin	Myoinositol + Folic acid	0.4600	0.6100
Tao,2021	GLP-1	Metformin	1.3600	0.0800
Zheng,2019	GLP-1	Metformin	-0.6100	0.3900
Shokrpour,2019	Metformin	Myoinositol + Folic acid	0.3000	0.2700
Jensterle,2017	GLP-1	GLP-1 + Metformin	1.4000	1.2100
Zahra,2016	Metformin	Placebo	0.9000	0.8200
Ravn,2022	Metformin	Myoinositol + Folic acid	-14.0700	7.3100
Jensterle,2016	GLP-1	GLP-1 + Metformin	-0.3000	0.6300
Jensterle,2015	GLP-1	Metformin	-0.2000	1.2200

Number of treatment arms (by study):

	narms
Wen,2023	2
Xing,2022	2
Elkind-Hirsch,2022	2
Ma,2021	2
Soldat-Stanković,2022	2
Tao,2021	2
Zheng,2019	2
Shokrpour,2019	2
Jensterle,2017	2
Zahra,2016	2
Ravn,2022	2
Jensterle,2016	2
Jensterle,2015	2

Results (random effects model):

	treat1	treat2	MD	95%-CI
Wen,2023	GLP-1 + Metformin	Metformin	-0.5930	[-1.8320; 0.6459]
Xing,2022	GLP-1 + Metformin	Metformin	-0.5930	[-1.8320; 0.6459]
Elkind-Hirsch,2022	GLP-1	Placebo	-0.1282	[-1.9229; 1.6665]
Ma,2021	GLP-1 + Metformin	Metformin	-0.5930	[-1.8320; 0.6459]
Soldat-Stanković,2022	Metformin	Myoinositol + Folic acid	0.1843	[-1.4666; 1.8353]
Tao,2021	GLP-1	Metformin	-0.0601	[-1.2212; 1.1011]
Zheng,2019	GLP-1	Metformin	-0.0601	[-1.2212; 1.1011]
Shokrpour,2019	Metformin	Myoinositol + Folic acid	0.1843	[-1.4666; 1.8353]
Jensterle,2017	GLP-1	GLP-1 + Metformin	0.5330	[-0.8586; 1.9245]
Zahra,2016	Metformin	Placebo	-0.0681	[-1.9295; 1.7932]
Ravn,2022	Metformin	Myoinositol + Folic acid	0.1843	[-1.4666; 1.8353]
Jensterle,2016	GLP-1	GLP-1 + Metformin	0.5330	[-0.8586; 1.9245]
Jensterle,2015	GLP-1	Metformin	-0.0601	[-1.2212; 1.1011]

Number of studies: k = 13
Number of pairwise comparisons: m = 13
Number of treatments: n = 5
Number of designs: d = 6

Random effects model

Treatment estimate (sm = 'MD', comparison: other treatments vs 'Placebo'):

	MD	95%-CI	z	p-value
GLP-1	-0.1282	[-1.9229; 1.6665]	-0.14	0.8887
GLP-1 + Metformin	-0.6612	[-2.7684; 1.4461]	-0.61	0.5386
Metformin	-0.0681	[-1.9295; 1.7932]	-0.07	0.9428
Myoinositol + Folic acid	-0.2524	[-2.7404; 2.2356]	-0.20	0.8424
Placebo	.	.	.	.

Quantifying heterogeneity / inconsistency:
tau^2 = 1.2306; tau = 1.1093; I^2 = 83.1% [70.4%; 90.4%]

Tests of heterogeneity (within designs) and inconsistency (between designs):

	Q	d.f.	p-value
Total	53.29	9	< 0.0001
Within designs	31.52	7	< 0.0001
Between designs	21.78	2	< 0.0001

Details of network meta-analysis methods:
- Frequentist graph-theoretical approach
- DerSimonian-Laird estimator for tau^2
- Calculation of I^2 based on Q

[[13]]
Original data:

	treat1	treat2	TE	seTE
Wen,2023	GLP-1 + Metformin	Metformin	-0.6700	0.1400
Xing,2022	GLP-1 + Metformin	Metformin	-0.5600	0.7800
Elkind-Hirsch,2022	GLP-1	Placebo	-0.8000	0.3000
Ma,2021	GLP-1 + Metformin	Metformin	-0.9000	0.9300
Soldat-Stanković,2022	Metformin	Myoinositol + Folic acid	0.4600	0.6100
Tao,2021	GLP-1	Metformin	1.3600	0.0800
Zheng,2019	GLP-1	Metformin	-0.6100	0.3900
Shokrpour,2019	Metformin	Myoinositol + Folic acid	0.3000	0.2700
Jensterle,2017	GLP-1	GLP-1 + Metformin	1.4000	1.2100
Zahra,2016	Metformin	Placebo	0.9000	0.8200
Ravn,2022	Metformin	Myoinositol + Folic acid	-14.0700	7.3100
Zheng,2017	GLP-1	Metformin	-0.6100	0.5000
Jensterle,2015	GLP-1	Metformin	-0.2000	1.2200

Number of treatment arms (by study):

	narms
Wen,2023	2
Xing,2022	2
Elkind-Hirsch,2022	2
Ma,2021	2
Soldat-Stanković,2022	2
Tao,2021	2
Zheng,2019	2
Shokrpour,2019	2
Jensterle,2017	2
Zahra,2016	2
Ravn,2022	2
Zheng,2017	2
Jensterle,2015	2

Results (random effects model):

	treat1	treat2	MD	95%-CI
Wen,2023	GLP-1 + Metformin	Metformin	-0.8245	[-2.1697; 0.5206]
Xing,2022	GLP-1 + Metformin	Metformin	-0.8245	[-2.1697; 0.5206]
Elkind-Hirsch,2022	GLP-1	Placebo	-0.1197	[-1.9202; 1.6808]
Ma,2021	GLP-1 + Metformin	Metformin	-0.8245	[-2.1697; 0.5206]
Soldat-Stanković,2022	Metformin	Myoinositol + Folic acid	0.1818	[-1.4810; 1.8446]
Tao,2021	GLP-1	Metformin	-0.0440	[-1.1482; 1.0602]
Zheng,2019	GLP-1	Metformin	-0.0440	[-1.1482; 1.0602]

```
Shokrpour,2019              Metformin Myoinositol + Folic acid 0.1818 [-1.4810; 1.8446]
Jensterle,2017                  GLP-1        GLP-1 + Metformin 0.7805 [-0.8363; 2.3973]
Zahra,2016                 Metformin                    Placebo -0.0757 [-1.9356; 1.7842]
Ravn,2022                  Metformin Myoinositol + Folic acid 0.1818 [-1.4810; 1.8446]
Zheng,2017                      GLP-1                Metformin -0.0440 [-1.1482; 1.0602]
Jensterle,2015                  GLP-1                Metformin -0.0440 [-1.1482; 1.0602]

Number of studies: k = 13
Number of pairwise comparisons: m = 13
Number of treatments: n = 5
Number of designs: d = 6

Random effects model

Treatment estimate (sm = 'MD', comparison: other treatments vs 'Placebo'):
                              MD            95%-CI     z p-value
GLP-1                    -0.1197 [-1.9202; 1.6808] -0.13 0.8963
GLP-1 + Metformin        -0.9002 [-3.1417; 1.3413] -0.79 0.4312
Metformin                -0.0757 [-1.9356; 1.7842] -0.08 0.9364
Myoinositol + Folic acid -0.2575 [-2.7523; 2.2373] -0.20 0.8397
Placebo                        .                  .     .       .

Quantifying heterogeneity / inconsistency:
tau^2 = 1.2514; tau = 1.1187; I^2 = 83.7% [71.5%; 90.7%]

Tests of heterogeneity (within designs) and inconsistency (between designs):
                    Q d.f. p-value
Total           55.15    9 < 0.0001
Within designs  43.83    7 < 0.0001
Between designs 11.32    2  0.0035

Details of network meta-analysis methods:
- Frequentist graph-theoretical approach
- DerSimonian-Laird estimator for tau^2
- Calculation of I^2 based on Q

[[14]]
Original data:

                              treat1              treat2       TE   seTE
Wen,2023              GLP-1 + Metformin            Metformin -0.6700 0.1400
Xing,2022             GLP-1 + Metformin            Metformin -0.5600 0.7800
Elkind-Hirsch,2022                GLP-1              Placebo -0.8000 0.3000
Ma,2021               GLP-1 + Metformin            Metformin -0.9000 0.9300
```

Soldat-Stanković,2022	Metformin	Myoinositol + Folic acid	0.4600	0.6100
Tao,2021	GLP-1	Metformin	1.3600	0.0800
Zheng,2019	GLP-1	Metformin	-0.6100	0.3900
Shokrpour,2019	Metformin	Myoinositol + Folic acid	0.3000	0.2700
Jensterle,2017	GLP-1	GLP-1 + Metformin	1.4000	1.2100
Zahra,2016	Metformin	Placebo	0.9000	0.8200
Ravn,2022	Metformin	Myoinositol + Folic acid	-14.0700	7.3100
Zheng,2017	GLP-1	Metformin	-0.6100	0.5000
Jensterle,2016	GLP-1	GLP-1 + Metformin	-0.3000	0.6300

Number of treatment arms (by study):

	narms
Wen,2023	2
Xing,2022	2
Elkind-Hirsch,2022	2
Ma,2021	2
Soldat-Stanković,2022	2
Tao,2021	2
Zheng,2019	2
Shokrpour,2019	2
Jensterle,2017	2
Zahra,2016	2
Ravn,2022	2
Zheng,2017	2
Jensterle,2016	2

Results (random effects model):

	treat1	treat2	MD	95%-CI
Wen,2023	GLP-1 + Metformin	Metformin	-0.6333	[-1.9041; 0.6376]
Xing,2022	GLP-1 + Metformin	Metformin	-0.6333	[-1.9041; 0.6376]
Elkind-Hirsch,2022	GLP-1	Placebo	-0.1630	[-2.0127; 1.6867]
Ma,2021	GLP-1 + Metformin	Metformin	-0.6333	[-1.9041; 0.6376]
Soldat-Stanković,2022	Metformin	Myoinositol + Folic acid	0.1720	[-1.5364; 1.8803]
Tao,2021	GLP-1	Metformin	-0.1653	[-1.3067; 0.9761]
Zheng,2019	GLP-1	Metformin	-0.1653	[-1.3067; 0.9761]
Shokrpour,2019	Metformin	Myoinositol + Folic acid	0.1720	[-1.5364; 1.8803]
Jensterle,2017	GLP-1	GLP-1 + Metformin	0.4680	[-0.9449; 1.8809]
Zahra,2016	Metformin	Placebo	0.0023	[-1.9063; 1.9108]
Ravn,2022	Metformin	Myoinositol + Folic acid	0.1720	[-1.5364; 1.8803]
Zheng,2017	GLP-1	Metformin	-0.1653	[-1.3067; 0.9761]
Jensterle,2016	GLP-1	GLP-1 + Metformin	0.4680	[-0.9449; 1.8809]

Number of studies: k = 13

Number of pairwise comparisons: m = 13
Number of treatments: n = 5
Number of designs: d = 6

Random effects model

Treatment estimate (sm = 'MD', comparison: other treatments vs 'Placebo'):

	MD	95%-CI	z	p-value
GLP-1	-0.1630	[-2.0127; 1.6867]	-0.17	0.8629
GLP-1 + Metformin	-0.6310	[-2.8033; 1.5412]	-0.57	0.5691
Metformin	0.0023	[-1.9063; 1.9108]	0.00	0.9981
Myoinositol + Folic acid	-0.1697	[-2.7312; 2.3918]	-0.13	0.8967
Placebo	.	.	.	.

Quantifying heterogeneity / inconsistency:
tau^2 = 1.3327; tau = 1.1544; I^2 = 86.2% [76.4%; 91.9%]

Tests of heterogeneity (within designs) and inconsistency (between designs):

	Q	d.f.	p-value
Total	65.06	9	< 0.0001
Within designs	44.00	7	< 0.0001
Between designs	21.06	2	< 0.0001

Details of network meta-analysis methods:
- Frequentist graph-theoretical approach
- DerSimonian-Laird estimator for tau^2
- Calculation of I^2 based on Q

=== Heterogeneity & Inconsistency ===
[1] 0.8492577
Q statistics to assess homogeneity / consistency

Design-specific decomposition of within-designs Q statistic

Design	Q	df	p-value
GLP-1 vs Metformin	39.82	3	< 0.0001
Metformin vs Myoinositol + Folic acid	3.93	2	0.1400
GLP-1 vs GLP-1 + Metformin	1.55	1	0.2127
GLP-1 + Metformin vs Metformin	0.08	2	0.9603

Between-designs Q statistic after detaching of single designs
(influential designs have p-value markedly different from < 0.0001)

Detached design	Q	df	p-value
GLP-1 vs Metformin	1.09	1	0.2968
Placebo vs GLP-1	10.03	1	0.0015
Placebo vs Metformin	10.03	1	0.0015
GLP-1 vs GLP-1 + Metformin	11.17	1	0.0008
GLP-1 + Metformin vs Metformin	11.17	1	0.0008

Q statistic to assess consistency under the assumption of
a full design-by-treatment interaction random effects model

11. PRISMA Checklist

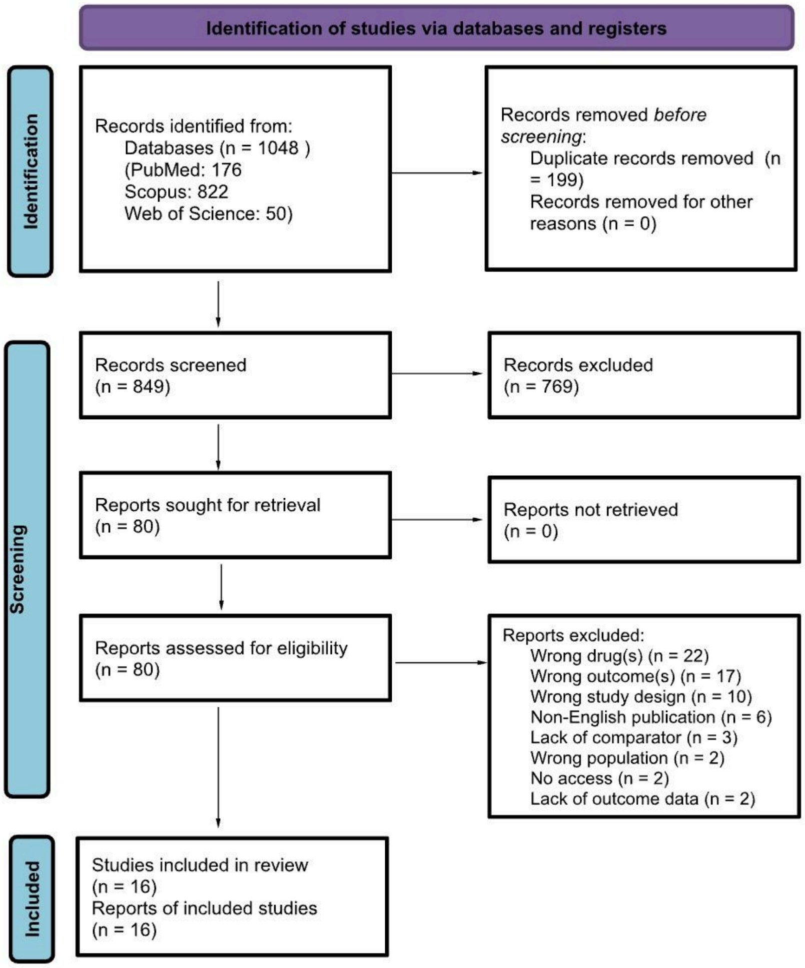

Figure 9: PRISMA Checklist
